# Supplementary material for: Extent and nature of duplication in PROSPERO using COVID-19-related registrations: a retrospective investigation and survey
Source: BMJ Open. 2022 Dec 1;12(12):e061862. doi: 10.1136/bmjopen-2022-061862 (PMC9716408; doi:10.1136/bmjopen-2022-061862)
Supplement: Supplementary data [file bmjopen-2022-061862supp001.pdf]

Appendix A

Search Terms:

'(((coronavirus or corona-virus) AND (wuhan or beijing or shanghai or Italy or South-Korea or korea or China or Chinese or 2019-nCoV or nCoV or COVID-19 or Covid19 or SARS-CoV* or SARSCov2 or ncov)) OR (pneumonia AND Wuhan) or "COVID-19" or "2019-nCoV" or "SARS-CoV" or SARSCOV2 or 2019-nCov or "2019 coronavirus" or "2019 corona virus" or covid19 or ncov OR "novel corona virus" or "new corona virus" or "nouveau corona virus" or "2019 corona virus" OR "novel coronavirus" or "new coronavirus" or "nouveau coronavirus" or "2019 coronavirus" or "Long Covid") NOT Animal:DB'

Appendix B

Data Extraction

Table 1. *Data extraction template for identifying duplicate reviews*

Record number	Research area	Date submitted	Date registered	Is the PICOS similar/identical to existing review?	Answer to screening question? *	Is the review funded?	Have authors acknowledged similar reviews?	Authors reason for duplication?	Is there a reason for duplication from our perspective?

**Screening questions before authors submit a PROSPERO registration ask whether their review is not similar to an existing review, sufficiently different, or similar to an existing review, but repetition is needed*

Appendix C

COVID-19 Systematic Review Duplication in PROSPERO Questionnaire

The survey can be entirely anonymous. But it would be useful for us to be able to link your responses to the PROSPERO record, so you can choose to include your personal information. If you do provide your identifying ID number, this will not be included or used in any future publications and it will not affect the status of your PROSPERO registration.

This survey has been granted ethical approval. If you choose to include your PROSPERO registration number, your data will be handled in compliance with GDPR and the UK Data Protection Act (2018). For more information, please visit the links on the University of York's policy on Data Protection Legislation, GDPR compliant research and the general privacy notice.

Q1 What is your PROSPERO registration number

____

About your COVID-19 related systematic review

Q2 Have you conducted a systematic review before?

- Yes (1)
- No (2)
- Other (3) ____

Q3 What research area was your systematic review looking at?

- Epidemiology (1)
- Transmission (2)
- Treatments (3)
- Other (4) ____

Q4 Why did you conduct your systematic review on a COVID-19 related topic?

- To inform future research that you had planned (such as a primary research study) (1)
- To inform funders/policy makers (2)
- For personal/departmental interest (3)
- Other (4) ____

Q5 Please provide details of the country in which the review is being carried out. For multi-national collaborations, give details of all the countries involved.

____

Q6 Is your review funded

- Yes (1)
- No (2)
- Other (3) ____

Display This Question:

If Is your review funded != No

Q7 If your review is funded, please provide details of the individuals, organizations, groups or other legal entities who take responsibility for initiating, managing, sponsoring and/or financing the review

____

Q8 Do you intend to publish or disseminate the results of your systematic review?

- Yes (1)
- No (2)
- Maybe (3)
- Other (4) ____

Display This Question:

If Do you intend to publish or disseminate the results of your systematic review? = No

Q9 Why did you decide not to publish the results of your systematic review?

____

Q10 Is there any other information that you wish to provide about your systematic review?

____

The following questions were specific to the registrant's response to the triage questions at PROSPERO submission

Not Similar Sub-Questions

When registering, users are prompted to search PROSPERO and check whether a similar systematic review has been registered. Some duplication, intended with good reason (e.g. the existing one is out of date or used suboptimal methods), is justifiable. When registering, you indicated that your review was not similar to an existing review

Q12 To help us understand this further, please indicate which statement applies most to your situation.

- I searched PROSPERO prior to registration and identified some/all of the similar records (1)
- I searched PROSPERO prior to registration but did not identify the similar records (2)
- I did not search PROSPERO prior to registration (3)

Display This Question:

If To help us understand this further, please indicate which statement applies most to your situation. = I searched PROSPERO prior to registration and identified some/all of the similar records

Q13 Please could you tell us more about the reasons you conducted a review similar to those which you identified?

____

Display This Question:

If To help us understand this further, please indicate which statement applies most to your situation. = I searched PROSPERO prior to registration but did not identify the similar records

Q14 Please could you tell us more about how you searched PROSPERO, including the search terms you used. This could help us to improve our guidance about how to search PROSPERO in the future.

____

Display This Question:

If To help us understand this further, please indicate which statement applies most to your situation. = I did not search PROSPERO prior to registration

Q15 Please could you tell us more about the reasons you did not search PROSPERO before registration?

____

Sufficiently Different Sub-Questions

When registering, users are prompted to search PROSPERO and check whether a similar systematic review has been registered. Some duplication, intended with good reason (e.g. the existing one is out of date or used suboptimal methods), is justifiable. When registering, you indicated that your review was similar to an existing review but sufficiently different to justify this review.

Q12 To help us understand this further, please indicate which statement applies most to your situation.

- I searched PROSPERO and identified some/ all of the existing reviews that were similar to my own. (1)
- Other (2)

Display This Question:

If To help us understand this further, please indicate which statement applies most to your situation. = I searched PROSPERO and identified some/ all of the existing reviews that were similar to my own.

Q13 For each of the similar reviews you identified, please could you tell us more about the reasons why your review is sufficiently different.

____

Display This Question:

If To help us understand this further, please indicate which statement applies most to your situation. = Other

Q14 Please could you tell us whether you searched PROSPERO, and if you did, please could you explain more about how you searched PROSPERO, including the search terms you used. This could help us to improve our guidance about how to search PROSPERO in the future.

____

Repetition Needed Sub-Questions

When registering, users are prompted to search PROSPERO and check whether a similar systematic review has been registered. Some duplication, intended with good reason (e.g. the existing one is out of date or used suboptimal methods), is justifiable. When registering, you indicated that your review was similar to an existing review but repetition was needed.

Q12 To help us understand this further, please indicate which statement applies most to your situation.

- I searched PROSPERO and identified some/ all of the existing reviews that were similar to my own. (1)
- Other (2)

Display This Question:

If To help us understand this further, please indicate which statement applies most to your situation. = I searched PROSPERO and identified some/ all of the existing reviews that were similar to my own.

Q13 For each of the similar reviews you identified, please could you tell us more about the reasons why repetition was needed.

____

Display This Question:

If To help us understand this further, please indicate which statement applies most to your situation. = Other

Q14 Please could you tell us whether you searched PROSPERO, and if you did, please could you explain more about how you searched PROSPERO, including the search terms you used. This could help us to improve our guidance about how to search PROSPERO in the future.

____

Appendix D

Research area	Date submitted	Date registered	Answer to screening question?	Have authors acknowledged similar reviews?	Authors reason for duplication?	Is there reason for duplication from our perspective?	Is the review funded?
**Cancer**	17/04/20	21/04/20	-	-	-	-	None
**Cancer**	22/04/20	22/04/20	NS	No	No	Perhaps, submitted one day after last was published	None
**Cardiovascular Disease**	14/04/20	20/04/20	-	-	-	-	None
**Cardiovascular Disease**	31/05/20	01/06/20	NS	No	No	No	National Natural Science Foundation of China
**Children**	15/04/20	21/04/20	-	-	-	-	None
**Children**	18/04/20	20/04/20	-	-	-	-	Nanchang University

Research area	Date submitted	Date registered	Answer to screening question?	Have authors acknowledged similar reviews?	Authors reason for duplication?	Is there reason for duplication from our perspective?	Is the review funded?
**Children**	02/08/20	06/08/20	RN*	No	-	Potentially. Four months since previous systematic review	None
**Comorbidities: Impact on Mortality**	24/03/20	03/04/20	-	-	-	-	None
**Comorbidities: Impact on Mortality**	22/05/20	28/05/20		No	None	No	None
**Comorbidities: Impact on Mortality**	29/10/20	30/10/20	NS*	No	None	Perhaps- to update evidence (5 months after last review).	None
**Comorbidities: Prevalence**	27/03/20	06/04/20	-	-	-	-	Human Resources Development Operational Programme Grant
**Comorbidities: Prevalence**	26/04/20	28/04/20	NS*	No	No	No	None

Research area	Date submitted	Date registered	Answer to screening question?	Have authors acknowledged similar reviews?	Authors reason for duplication?	Is there reason for duplication from our perspective?	Is the review funded?
**Comorbidities: Severe Outcomes and Death**	26/02/20	17/03/20	-	-	-	-	National Key R&D Program of China.
**Comorbidities: Severe Outcomes and Death**	26/03/20	16/04/20	SD	Yes	Yes: no risk of bias in CRD42020170315	Yes, CRD42020170315 do not report methods of quality assessment).	None
**Comorbidities: Severe Outcomes and Death**	31/03/20	30/04/20	NS	No	None	No	None
**Comorbidities: Severe Outcomes and Death**	13/04/20	16/04/20	NS	No	None	No	Second Affiliated Hospital of Nanchang University
**COVID-19 in Healthcare Workers**	06/04/20	15/04/20	--	-	-	-	None
**COVID-19 in Healthcare Workers**	27/04/20	28/04/20	NS	None	No	No	None
**Diabetes**	28/04/20	04/05/20	-	-	-	-	None

Research area	Date submitted	Date registered	Answer to screening question?	Have authors acknowledged similar reviews?	Authors reason for duplication?	Is there reason for duplication from our perspective?	Is the review funded?
**Diabetes**	30/06/20	30/06/20	NS	No	None	No	Natural Science Foundation of Tianjan and Tianjan Education Committee
**Diabetes**	05/10/20	06/10/20	NS	No	None	Perhaps update of review potentially needed	None
**Echocardiographic Findings**	18/08/20	19/08/20	-	-	-	-	None
**Echocardiographic Findings**	19/09/20	22/09/20	NS	No	No	No	None
**Face Masks**	29/09/20	29/09/20	-	-	-	-	None
**Face Masks**	19/10/20	28/10/20	SD	No	No	Yes. Living systematic review	None
**General Risk Factors: Impact on Mortality**	26/03/20	03/04/20	-	-	-	-	None

Research area	Date submitted	Date registered	Answer to screening question?	Have authors acknowledged similar reviews?	Authors reason for duplication?	Is there reason for duplication from our perspective?	Is the review funded?
**General Risk Factors: Impact on Mortality**	04/04/20	09/04/20	NS	None	No	Potentially- only one day after previous record was registered.	Wellcome Trust
**General Risk Factors: Impact on Poor Outcomes and Mortality**	27/02/20	03/04/20	-	-	-	-	National Natural Science Foundation
**General Risk Factors: Impact on Poor Outcomes and Mortality**	15/03/20	27/03/20	-	-	-	-	None
**General Risk Factors: Impact on Poor Outcomes and Mortality**	18/03/20	23/03/20	NS	None	No	No	Chinese Natural Science Foundation and the Training Program of Shanghai Health and Family Planning System
**General Risk Factors: Impact on Poor Outcomes and Mortality**	28/03/20	09/04/20	SD	Yes	Yes, provide additional analysis (regression)	Yes	None

Research area	Date submitted	Date registered	Answer to screening question?	Have authors acknowledged similar reviews?	Authors reason for duplication?	Is there reason for duplication from our perspective?	Is the review funded?
**General Risk Factors: Impact on Poor Outcomes and Mortality**	12/04/20	16/04/20	NS	No	None	No	None
**General Risk Factors: Impact on Poor Outcomes and Mortality**	14/04/20	17/05/20	NS*	No	None	None	None
**General Risk Factors: Impact on Poor Outcomes and Mortality**	20/04/20	21/04/20	RN	No	None	None	None
**General Risk Factors: Impact on Poor Outcomes and Mortality**	06/05/20	06/05/20	SD	No	None	No	Department of Epidemiology, College of Public Health, University of Nebraska Medical
**General Risk Factors: Impact on Poor Outcomes and Mortality**	11/05/20	12/05/20	NS	No	None	No	None
**Long COVID**	15/10/20	23/10/20	-	-	-	-	None

Research area	Date submitted	Date registered	Answer to screening question?	Have authors acknowledged similar reviews?	Authors reason for duplication?	Is there reason for duplication from our perspective?	Is the review funded?
**Long COVID**	31/10/20	02/11/20	SD	No	No	No	None
**Manifestations: Taste and Smell**	22/05/20	29/05/20	-	-	-	-	None
**Manifestations: Taste and Smell**	20/06/20	25/06/20	NS	No	None	None	Health Commission of Hunan Province and Human Pharmaceutical Association
**Mental Health in Healthcare Workers**	31/07/20	03/08/20	-	-	-	-	National Council for Scientific and Technological Development - CNPq, Brazilian Government
**Mental Health in Healthcare Workers**	19/10/20	22/10/20	NULL	No	No	No	None
**Neurological Manifestations**	22/05/20	01/06/20	-	-	-	-	None
**Neurological Manifestations**	02/06/20	02/06/20	NS	No	None	Perhaps, submitted one day after previous was published	None

Research area	Date submitted	Date registered	Answer to screening question?	Have authors acknowledged similar reviews?	Authors reason for duplication?	Is there reason for duplication from our perspective?	Is the review funded?
**Obesity**	13/04/20	06/04/20	-	-	-	-	NIHR Applied Research and Care
**Obesity**	29/04/20	01/05/20	NS	None	None	No	University of Indonesia
**Obesity**	13/05/20	15/05/20	NS	No	None	None	Programme Széchenyi 20
**Obesity**	10/11/20	12/11/20	SD	No	No	Perhaps update of review potentially needed	None
**Pregnancy**	04/04/20	23/06/20	-	-	-	-	Partly funded by WHO
**Pregnancy**	13/04/20	17/04/20	-	-	-	-	None
**Pregnancy**	24/04/20	24/04/20	NS*	No	None	No	None
**Skin Manifestations**	23/06/20	25/06/20	-	-	-	-	None

Research area	Date submitted	Date registered	Answer to screening question?	Have authors acknowledged similar reviews?	Authors reason for duplication?	Is there reason for duplication from our perspective?	Is the review funded?
**Skin Manifestations**	28/06/20	29/06/20	SD	Yes	Yes (none will be published before completion)	No	None
**Treatment: ACE Inhibitors and ARBs**	07/06/20	19/06/20	-	-	-	-	Major Project of National Science and Technology on New Drug etc.
**Treatment: ACE Inhibitors and ARBs**	03/07/20	08/07/20	SD	No	None	No	None
**Treatment: Metformin**	21/08/20	25/08/20	-	-	-	-	ARC-EM
**Treatment: Metformin**	12/09/20	15/09/20	RN	None	None	No	National Natural Science China
**Breastfeeding**	06/04/20	21/04/20	SD	References CRD420173886	Yes. But review is sufficiently so no duplication occurred.	Yes	None
**Breastfeeding**	11/04/20	20/04/20	N/A	-	-	-	None
**Breastfeeding**	17/07/20	20/07/20	SD	Not explicitly	No	No	None

**Research area**	**Date submitted**	**Date registered**	**Answer to screening question?**	**Have authors acknowledged similar reviews?**	**Authors reason for duplication?**	**Is there reason for duplication from our perspective?**	**Is the review funded?**
**Breastfeeding**		10/10/20	NS	No	No	No comparator.	None
**Maternal to Fetal Transmission**	16/03/20	24/03/20	NS	-	-	-	N/A
**Maternal to Fetal Transmission**	22/06/20	03/07/20	SD	No	No	No	None
**Surgery**	02/04/20	22/04/20	NS	-	-	-	None
**Surgery**	25/04/20	28/04/20	NS	No	N/A	No	None
**Viral Shedding**	24/04/20	29/04/20	NS	-	-	-	None
**Viral Shedding**	22/06/20	22/06/20	NS*	Yes, acknowledge CRD420181914	Yes, previous registration deemed poor quality	Potentially- poor reporting in previous registration. Authors have given reason for duplication.	None
**Weather**	28/03/20	07/04/20	NS	-	-	-	None

Research area	Date submitted	Date registered	Answer to screening question?	Have authors acknowledged similar reviews?	Authors reason for duplication?	Is there reason for duplication from our perspective?	Is the review funded?
**Weather**	01/04/20	08/04/20	NS	-	-	-	Federal University of Pará, Brazil.
**Weather**	27/04/20	30/04/20	NS	No	No	Yes, looking at seasons and times of year rather than climate conditions.	None
**Weather**	03/07/20	03/07/20	SD	No	No	Almost identical. No real reason for duplication.	None
**Ace inhibitors**	05/04/20	14/04/20	NS	-	-	-	Self funded
**Ace inhibitors**	13/04/20	17/04/20	NS*	-	-	-	None
**Ace inhibitors**	03/05/20	05/05/20	NS	No	NO	NO	None

Research area	Date submitted	Date registered	Answer to screening question?	Have authors acknowledged similar reviews?	Authors reason for duplication?	Is there reason for duplication from our perspective?	Is the review funded?
**Ace inhibitors**	04-Mayx-20	04/05/20	NS	No	NO	NO	China Postdoctoral Science Foundation (2019M652593) and Henan Postdoctoral Science Foundation (1902006)
**Ace inhibitors**	08/05/20	11/05/20	SD	Partially	NO	NO	None
**Ace inhibitors**	13/05/20	14/05/20	NS	No	NO	NO	This study was supported by National Natural Science Foundation of China, 81970203; National Natural Science Foundation of China, 81570212; National Natural Science Foundation of China, 31800976 Chongqing Science and Health Joint Medical Research Project, 2018QNXM024
**Ace inhibitors**	27/07/20	28/07/20	NS	No	NO	NO	Qingsong Qin is sponsored by Department of education of Guangdong province, China

**Research area**	**Date submitted**	**Date registered**	**Answer to screening question?**	**Have authors acknowledged similar reviews?**	**Authors reason for duplication?**	**Is there reason for duplication from our perspective?**	**Is the review funded?**
**Ace inhibitors**	03/08/20	04/08/20	SD	Partially	NO	NO	Disciplines Construction Project of PUMC (Grant number: 20192102). Chinese Academy of Medical Sciences, CAMS Innovation Fund for Medical Sciences (Grant number: 2018-I2M-1-002)
**All treatment living**	20/04/20	22/04/20	SD	-	-	-	None
**All treatment living**	27/04/20	28/04/20	SD	Partially	NO	No	This review received some funding from the Agence Nationale de la Recherche (ANR) and from the WHO
**All treatment MA**	07-Feb-20	31/03/20	NS	-	-	-	NA

Research area	Date submitted	Date registered	Answer to screening question?	Have authors acknowledged similar reviews?	Authors reason for duplication?	Is there reason for duplication from our perspective?	Is the review funded?
**All treatment MA**	09/03/20	20/03/20	NS	No	No	No	This study will be funded by Research Funds of Shanghai Health and Family Planning commission (20184Y0022, 20194Y0007), Cultivation fund of clinical research of Renji hospital (PY2018-III-06), Clinical Pharmacy Innovation Research Institute of Shanghai Jiao Tong University School of Medicine (CXYJY2019ZD001, CXYJY2019QN004).
**All treatment MA**	22/03/20	24/03/20	NS	No	No	No	Shubham Misra is a recipient of DST-INSPIRE PhD Fellowship from Department of Science & Technology, Government of India
**All treatment MA**	07/04/20	17/04/20	NS	No	No	No	https://covid19.kacst.edu.sa/grants/

Research area	Date submitted	Date registered	Answer to screening question?	Have authors acknowledged similar reviews?	Authors reason for duplication?	Is there reason for duplication from our perspective?	Is the review funded?
**All treatment MA**	20/04/20	22/04/20	SD	Partially	No	No	None
**All treatment MA**	05/05/20	06/05/20	NS	No	No	No	None
**All treatment MA**	13/10/20	16/10/20	NS	No	No	No	This work was funded by the Guidance Plan for Social Development of Taizhou Municipal Science and Technology (ssf20160141).
**All treatment MA**	14/11/20	07/12/20	NS	No	No	No	None
**All treatment MA**	05/01/21	08/01/21	Not in system	No	No	No	None
**All treatment NMA**	07/04/20	15/04/20	NS	NO	Yes		
**All treatment NMA**	12/04/20	16/04/20	SD	Partially	NA		
**All treatment NMA**	18/04/20	22/04/20	SD	Partially	No	No	The research project will be assisted with the by Griffith University

Research area	Date submitted	Date registered	Answer to screening question?	Have authors acknowledged similar reviews?	Authors reason for duplication?	Is there reason for duplication from our perspective?	Is the review funded?
							Postgraduate Research Scholarship
**All treatment NMA**	19/04/20	20/04/20	NS	NO	No	No	None
**All treatment NMA**	23/04/20	23/04/20	NS	NO	No	No	Supported by the Programme of Introducing Talents of Discipline to Universities, China, the 111 Project, D18011)
**All treatment NMA**	27/04/20	27/04/20	SD	Partially	No	No	None
**All treatment NMA**	15/05/20	19/05/20	NS	NO	No	No	None
**All treatment NMA**	18/06/20	24/06/20	NS	NO	No	No	None
**All treatment NMA**	18/10/20	26/10/20	NS	NO	No	No	None
**All treatment umbrella**	13/05/20	20/05/20	NS	NA	NA	NA	NA

Research area	Date submitted	Date registered	Answer to screening question?	Have authors acknowledged similar reviews?	Authors reason for duplication?	Is there reason for duplication from our perspective?	Is the review funded?
**All treatment umbrella**	07/09/20	07/09/20	NS	No	No	No	Indian Council of Medical Research
**All treatment umbrella**	04/10/20	05/10/20	NS	No	No	No	None
**Anticoagulant**	02/06/20	02/06/20	NS	-	-	-	
**Anticoagulant**	02/07/20	03/07/20	SD	Partially	No	No	None
**Anticoagulant**	14/10/20	15/10/20	NS	No	No	No	None
**Anticoagulant**	07/01/21	11/01/21	not in list	No	No	No	None
**Blood plasma**	12/03/20	24/03/20	NS	-	-	-	None
**Blood plasma**	28/03/20	08/04/20	SD	Partially		No reasons why sufficiently different.	Funded by the UK Forum
**Blood plasma**	02/04/20	02/04/20	NS	No	No	No	None

Research area	Date submitted	Date registered	Answer to screening question?	Have authors acknowledged similar reviews?	Authors reason for duplication?	Is there reason for duplication from our perspective?	Is the review funded?
**Blood plasma**	06/04/20	15/04/20	SD	Partially	No	No reasons why sufficiently different.	None
**Blood plasma**	10/04/20	15/04/20	NS	No	No	No	Education Foundation of Chengdu University of Traditional Chinese Medic
**Blood plasma**	17/06/20	19/06/20	SD	Partially	No	No	None
**Blood plasma**	05/07/20	06/07/20	SD	Partially	No	No reasons why sufficently different.	None
**Blood plasma**	16/07/20	16/07/20	NS	No	No	No	None
**Blood plasma**	12/08/20	14/08/20	SD	Partially	No	No reasons why sufficently different.	None
**Blood plasma**	09/12/20	22/12/20	not in list	No	No	No	None
**Blood plasma**	16/12/20	17/12/20	not in list		No	No	None

**Research area**	**Date submitted**	**Date registered**	**Answer to screening question?**	**Have authors acknowledged similar reviews?**	**Authors reason for duplication?**	**Is there reason for duplication from our perspective?**	**Is the review funded?**
**Blood plasma**	21/12/20	22/12/20	not in list	No	Potentially severe patient pop.	No	None
**Colchicine**	17/09/20	21/09/20					None
**Colchicine**	11/01/21	12/01/21	not in list	No	No	No	None
**Corticosteroids**	03/03/20	20/04/20	NS	-	-	-	None
**Corticosteroids**	20/04/20	21/04/20	NS	-	-	-	None
**Corticosteroids**	28/04/20	30/04/20	NS	No	No	No	None
**Corticosteroids**	06/05/20	06/05/20	SD	Partially	No	No	This work was supported by National Key Research and Development Project (20YFC2005700), Key Realm R&D Program of Guangdong Province (2019B030335001), and Medical Scientific Research

Research area	Date submitted	Date registered	Answer to screening question?	Have authors acknowledged similar reviews?	Authors reason for duplication?	Is there reason for duplication from our perspective?	Is the review funded?
							Foundation of Guangdong Province of China (A2019407)
**Corticosteroids**	07/07/20	07/07/20	SD	Partially	No	No	None
**Corticosteroids**	23/09/20	23/09/20	SD	Partially	No	No	None
**Corticosteroids**	07/12/20	08/12/20	not in list	No	No	No	None
**Corticosteroids**	21/12/20	22/12/20	not in list	No	No	No	NaFoUniMedCovid19“ (FKZ: 01KX21) „CEO-Sys“
**Corticosteroids**	05/01/21	12/01/21	not in list	No	No	No	None
**ECMO**	02/04/20	14/04/20		-	-	-	None

Research area	Date submitted	Date registered	Answer to screening question?	Have authors acknowledged similar reviews?	Authors reason for duplication?	Is there reason for duplication from our perspective?	Is the review funded?
**ECMO**	07/04/20	14/04/20		-	-	-	None
**ECMO**	16/04/20	17/04/20	NS	No	No	No	None
**ECMO**	03/05/20	04/05/20	SD	Partially	No	No	None
**ECMO**	15/06/20	18/06/20	NS	No	No	No	None
**ECMO**	19/06/20	22/06/20	NS	No	No	No	None
**ECMO**	17/09/20	21/09/20	NS	No	No	No	None
**ECMO**	22/11/20	23/11/20	NS	No	No	No	This work was supported by the research Grant 20YFC0841300 from Ministry of Science and Technology of the People's Republic of China.
**ECMO**	21/12/20	22/12/20	NS	No	No	No	None

Research area	Date submitted	Date registered	Answer to screening question?	Have authors acknowledged similar reviews?	Authors reason for duplication?	Is there reason for duplication from our perspective?	Is the review funded?
**ECMO**	04/01/21	07/01/21	NS	No	No	No	None
**Hydroxychloroquine**	22-Feb-20	17/03/20		-	-	-	None
**Hydroxychloroquine**	29/03/20	16/04/20	NS	No	No	No	Major Projects of Science and Technology Innovation 2025 in Ningbo, China
**Hydroxychloroquine**	04/04/20	06/04/20	SD	Partially	No	No	NIHR Applied Research Collaboration East Midlands (ARC EM), UK
**Hydroxychloroquine**	06/04/20	07/04/20	SD	Partially	No	No	None
**Hydroxychloroquine**	06/04/20	09/04/20	NS	No	No	No	None
**Hydroxychloroquine**	07/04/20	17/04/20	NS	No	No	No	None
**Hydroxychloroquine**	10/04/20	21/04/20	SD	Partially	No	No	None
**Hydroxychloroquine**	14/04/20	17/04/20	SD	Partially	No	No	None

Research area	Date submitted	Date registered	Answer to screening question?	Have authors acknowledged similar reviews?	Authors reason for duplication?	Is there reason for duplication from our perspective?	Is the review funded?
**Hydroxychloroquine**	20/04/20	21/04/20	SD	Partially	No	No	None
**Hydroxychloroquine**	27/04/20	28/04/20	NS	No	No	No	None
**Hydroxychloroquine**	22/05/20	22/05/20	SD	Partially	No	No	None
**Hydroxychloroquine**	10/08/20	12/08/20	NS	No	No	No	None
**Hydroxychloroquine**	19/08/20	19/08/20	NS	No	No	No	None
**Hydroxychloroquine**	12/09/20	17/09/20	NS	No	No	No	None
**Hydroxychloroquine**	12/09/20	15/09/20	SD	Partially	No	No	None
**Interferon**	26/03/20	26/03/20	NA	-	-	-	None
**Interferon**		07/04/20	NA	-	-	-	None
**Interferon**	17/11/20	20/11/20	SD	Partially	No	No	None

**Research area**	**Date submitted**	**Date registered**	**Answer to screening question?**	**Have authors acknowledged similar reviews?**	**Authors reason for duplication?**	**Is there reason for duplication from our perspective?**	**Is the review funded?**
**Interferon**	21/11/20	03/12/20	NS	No	No	No	None
**Ivermectin**	08/07/20	09/07/20		-	-	-	-
**Ivermectin**	20/10/20	09/11/20	NS	no	No	No	None
**Ivermectin**	24/12/20	07/01/21	NS	no	No	No	The Institute for Health Technology Assessment (IETS),
**Ivermectin**	11/01/21	12/01/21	NS	no	No	No	None
**JAK inhibitors**	07/06/20	09/06/20		-	-	-	NA
**JAK inhibitors**	04/01/21	12/01/21	SD	partially	no	no	None
**lopinavir**	06/04/20	15/04/20	NA	NA	NA	NA	NA
**lopinavir**	09/04/20	15/04/20	NA	NA	NA	NA	NA

Research area	Date submitted	Date registered	Answer to screening question?	Have authors acknowledged similar reviews?	Authors reason for duplication?	Is there reason for duplication from our perspective?	Is the review funded?
**lopinavir**	12/04/20	16/04/20	NA	NA	NA	NA	NA
**lopinavir**	17/04/20	21/04/20	NS	no	no	no	None
**lopinavir**	24/04/20	24/04/20	NS	no	no	no	None
**lopinavir**	13/01/21	15/01/21	SD	partially	no	no	This study is carried out at the request of the Ministry of Health and Social Protection and the Disaster Risk Management Fund. Contract No. 9677-MECOVID19-1009-20.
**Prone position**	24/06/20	24/06/20	NA	-	-	-	-
**Prone position**	28/08/20	01/09/20	NS	no	no	no	None
**Prone position**	11/09/20	14/09/20	NS	no	no	no	None
**Prone position**	19/10/20	22/10/20	NS	no	no	no	None

Research area	Date submitted	Date registered	Answer to screening question?	Have authors acknowledged similar reviews?	Authors reason for duplication?	Is there reason for duplication from our perspective?	Is the review funded?
**Prone position**	16/11/20	20/11/20	RN	yes	no	no	None
**Prone position**	10/12/20	11/12/20	NS	no	no	no	None
**Prone position**	12/12/20	14/12/20	NS	no	no	no	None
**RAAS**	07/05/20	11/05/20		-	-	-	None
**RAAS**	14/06/20	17/06/20	NS	no	no	no	None
**RAAS**	15/09/20	16/09/20	RN	yes	no	no	None
**Remdisivir**	08-Feb-20	23/03/20	NS	-	-	-	National Natural Science Foundation of China
**Remdisivir**	02/04/20	14/04/20	NS	No	No	No	CDT-Africa, College of Health Sciences, Addis Ababa University
**Remdisivir**	21/04/20	23/04/20	NS	No	No	No	None
**Remdisivir**	30/04/20	01/05/20	SD	No	No	No	None

Research area	Date submitted	Date registered	Answer to screening question?	Have authors acknowledged similar reviews?	Authors reason for duplication?	Is there reason for duplication from our perspective?	Is the review funded?
**Remdisivir**	26/05/20	26/05/20	NS	No	No	No	Tongji Hospital, Tongji Medical College
**Remdisivir**	20/09/20	21/09/20	NS	No	No	No	None
**Remdisivir**	12/10/20	15/10/20	NS	No	No	No	Zhejiang Medicine and Health Science and Technology Project; Natural Science Foundation of Zhejiang Province
**Tocilizumab**	22/03/20	24/03/20	NS	-	-	-	None
**Tocilizumab**	18/06/20	19/06/20	NS		No	No	None
**Tocilizumab**	21/06/20	22/06/20	SD		No	No	None
**Tocilizumab**	26/06/20	01/07/20	NS		No	No	Nicola Luigi Bragazzi and Jianhong Wu are partially supported by the Canadian Institute of Health Research

Research area	Date submitted	Date registered	Answer to screening question?	Have authors acknowledged similar reviews?	Authors reason for duplication?	Is there reason for duplication from our perspective?	Is the review funded?
							(CIHR) 2019 Novel Coronavirus (COVID-19) rapid research program
**Tocilizumab**	14/08/20	17/08/20	NS		No	No	None
**Tocilizumab**	06/11/20	10/11/20	NS	no	No	No	None
**Tocilizumab**	11/12/20	18/12/20	NS	no	No	No	1.3.5 project for disciplines of excellence, West China Hospital, Sichuan University
**Tocilizumab**	16/12/20	17/12/20	SD	Partially	No	No	None

* Registrant changed their answer to the screening question. Grey cells represent 'duplicate' records, white cells represent records registered first.
Abbreviations: NS, Not Similar; RN, Repetition Needed; SD, Sufficiently Different.

Appendix E

Duplicate Registrations Characteristics

Table 2: Number of 'duplicate' records registered in each COVID-19 related topic area.

Topic	N duplicates
**Epidemiology**	
**Health Condition and Impact on Outcome**	
Cancer	1
Cardiovascular disease	1
Diabetes	2
General Risk Factors	8
Obesity	3
Comorbidities	6
**Healthcare Workers**	
COVID-19 in healthcare workers	1
Mental Health in Healthcare Workers	1
**Clinical presentation of COVID-19**	
Echocardiographic Findings	1
Manifestations (Dermatological)	1
Manifestations (Taste and Smell)	1
Manifestations (Neurological)	1
Long-COVID	1
**Transmission of COVID-19**	
Facemasks	1
Pregnancy and mother-baby transmission	1
**Treatment of COVID-19**	
ACE-inhibitors	1
Metformin	1
**Total number of duplicates**	**31**
**Treatments**	
**All treatments**	
All treatment living	1
All treatment MA	7
All treatment umbrella	2
All treatment NMA	8
**Pharmacological interventions**	
ACE-inhibitors	6
Anticoagulants	1
Corticosteroids	4
Hydroxychloroquine	14
Interferon	2

Ivermectin	3
JAK inhibitors	1
**Topic**	**N duplicates**
**Pharmacological interventions**	
lopinavir	3
RAAS	2
Remdisivir	6
Tocilizumab	7
**Non-pharmacological interventions**	
Blood plasma	8
ECMO	5
Prone position	6
**Total number of duplicates**	**86**
**Transmission**	
Mother-baby transmission	2
Transmission during Surgery	1
Weather	1
**Total number of duplicates**	**4**
