# Supplementary material for: Extent and nature of duplication in PROSPERO using COVID-19-related registrations: a retrospective investigation and survey
Source: BMJ Open. 2022 Dec 1;12(12):e061862. doi: 10.1136/bmjopen-2022-061862 (PMC9716408; doi:10.1136/bmjopen-2022-061862)
Supplement: Author's manuscript [file bmjopen-2022-061862.draft_revisions.pdf]

BMJ Open

BMJ Open is committed to open peer review. As part of this commitment we make the peer review history of every article we publish publicly available.

When an article is published we post the peer reviewers' comments and the authors' responses online. We also post the versions of the paper that were used during peer review. These are the versions that the peer review comments apply to.

The versions of the paper that follow are the versions that were submitted during the peer review process. They are not the versions of record or the final published versions. They should not be cited or distributed as the published version of this manuscript.

BMJ Open is an open access journal and the full, final, typeset and author-corrected version of record of the manuscript is available on our site with no access controls, subscription charges or pay-per-view fees (http://bmjopen.bmj.com).

If you have any questions on BMJ Open's open peer review process please email info.bmjopen@bmj.com

BMJ Open

The extent and nature of duplication in PROSPERO: an investigation using COVID-19 related registrations

Journal:	*BMJ Open*
Manuscript ID	bmjopen-2022-061862
Article Type:	Original research
Date Submitted by the Author:	15-Feb-2022
Complete List of Authors:	Beresford, Lucy; University of York, Centre for Review and Dissemination<br>Walker, Ruth; University of York Centre for Reviews and Dissemination<br>Stewart, Lesley; University of York Centre for Reviews and Dissemination
Keywords:	COVID-19, STATISTICS & RESEARCH METHODS, QUALITATIVE RESEARCH

SCHOLARONE™
Manuscripts

I, the Submitting Author has the right to grant and does grant on behalf of all authors of the Work (as defined in the below author licence), an exclusive licence and/or a non-exclusive licence for contributions from authors who are: i) UK Crown employees; ii) where BMJ has agreed a CC-BY licence shall apply, and/or iii) in accordance with the terms applicable for US Federal Government officers or employees acting as part of their official duties; on a worldwide, perpetual, irrevocable, royalty-free basis to BMJ Publishing Group Ltd ("BMJ") its licensees and where the relevant Journal is co-owned by BMJ to the co-owners of the Journal, to publish the Work in this journal and any other BMJ products and to exploit all rights, as set out in our licence.

The Submitting Author accepts and understands that any supply made under these terms is made by BMJ to the Submitting Author unless you are acting as an employee on behalf of your employer or a postgraduate student of an affiliated institution which is paying any applicable article publishing charge ("APC") for Open Access articles. Where the Submitting Author wishes to make the Work available on an Open Access basis (and intends to pay the relevant APC), the terms of reuse of such Open Access shall be governed by a Creative Commons licence – details of these licences and which Creative Commons licence will apply to this Work are set out in our licence referred to above.

Other than as permitted in any relevant BMJ Author's Self Archiving Policies, I confirm this Work has not been accepted for publication elsewhere, is not being considered for publication elsewhere and does not duplicate material already published. I confirm all authors consent to publication of this Work and authorise the granting of this licence.

For peer review only

Manuscript Title:

The extent and nature of duplication in PROSPERO: an investigation using COVID-19 related registrations

Authors:

Miss Lucy Beresford, Research Fellow in Evidence Synthesis[1]
lucy.beresford@york.ac.uk*

Ruth Walker, Research Fellow in Evidence Synthesis[1]
ruth.walker@york.ac.uk*

Professor Lesley Stewart, Professor of Evidence Synthesis[1]
lesley.stewart@york.ac.uk

[1] Centre for Reviews and Dissemination, University of York
* These authors contributed equally to the work

Contact information for corresponding author

Ruth Walker, Research Fellow in Evidence Synthesis
Centre for Reviews and Dissemination, University of York
ruth.walker@york.ac.uk

For peer review only

Abstract

Objectives

This project explored the extent of and reasons for duplication of COVID-19 related systematic review registrations in PROSPERO (international prospective register of systematic reviews) during the pandemic.

Methods

We focused on four COVID-19 research areas: epidemiology, rehabilitation, transmission and treatments. Records were compared using the PICOS (Population, Intervention, Comparator, Study Design) elements of the PROSPERO registration form. Registrations that had similar or identical PICOS were retained for further evaluation as 'duplicates'.

Those registering 'duplicates' were invited to complete a short questionnaire, asking whether they searched PROSPERO prior to registration, identified the similar reviews, and if so, why they continued with their review.

Results

1054 COVID-19 reviews were registered between March 2020 and January 2021, of which 138 were identified as having been submitted when at least one similar protocol was already registered in PROSPERO. Duplication was greatest in reviews of COVID-19 treatments; for example, there were 14 similar reviews evaluating the efficacy of hydroxychloroquine.

We contacted the authors of 'duplicate' records and received 41 responses. Most respondents said that they identified similar reviews when they searched PROSPERO prior to registration. The main reasons they gave for 'duplication' were differences in PICOS or planned analyses, poor quality of previous registrations, and the need to update the evidence.

Conclusions

This research highlights that registration of similar and duplicate systematic reviews related to COVID-19 in PROSPERO occurred frequently. Increased awareness of research waste is required, and initial checking for similar reviews should be embedded within good review practice.

For peer review only - http://bmjopen.bmj.com/site/about/guidelines.xhtml

Introduction

During the COVID-19 pandemic, a significant volume of medical research has been conducted to better understand the virus, its biology and associated disease.[1] Large numbers of systematic reviews have synthesised the emerging evidence.[2] It is best practice to register systematic reviews at the protocol stage to help avoid unplanned duplication and to enable comparison between methods reported in the final publication and that which was planned in the protocol.[3] This can be done in registries such as PROSPERO, an international prospective register of systematic review protocols, funded by the National Institute of Health Research in the UK. PROSPERO was launched in 2011 with the intention of increasing transparency, limiting unintended duplication, and reducing the risk of bias in systematic reviews.[4-6]

In March 2020, PROSPERO prioritised registration of review protocols related to COVID-19. Our intention was to support the global pandemic research endeavour by making information about planned and ongoing reviews public as quickly as possible and providing researchers and policy makers with an overview of the evidence synthesis pipeline. This would also help reduce unintended duplication, by enabling authors to check whether similar reviews already existed before embarking on a new one, based on up-to-date information (COVID-19 registrations are published within 24 hours of submission Monday- Friday). Submissions are registered in PROSPERO through the process described in Box 1.

Box 1.

- Registration in PROSPERO involves the submission and publication of key information about the design and conduct of a systematic review
- Authors should register their systematic review once they have designed their review protocol but before they begin data extraction
- Prior to submission authors answer a series of 'triage questions' to check whether their review is eligible for inclusion.
- Previously, applications were checked by the PROSPERO team to ensure that they were in scope and that the required data had been provided. Since March 2020, to enable rapid processing of COVID-19 registrations, some applications have been published automatically following simple automated checks. No quality assessment or peer review is involved. Records are published on an open access electronic database.
- Registration information can be amended by authors should plans change. These changes are published, and a date stamped audit trail of previous versions made available in the public record.
- Registration records are permanent, and authors are asked to provide links to subsequent reports and publications.
- PROSPERO assigns each registered review a unique registration number. This number should be cited in publications.
- Peer reviewers and readers can compare what was reported for the review with what was intended in the review protocol.

For peer review only - http://bmjopen.bmj.com/site/about/guidelines.xhtml

To aid identification of reviews on topics of key relevance to pandemic research, a single click search by broad COVID-19 research categories was implemented on the PROSPERO home page. The research categories have been regularly updated as new topics emerge. PROSPERO encourages authors not to duplicate existing systematic reviews but recognises that duplication may be intended with good reason (e.g., the existing review is out of date or used suboptimal methods). As such, when registering, users are asked to indicate whether their review is (i) not similar to an existing review, (ii) sufficiently different, or (iii) similar to an existing review, but repetition is needed, as part of a series of eligibility questions before completing the registration form.

Despite encouragement to check PROSPERO for similar systematic reviews and to not duplicate without good reason, it quickly became apparent that multiple systematic reviews related to COVID-19 that addressed the same research question were being registered. Unnecessary duplication of research is wasteful and creates additional work for healthcare providers who need to make sense of evidence in a rapidly evolving field. Duplication can also cause confusion, if findings between reviews are inconsistent.[1] We aimed to explore the extent of duplication within PROSPERO registration records related to COVID-19, including whether the extent differed between rapid, full and living systematic reviews and/or particular areas of COVID-19 related research. We also aimed to explore the nature of duplication, including whether this was done unknowingly, covertly or for good reason by authors who were open and transparent about replication. Finally, we aimed to better understand the reasons for repetition from the authors' perspective and explore who funded duplicate reviews.

Methods

Searches

PROSPERO was searched for registrations relating to COVID-19 from March 2020 to January 2021. Four COVID-19 research categories were selected for further study: epidemiology, transmission, treatment, and rehabilitation. For practical reasons, the records were downloaded for reviews of transmission, rehabilitation and epidemiology in November 2020, and treatment in January 2021.

Identification of duplicate records

Within each topic area, records were each assigned a keyword based on the review title. Those with the same keyword were compared, firstly using the title of the registration record and then for those with similar titles, using the Population, Intervention/Exposure, Comparator, Outcome and Study

For peer review only - http://bmjopen.bmj.com/site/about/guidelines.xhtml

design (PICOS) criteria recorded in the PROSPERO registration form. Those addressing the same research question, based on the PICOS criteria were retained. To ensure that decisions were being made consistently, this was conducted in duplicate by two reviewers for a subset of records (the rehabilitation and transmission reviews, and a random sample of epidemiology registrations (100 from 445 records)). Discrepancies in decisions were discussed between reviewers to reach a consensus. The remaining records were screened singularly.

For reviews addressing the same research question, the date of first submission to PROSPERO and the date of publication were obtained. Registrations submitted after the publication of a record with a similar or identical PICOS, were deemed 'duplicates' and retained. Reviews that have been submitted to PROSPERO but not yet registered/published on the public website (for example if the record was returned to authors for additional information or clarification) are not visible on the PROSPERO COVID-19 search. Therefore, if a record was *submitted* before another record on the same topic and *published* after it, this was not counted as duplication because the authors would not have had knowledge of the other review at the point they submitted their registration form.

Data extraction

For duplicate reviews, data on whether authors acknowledged similar existing registrations in PROSPERO when answering the screening questions prior to registration were recorded. From the public registration forms, data concerning the nature of duplication were extracted (see Appendix, Table 1 for the Data Extraction template). This included whether the authors provided reasons for conducting a new review, whether they had an external funding source and whether reviews were rapid, full, or living systematic reviews (this information was used to assess whether full systematic reviews were conducted after rapid reviews for particular research topics, and/or whether rapid or full systematic reviews were being registered after living systematic reviews).

Surveys

To further understand the reasons why people duplicated existing reviews, authors of duplicate registrations were contacted by email and invited to complete a short questionnaire. Authors were asked whether they searched PROSPERO prior to registration, and whether they identified any similar reviews. Authors were also asked why they believed their review to be sufficiently different from an existing review, or why they felt repetition was needed if they had identified a similar review. Reminders to complete the questionnaire were sent one week before it closed. A copy of the questionnaire is given in the Appendix.

For peer review only - http://bmjopen.bmj.com/site/about/guidelines.xhtml

Patient Public Involvement

Patients or the public were not involved in the design, conduct or reporting, or dissemination plans of our research. Our findings from this research were discussed with the COVID Evidence Network to support Decision making (COVID-END) community.

Results

Search results

From 01 March 2020 to 31st of January 2022, PROSPERO registered 5,959 protocols related to COVID-19. The monthly number of registrations published on PROSPERO peaked in April 2020 with 588 protocols (Figure 1). Overall, 1054 registrations related to the four COVID-19 topic areas of interest (until January 2021).

Descriptive characteristics

574 records were found to have a similar review title and had their full PROSPERO record examined. 195 of these records had a similar or identical PICO(S) (57 of which were the first instance of reviews addressing particular research questions). The remaining 138 duplicate records had been submitted when at least one protocol with similar or identical PICOS had already been published in PROSPERO. Figure 2 presents the number of records at each stage of the screening process.

There were 33 (7.4% of those registered) duplicate reviews for epidemiological topics, 98 (20%) for treatments and seven (7.5%) relating to the transmission of COVID-19. No duplicate reviews were identified in the rehabilitation topic area. Figure 3 shows the number of duplicated reviews registered by month between March 2020 and January 2021 in each research area.

The number of duplicate reviews on specific questions ranged from one to 14. Multiple duplicate registrations were most common in reviews evaluating COVID-19 treatments, including reviews evaluating the efficacy of drugs such as hydroxychloroquine (14 duplicate reviews), tocilizumab (7), remdesivir (6), the use of blood plasma (7) and prone position (6). The majority of epidemiological reviews only had a single duplicate review, except for those evaluating the impact of comorbidities (six duplicate reviews) and reviews of general risk factors in patients with COVID-19 (8). The number of duplicates per research question are given in the Appendix, Table 2.

For peer review only - http://bmjopen.bmj.com/site/about/guidelines.xhtml

Screening questions

When answering the PROSPERO screening questions prior to registering their protocol, authors of 85 of the 138 records considered as duplicate reviews did not acknowledge existing similar reviews in PROSPERO and selected ‘Not Similar’ on the screening question. 47 indicated that their review was sufficiently different to those already registered and five authors indicated that repetition of existing reviews was needed.

Review type

Table 1 shows the number of registrations published on PROSPERO by research area and review type. We identified 138 duplicate full systematic reviews, only two of which were replacing rapid reviews that addressed the same research question.

Table 1. Number of Registered and Duplicate Reviews in four COVID-19 topic areas by type of systematic review.

Review Type	Number of Registered Reviews				Number of Duplicate Reviews			
	Epidemiology	Rehabilitation	Treatment	Transmission	Epidemiology	Rehabilitation	Treatment	Transmission
**Rapid**	16[a]	2	21[b]	7	1	0	2	0
**Living**	12	3	27	7	1	0	6	1
**Full**	417	24	439	79	31	0	90	6

[a]Two reviews were rapid, living systematic reviews and are counted in the ‘living’ row. [b]One review was a rapid, living systematic review and is counted in the ‘living’ row.

It was often difficult to differentiate between rapid and full systematic reviews as many of the registration forms were poorly completed. Some reviews also indicated they would complete within 4-6 weeks but did not identify as being a rapid review or detail abbreviated or rapid review methods, such as use of text-mining during the screening process.

Twenty-three full systematic reviews and one living systematic review were found to be registered after living systematic reviews addressing the same research question.

Reasons for duplication

Provided in registrations

For each duplicate record, we explored whether the authors had acknowledged previous registrations in their PROSPERO record, and if so, gave reasons for duplication. Thirty-three (24%) authors of duplicate records did so. Only 4 of these authors (12%) provided a reason for replication

For peer review only - http://bmjopen.bmj.com/site/about/guidelines.xhtml

within their record despite being advised during the registration process that the reasons for duplication should be made clear.

Provided in response to survey

We received 41 unique responses to the survey investigating reasons for duplication, from 12 countries (Figure 4). Nineteen responses (22% of those contacted) were from authors who during PROSPERO screening indicated that their review was 'not similar' to existing registered reviews, 20 responses, (42% of those contacted) indicated their review was 'sufficiently different' and two responses (40% of those contacted) indicated 'repetition was needed'.

Of the respondents that provided their registration number in the survey, 68% had registered reviews relating to treatments for COVID-19. Overall, 90% of respondents had conducted a systematic review previously, and 92.5% intended to publish the results of their review. Most respondents had received no specific funding for their systematic review (87.5%).

11/19 respondents who indicated their review was 'not similar' to existing registered reviews, responded in the survey that they searched PROSPERO and did not identify any reviews on the same topic area. When asked to provide search terms used to do this, some were so broad that a large number of results would have been returned (such as single words like 'COVID-19'). Other respondents used multiple search terms; when we repeated these searches, all identified the duplicate records related to their review. 8/19 respondents said they searched PROSPERO and found some reviews on the same topics, but decided their review was different to the existing reviews found by their search.

19/20 respondents who indicated in the screening questions that their reviews were sufficiently different to other registered reviews, responded that their searches identified some or all the reviews that we identified as being similar to their own. However, no respondent provided justification to why they believed their review was significantly different to previous registrations in their PROSPERO registration.

Of the two respondents who indicated that repetition was needed, neither provided their justification for repeating previous systematic reviews.

The reasons given for conducting similar reviews are summarised in Table 2.

Table 2. *Summary of reasons for duplications from respondents of the questionnaire*

**Response to Screening Question**	**Responses in Survey**

For peer review only - http://bmjopen.bmj.com/site/about/guidelines.xhtml

**Not Similar**	• Different objectives or planned analyses<br>• Poor quality of previous systematic reviews<br>• Duplication was needed owing to the rapid rate that new studies on COVID-19 were being published
**Sufficiently Different**	• Differences in PICOS, study design or planned analyses<br>• Differences in databases searched<br>• Poor quality of previous systematic reviews<br>• Duplication was needed owing to the rapid rate that new studies on COVID-19 were being published
**Repetition Needed**	• No systematic review was published or completed at the time of registration<br>• Repetition was needed to confirm/refute previous systematic review conclusions*

For peer review only

Discussion

To synthesise the considerable amount of primary research conducted during the COVID-19 pandemic [7], a substantial number of systematic reviews have been produced, many of which have been prospectively registered on PROSPERO. There is growing concern that these include duplicate systematic reviews that have replicated without good reason.7 Unintended or unnecessary duplication of research wastes research funding, time, and effort and creates extra work for policy makers and healthcare providers who need to determine what novel information, if any, new reviews provide.[2 5 8]

We aimed to assess the extent and nature of duplication within PROSPERO registration records related to COVID-19, within four research areas: epidemiology, rehabilitation, transmission, and treatment. Duplication was found to be extensive (accounting for 13% of records across these research areas). Researchers may have instead made a more valuable contribution to knowledge by considering an alternative review topic (as advised on the PROSPERO homepage).

The nature of this duplication was first examined by assessing whether duplication was a result of rapid reviews (a form of evidence synthesis in which some systematic review methods are condensed or omitted to produce results in a timely manner) being replaced by full systematic reviews.[9] As rapid reviews provide a useful overview of the available research, but may stop short of providing a full or detailed synthesis, a later full systematic review may be justified. However, only two examples of this were identified in our sample. We also identified rapid and full systematic reviews registered after living systematic reviews on the same topic. For example, following the registration of a living systematic review and network meta-analysis on all interventions for COVID-19, an additional seven duplicate standard systematic reviews were registered. This is particularly

wasteful as living reviews update regularly to capture and synthesise new research, preventing the need for many systematic reviews assessing the same research question at different points in time. Responses to our survey provided more insight into the reasons for duplication. Notably, users indicated that they did not identify any similar records when they searched PROSPERO prior to registration, despite our own simple repetition of their searches identifying similar reviews. This may indicate poor use of search terms that are not able or likely to identify similar protocols, or lack of examination of the reviews returned by their search (the very broad terms that some authors reported using would have returned a large number of potentially similar reviews that could have made screening a daunting task). To tackle this, PROSPERO may need to develop better tools and guidance to support more effective searching and continue to promote research integrity, such as making explanation of planned replication a mandatory requirement.

Given the time and resources required to conduct systematic reviews, a surprising number of duplicate reviews lacked external funding. Unfunded reviews may have been initiated due to an altruistic desire to do something to help, and at least early in the pandemic, many funders were not set up to make rapid funding decisions. The number of reviews lacking external funding may, however, be concerning as (at least in the case in high income countries), these reviewers may not have been subject to the same level scrutiny or review as afforded by the funding application process (e.g., demonstrating sufficient experience and a track record in conducting systematic reviews). Research funders may also require due diligence in checking for existing reviews. Research that assesses whether funded proposals translate into better quality research, compared to self-funded proposals could be useful.

Although not formally assessed here, a general observation from this research was the low quality of registration forms we reviewed. Poorly completed forms make it difficult, even for experienced systematic reviewers, to determine whether an existing review is similar or identical to their own and may therefore be contributing to unnecessary duplication of systematic reviews. Fewer high quality systematic reviews are always more valuable to decision makers than many of poor quality ones, which have the potential to damage the confidence of the research community in systematic reviews, which are viewed as the 'gold standard' of evidence-based research. [10]

PROSPERO could help tackle the concerns raised in this research by engaging in outreach activity to improve the quality of registration records and by enhancing/promoting the guidance provided on the PROSPERO website. Further developments aimed at reducing the level of duplication could include the provision of resources to help users decide whether duplication is necessary or useful, such as the checklist created by Tugwell et al (2021).[11] This would be especially helpful for

For peer review only - http://bmjopen.bmj.com/site/about/guidelines.xhtml

researchers who lack experience in systematic reviews and may not understand the importance of avoiding duplication. An improved functionality around discovery of similar reviews could be introduced, including the development of automation that would highlight potentially similar reviews that exist in the database. More generally, PROSPERO's educational role could be developed, not only in promoting best review methods and practice, but also in highlighting the importance of avoiding unintended duplication and research waste. With 175,140 signed up users (setting up a PROSPERO account is prerequisite to registering a review) we are well placed to reach a large audience and help build expertise and infrastructure that would better serve decision making in a future pandemic.

Limitations

As not all authors prospectively register their systematic reviews, the real extent of duplication may differ from that in PROSPERO, particularly because those who knowingly duplicate without good reason may be less likely to register their review.

Owing to time and resource constraints our research only examined four of the seventeen research areas related to COVID-19.

As records can be created in the PROSPERO system prior to submission, it is possible that similar systematic reviews could be registered during this period. If authors did not re-search PROSPERO prior to submission, unintended duplication could occur. However, this data is not available in the PROSPERO system so was not captured in this study.

We observed low quality registration forms and a substantial number of systematic reviews that lacked external funding; however, a comparison was not made to non-duplicate reviews. Further work addressing this could be of interest.

Finally, while the surveys provide a useful insight into the reasons for replication of previous systematic reviews, the number of responses received was small. This may not be representative of all PROSPERO users, as those who responded could feel that they had a legitimate reason to replicate, whilst those who did not may not have responded to the survey when asked.

Conclusions

This research has highlighted that registration of duplicate systematic reviews related to COVID-19 has occurred frequently in PROSPERO, with 138 review teams addressing research questions of

For peer review only - http://bmjopen.bmj.com/site/about/guidelines.xhtml

existing reviews in the first year of the pandemic. It adds to the evidence highlighting the problem of duplication and research waste in reviews and evidence syntheses and has identified a need to increase awareness of the need to avoid unintended duplication among those conducting reviews. PROSPERO, which has provided registration free of charge and served the systematic review community for ten years, responded to the COVID-19 pandemic by ensuring that reviews relating to COVID-19 were prioritised and registered/published rapidly. Changes made to accommodate this, along with associated reflection on the role of registration, has informed thinking about future developments and consideration of how PROSPERO can continue to support the global systematic review community over the next ten years and be part of the research infrastructure that stands ready to respond effectively to future global health challenges.

Contributorship Statement

The corresponding author attests that all listed authors meet authorship criteria and that no others meeting the criteria have been omitted.

Lucy Beresford and Ruth Walker equally contributed to the data curation, formal analysis, investigation, visualisation and writing of the original draft, Lesley Stewart provided support for these tasks. Lesley Stewart led the conceptualisation, project administration, validation and reviewing and editing of the drafts, Lucy Beresford and Ruth Walker provided support for these tasks. All authors contributed equally to the methodology used in the research. Lesley Stewart provided overall project supervision.

Funding Statement

This report presents independent research commissioned by the National Institute for Health Research (NIHR). The views and opinions expressed by authors in this publication are those of the authors and do not necessarily reflect those of the NHS, the NIHR, MRC, CCF, NETSCC, or the Department of Health.

Competing Interests

Production of PROSPERO is funded by the NIHR through an overarching research contract 14/25/13. Supplementary funding was made through this award to support PROSPERO’s response to COVID-19 which included this project. This research funding is made to the University of York. Lesley Stewart is the principal investigator and award holder. Ruth Walker is partially funded on this grant and is part of the PROSPERO team. Lucy Beresford was funded by the supplementary Covid funding for a period of six months to provide additional support to PROSPERO and contribute to this project.

For peer review only - http://bmjopen.bmj.com/site/about/guidelines.xhtml

Data Sharing Statement

Not applicable.

Ethics

The study was granted ethical approval from the University of York, Health Sciences Research Governance Committee. The study was deemed to be very low risk so was dealt with as a Chair's Action. The letter can be provided upon request.

Acknowledgements

We would like to acknowledge Gordon Dooley who extracted the data from PROSPERO for analysis.

For peer review only

References

1. Lorenc T KC, Raine G, Shemilt I, Sutcliffe K, D'Souza P, Burchett H, Hinds K, Macdowall W, Melton H, Richardson M, South E, Stansfield C, Thomas S, Kwan I, Wright K, Sowden A, Thomas J (2020) COVID. COVID-19: living map of the evidence. London: EPPI-Centre, Social Science Research Unit, UCL Social Research Institute, University College London., 2020.

2. Page MJ, Welch VA, Haddaway NR, et al. "One more time": why replicating some syntheses of evidence relevant to COVID-19 makes sense. *J Clin Epidemiol* 2020;125:179-82. doi: 10.1016/j.jclinepi.2020.05.024 [published Online First: 2020/05/29]

3. Page MJ, McKenzie JE, Bossuyt PM, et al. The PRISMA 2020 statement: an updated guideline for reporting systematic reviews. *Bmj* 2021;372

4. Booth A, Clarke M, Ghersi D, et al. An international registry of systematic-review protocols. *Lancet* 2011;377(9760):108-09. doi: 10.1016/s0140-6736(10)60903-8

5. Moher D, Booth A, Stewart L. How to reduce unnecessary duplication: use PROSPERO. *Bjog* 2014;121(7):784-6. doi: 10.1111/1471-0528.12657 [published Online First: 2014/03/19]

6. Stewart L, Moher D, Shekelle P. Why prospective registration of systematic reviews makes sense. *Systematic Reviews* 2012;1(1):7. doi: 10.1186/2046-4053-1-7

7. Glasziou PP, Sanders S, Hoffmann T. Waste in covid-19 research. *Bmj* 2020;369:m1847. doi: 10.1136/bmj.m1847 [published Online First: 2020/05/14]

8. Chalmers I, Glasziou P. Avoidable waste in the production and reporting of research evidence. *Lancet* 2009;374(9683):86-9. doi: 10.1016/s0140-6736(09)60329-9 [published Online First: 2009/06/16]

9. Tricco AC, Antony J, Zarin W, et al. A scoping review of rapid review methods. *BMC Medicine* 2015;13(1):224. doi: 10.1186/s12916-015-0465-6

10. Smith J, Noble H. Reviewing the literature. *Evidence Based Nursing* 2016;19(1):2. doi: 10.1136/eb-2015-102252

For peer review only - http://bmjopen.bmj.com/site/about/guidelines.xhtml

11. Tugwell P, Welch VA, Karunananthan S, et al. When to replicate systematic reviews of interventions: consensus checklist. *BMJ* 2020;370:m2864. doi: 10.1136/bmj.m2864

For peer review only

For peer review only - http://bmjopen.bmj.com/site/about/guidelines.xhtml

Figure Legends

- **Figure 1.** Number of COVID-19 related registrations between March 2020 to July 2021.
- **Figure 2.** Flow chart showing number of records screened and identified as ‘duplicates’ in the four COVID-19 research areas.
- **Figure 3.** Number of ‘duplicate’ reviews registered in PROSPERO between March 2020 and January 2021, by research area.
- **Figure 4.** The country in which the survey respondent’s review is/was being carried out.

For peer review only

Figure 1. *Number of COVID-19 related registrations between March 2020 to July 2021.*

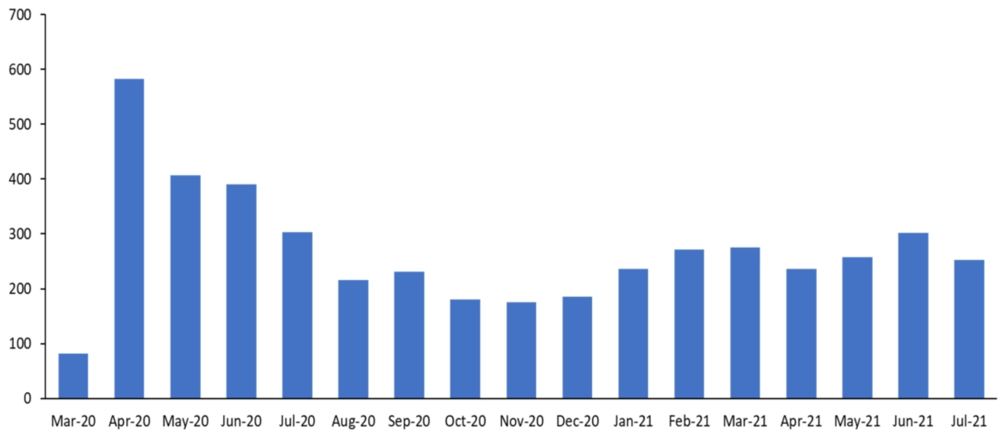

655x319mm (130 x 130 DPI)

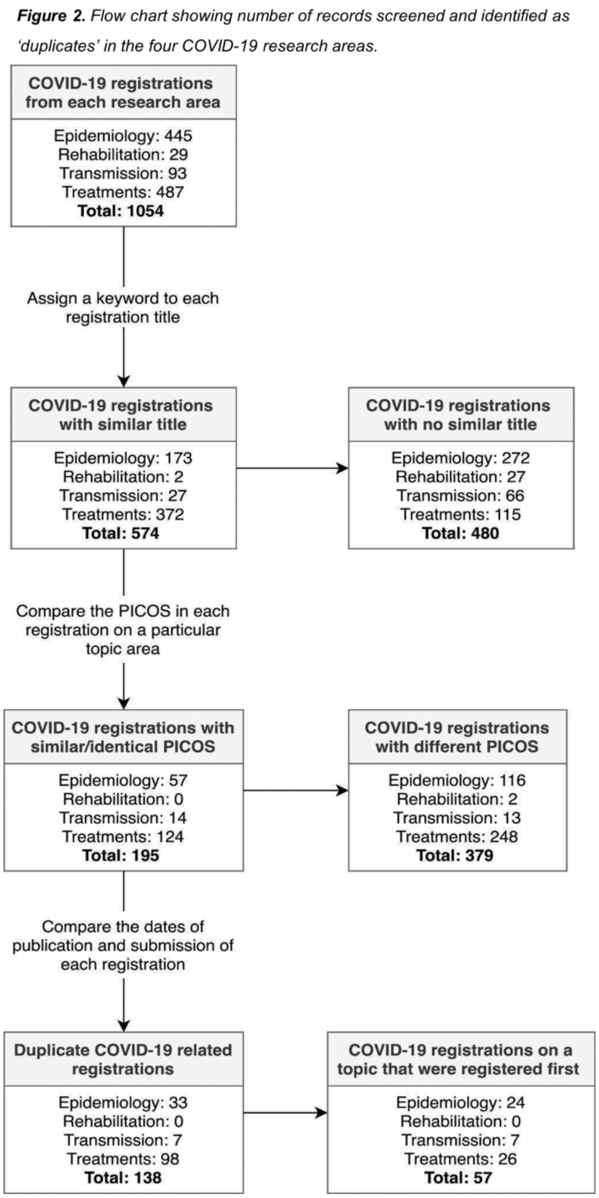

Figure 2. Flow chart showing number of records screened and identified as 'duplicates' in the four COVID-19 research areas.

287x582mm (130 x 130 DPI)

Figure 3. Number of 'duplicate' reviews registered in PROSPERO between March 2020 and January 2021 by research area

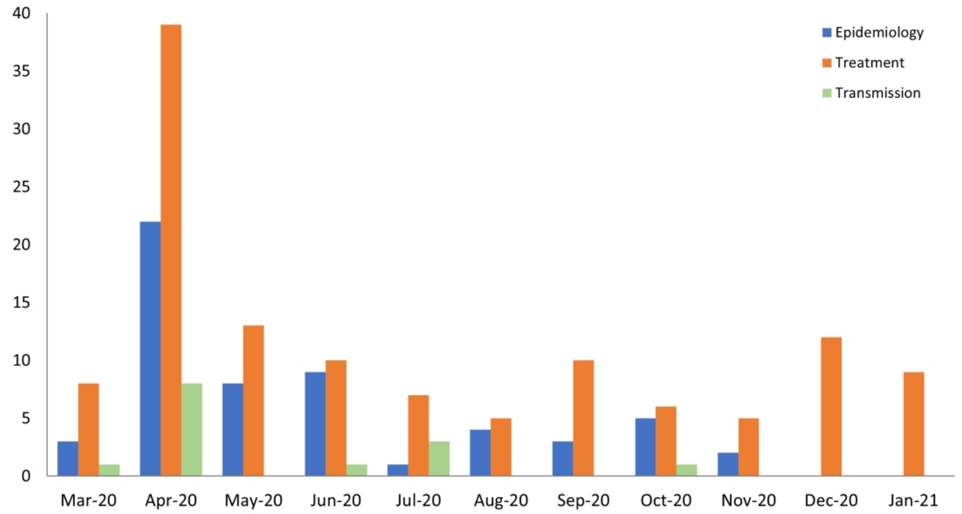

Please note: Registrations for transmission and epidemiology were obtained in November 2020. Registrations on treatment were obtained in January 2021. Therefore, in December 2020 and January 2021, only records relating to treatment of COVID-19 were reviewed. No 'duplicate'

499x311mm (130 x 130 DPI)

Figure 4. The country in which the survey respondent's review is being carried out

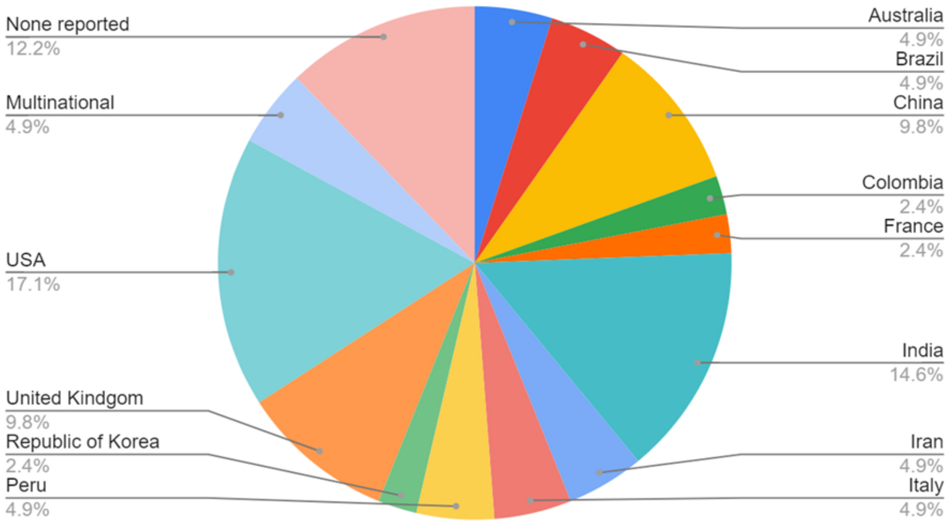

Figure 4. The country in which the survey respondent's review is/was being carried out.

622x385mm (38 x 38 DPI)

Appendix

Data Extraction

Table 1. *Data extraction template for identifying duplicate reviews*

Record number	Research area	Date submitted	Date registered	Is the PICOS similar/identical to existing review?	Answer to screening question? *	Is the review funded?	Have authors acknowledged similar reviews?	Authors reason for duplication?	Is there a reason for duplication from our perspective?

**Screening questions before authors submit a PROSPERO registration ask whether their review is not similar to an existing review, sufficiently different, or similar to an existing review, but repetition is needed*

COVID-19 Systematic Review Duplication in PROSPERO Questionnaire

For peer review only

The survey can be entirely anonymous. But it would be useful for us to be able to link your responses to the PROSPERO record, so you can choose to include your personal information. If you do provide your identifying ID number, this will not be included or used in any future publications and it will not affect the status of your PROSPERO registration.

This survey has been granted ethical approval. If you choose to include your PROSPERO registration number, your data will be handled in compliance with GDPR and the UK Data Protection Act (2018). For more information, please visit the links on the University of York's policy on Data Protection Legislation, GDPR compliant research and the general privacy notice.

Q1 What is your PROSPERO registration number

____

About your COVID-19 related systematic review

Q2 Have you conducted a systematic review before?

- Yes (1)
- No (2)
- Other (3) ____

Q3 What research area was your systematic review looking at?

- Epidemiology (1)
- Transmission (2)
- Treatments (3)
- Other (4) ____

Appendix

Q4 Why did you conduct your systematic review on a COVID-19 related topic?

- To inform future research that you had planned (such as a primary research study) (1)
- To inform funders/policy makers (2)
- For personal/departmental interest (3)
- Other (4) ____

Q5 Please provide details of the country in which the review is being carried out. For multi-national collaborations, give details of all the countries involved.

____

Q6 Is your review funded

- Yes (1)
- No (2)
- Other (3) ____

Display This Question:

If Is your review funded != No

Q7 If your review is funded, please provide details of the individuals, organizations, groups or other legal entities who take responsibility for initiating, managing, sponsoring and/or financing the review

____

Q8 Do you intend to publish or disseminate the results of your systematic review?

- Yes (1)
- No (2)
- Maybe (3)
- Other (4) ____

Display This Question:

If Do you intend to publish or disseminate the results of your systematic review? = No

Q9 Why did you decide not to publish the results of your systematic review?

____

Q10 Is there any other information that you wish to provide about your systematic review?

____

The following questions were specific to the registrant's response to the triage questions at PROSPERO submission

For peer review only

Appendix

Not Similar Sub-Questions

When registering, users are prompted to search PROSPERO and check whether a similar systematic review has been registered. Some duplication, intended with good reason (e.g. the existing one is out of date or used suboptimal methods), is justifiable. When registering, you indicated that your review was not similar to an existing review

Q12 To help us understand this further, please indicate which statement applies most to your situation.

- I searched PROSPERO prior to registration and identified some/all of the similar records (1)
- I searched PROSPERO prior to registration but did not identify the similar records (2)
- I did not search PROSPERO prior to registration (3)

Display This Question:

If To help us understand this further, please indicate which statement applies most to your situation. = I searched PROSPERO prior to registration and identified some/all of the similar records

Q13 Please could you tell us more about the reasons you conducted a review similar to those which you identified?

____

Display This Question:

If To help us understand this further, please indicate which statement applies most to your situation. = I searched PROSPERO prior to registration but did not identify the similar records

Q14 Please could you tell us more about how you searched PROSPERO, including the search terms you used. This could help us to improve our guidance about how to search PROSPERO in the future.

____

Display This Question:

If To help us understand this further, please indicate which statement applies most to your situation. = I did not search PROSPERO prior to registration

Q15 Please could you tell us more about the reasons you did not search PROSPERO before registration?

____

For peer review only

Appendix

Sufficiently Different Sub-Questions

When registering, users are prompted to search PROSPERO and check whether a similar systematic review has been registered. Some duplication, intended with good reason (e.g. the existing one is out of date or used suboptimal methods), is justifiable. When registering, you indicated that your review was similar to an existing review but sufficiently different to justify this review.

Q12 To help us understand this further, please indicate which statement applies most to your situation.

- I searched PROSPERO and identified some/ all of the existing reviews that were similar to my own. (1)
- Other (2)

Display This Question:

If To help us understand this further, please indicate which statement applies most to your situation. = I searched PROSPERO and identified some/ all of the existing reviews that were similar to my own.

Q13 For each of the similar reviews you identified, please could you tell us more about the reasons why your review is sufficiently different.

____

Display This Question:

If To help us understand this further, please indicate which statement applies most to your situation. = Other

Q14 Please could you tell us whether you searched PROSPERO, and if you did, please could you explain more about how you searched PROSPERO, including the search terms you used. This could help us to improve our guidance about how to search PROSPERO in the future.

____

For peer review only

Appendix

Repetition Needed Sub-Questions

When registering, users are prompted to search PROSPERO and check whether a similar systematic review has been registered. Some duplication, intended with good reason (e.g. the existing one is out of date or used suboptimal methods), is justifiable. When registering, you indicated that your review was similar to an existing review but repetition was needed.

Q12 To help us understand this further, please indicate which statement applies most to your situation.

- I searched PROSPERO and identified some/ all of the existing reviews that were similar to my own. (1)
- Other (2)

Display This Question:

If To help us understand this further, please indicate which statement applies most to your situation. = I searched PROSPERO and identified some/ all of the existing reviews that were similar to my own.

Q13 For each of the similar reviews you identified, please could you tell us more about the reasons why repetition was needed.

____

Display This Question:

If To help us understand this further, please indicate which statement applies most to your situation. = Other

Q14 Please could you tell us whether you searched PROSPERO, and if you did, please could you explain more about how you searched PROSPERO, including the search terms you used. This could help us to improve our guidance about how to search PROSPERO in the future.

____

For peer review only

Appendix

Duplicate Registrations Characteristics

Table 2: Number of 'duplicate' records registered in each COVID-19 related topic area.

Topic	N duplicates
**Epidemiology**	
**Health Condition and Impact on Outcome**	
Cancer	1
Cardiovascular disease	1
Diabetes	2
General Risk Factors	8
Obesity	3
Comorbidities	6
**Healthcare Workers**	
COVID-19 in healthcare workers	1
Mental Health in Healthcare Workers	1
**Clinical presentation of COVID-19**	
Echocardiographic Findings	1
Manifestations (Dermatological)	1
Manifestations (Taste and Smell)	1
Manifestations (Neurological)	1
Long-COVID	1
**Transmission of COVID-19**	
Facemasks	1
Pregnancy and mother-baby transmission	1
**Treatment of COVID-19**	
ACE-inhibitors	1
Metformin	1
**Total number of duplicates**	**31**
**Treatments**	
**All treatments**	
All treatment living	1
All treatment MA	7
All treatment umbrella	2
All treatment NMA	8
**Pharmacological interventions**	
ACE-inhibitors	6
Anticoagulants	1
Corticosteroids	4
Hydroxychloroquine	14
Interferon	2
Ivermectin	3
JAK inhibitors	1

Appendix

Topic	N duplicates
**Pharmacological interventions**	
lopinavir	3
RAAS	2
Remdisivir	6
Tocilizumab	7
**Non-pharmacological interventions**	
Blood plasma	8
ECMO	5
Prone position	6
**Total number of duplicates**	**86**
**Transmission**	
Mother-baby transmission	2
Transmission during Surgery	1
Weather	1
**Total number of duplicates**	**4**

For peer review only

Appendix

Research area	Date submitted	Date registered	Answer to screening question?	Have authors acknowledged similar reviews?	Authors reason for duplication?	Is there reason for duplication from our perspective?	Is the review funded?
**Cancer**	17/04/20	21/04/20	-	-	-	-	None
**Cancer**	22/04/20	22/04/20	NS	No	No	Perhaps, submitted one day after last was published	None
**Cardiovascular Disease**	14/04/20	20/04/20	-	-	-	-	None
**Cardiovascular Disease**	31/05/20	01/06/20	NS	No	No	No	National Natural Science Foundation of China
**Children**	15/04/20	21/04/20	-	-	-	-	None
**Children**	18/04/20	20/04/20	-	-	-	-	Nanchang University
**Children**	02/08/20	06/08/20	RN*	No	-	Potentially. Four months since previous systematic review	None

For peer review only

Appendix

Research area	Date submitted	Date registered	Answer to screening question?	Have authors acknowledged similar reviews?	Authors reason for duplication?	Is there reason for duplication from our perspective?	Is the review funded?
**Comorbidities: Impact on Mortality**	24/03/20	03/04/20	-	-	-	-	None
**Comorbidities: Impact on Mortality**	22/05/20	28/05/20		No	None	No	None
**Comorbidities: Impact on Mortality**	29/10/20	30/10/20	NS*	No	None	Perhaps- to update evidence (5 months after last review).	None
**Comorbidities: Prevalence**	27/03/20	06/04/20	-	-	-	-	Human Resources Development Operational Programme Grant
**Comorbidities: Prevalence**	26/04/20	28/04/20	NS*	No	No	No	None
**Comorbidities: Severe Outcomes and Death**	26/02/20	17/03/20	-	-	-	-	National Key R&D Program of China.

Appendix

Research area	Date submitted	Date registered	Answer to screening question?	Have authors acknowledged similar reviews?	Authors reason for duplication?	Is there reason for duplication from our perspective?	Is the review funded?
**Comorbidities: Severe Outcomes and Death**	26/03/20	16/04/20	SD	Yes	Yes: no risk of bias in CRD42020170315	Yes, CRD42020170315 do not report methods of quality assessment).	None
**Comorbidities: Severe Outcomes and Death**	31/03/20	30/04/20	NS	No	None	No	None
**Comorbidities: Severe Outcomes and Death**	13/04/20	16/04/20	NS	No	None	No	Second Affiliated Hospital of Nanchang University
**COVID-19 in Healthcare Workers**	06/04/20	15/04/20	--	-	-	-	None
**COVID-19 in Healthcare Workers**	27/04/20	28/04/20	NS	None	No	No	None
**Diabetes**	28/04/20	04/05/20	-	-	-	-	None
**Diabetes**	30/06/20	30/06/20	NS	No	None	No	Natural Science Foundation of Tianjan and Tianjan Education Committee

Appendix

Research area	Date submitted	Date registered	Answer to screening question?	Have authors acknowledged similar reviews?	Authors reason for duplication?	Is there reason for duplication from our perspective?	Is the review funded?
**Diabetes**	05/10/20	06/10/20	NS	No	None	Perhaps update of review potentially needed	None
**Echocardiographic Findings**	18/08/20	19/08/20	-	-	-	-	None
**Echocardiographic Findings**	19/09/20	22/09/20	NS	No	No	No	None
**Face Masks**	29/09/20	29/09/20	-	-	-	-	None
**Face Masks**	19/10/20	28/10/20	SD	No	No	Yes. Living systematic review	None
**General Risk Factors: Impact on Mortality**	26/03/20	03/04/20	-	-	-	-	None
**General Risk Factors: Impact on Mortality**	04/04/20	09/04/20	NS	None	No	Potentially- only one day after previous record was registered.	Wellcome Trust

Appendix

Research area	Date submitted	Date registered	Answer to screening question?	Have authors acknowledged similar reviews?	Authors reason for duplication?	Is there reason for duplication from our perspective?	Is the review funded?
**General Risk Factors: Impact on Poor Outcomes and Mortality**	27/02/20	03/04/20	-	-	-	-	National Natural Science Foundation
**General Risk Factors: Impact on Poor Outcomes and Mortality**	15/03/20	27/03/20	-	-	-	-	None
**General Risk Factors: Impact on Poor Outcomes and Mortality**	18/03/20	23/03/20	NS	None	No	No	Chinese Natural Science Foundation and the Training Program of Shanghai Health and Family Planning System
**General Risk Factors: Impact on Poor Outcomes and Mortality**	28/03/20	09/04/20	SD	Yes	Yes, provide additional analysis (regression)	Yes	None
**General Risk Factors: Impact on Poor Outcomes and Mortality**	12/04/20	16/04/20	NS	No	None	No	None

For peer review only - http://bmjopen.bmj.com/site/about/guidelines.xhtml

Appendix

Research area	Date submitted	Date registered	Answer to screening question?	Have authors acknowledged similar reviews?	Authors reason for duplication?	Is there reason for duplication from our perspective?	Is the review funded?
**General Risk Factors: Impact on Poor Outcomes and Mortality**	14/04/20	17/05/20	NS*	No	None	None	None
**General Risk Factors: Impact on Poor Outcomes and Mortality**	20/04/20	21/04/20	RN	No	None	None	None
**General Risk Factors: Impact on Poor Outcomes and Mortality**	06/05/20	06/05/20	SD	No	None	No	Department of Epidemiology, College of Public Health, University of Nebraska Medical
**General Risk Factors: Impact on Poor Outcomes and Mortality**	11/05/20	12/05/20	NS	No	None	No	None
**Long COVID**	15/10/20	23/10/20	-	-	-	-	None
**Long COVID**	31/10/20	02/11/20	SD	No	No	No	None
**Manifestations: Taste and Smell**	22/05/20	29/05/20	-	-	-	-	None

Appendix

Research area	Date submitted	Date registered	Answer to screening question?	Have authors acknowledged similar reviews?	Authors reason for duplication?	Is there reason for duplication from our perspective?	Is the review funded?
**Manifestations: Taste and Smell**	20/06/20	25/06/20	NS	No	None	None	Health Commission of Hunan Province and Human Pharmaceutical Association
**Mental Health in Healthcare Workers**	31/07/20	03/08/20	-	-	-	-	National Council for Scientific and Technological Development - CNPq, Brazilian Government
**Mental Health in Healthcare Workers**	19/10/20	22/10/20	NULL	No	No	No	None
**Neurological Manifestations**	22/05/20	01/06/20	-	-	-	-	None
**Neurological Manifestations**	02/06/20	02/06/20	NS	No	None	Perhaps, submitted one day after previous was published	None
**Obesity**	13/04/20	06/04/20	-	-	-	-	NIHR Applied Research and Care
**Obesity**	29/04/20	01/05/20	NS	None	None	No	University of Indonesia

Appendix

Research area	Date submitted	Date registered	Answer to screening question?	Have authors acknowledged similar reviews?	Authors reason for duplication?	Is there reason for duplication from our perspective?	Is the review funded?
**Obesity**	13/05/20	15/05/20	NS	No	None	None	Programme Széchenyi 20
**Obesity**	10/11/20	12/11/20	SD	No	No	Perhaps update of review potentially needed	None
**Pregnancy**	04/04/20	23/06/20	-	-	-	-	Partly funded by WHO
**Pregnancy**	13/04/20	17/04/20	-	-	-	-	None
**Pregnancy**	24/04/20	24/04/20	NS*	No	None	No	None
**Skin Manifestations**	23/06/20	25/06/20	-	-	-	-	None
**Skin Manifestations**	28/06/20	29/06/20	SD	Yes	Yes (none will be published before completion)	No	None
**Treatment: ACE Inhibitors and ARBs**	07/06/20	19/06/20	-	-	-	-	Major Project of National Science and Technology on New Drug etc.

Appendix

Research area	Date submitted	Date registered	Answer to screening question?	Have authors acknowledged similar reviews?	Authors reason for duplication?	Is there reason for duplication from our perspective?	Is the review funded?
**Treatment: ACE Inhibitors and ARBs**	03/07/20	08/07/20	SD	No	None	No	None
**Treatment: Metformin**	21/08/20	25/08/20	-	-	-	-	ARC-EM
**Treatment: Metformin**	12/09/20	15/09/20	RN	None	None	No	National Natural Science China
**Breastfeeding**	06/04/20	21/04/20	SD	References CRD420173886	Yes. But review is sufficiently so no duplication occurred.	Yes	None
**Breastfeeding**	11/04/20	20/04/20	N/A	-	-	-	None
**Breastfeeding**	17/07/20	20/07/20	SD	Not explicitly	No	No	None
**Breastfeeding**		10/10/20	NS	No	No	No comparator.	None
**Maternal to Fetal Transmission**	16/03/20	24/03/20	NS	-	-	-	N/A
**Maternal to Fetal Transmission**	22/06/20	03/07/20	SD	No	No	No	None

Appendix

Research area	Date submitted	Date registered	Answer to screening question?	Have authors acknowledged similar reviews?	Authors reason for duplication?	Is there reason for duplication from our perspective?	Is the review funded?
**Surgery**	02/04/20	22/04/20	NS	-	-	-	None
**Surgery**	25/04/20	28/04/20	NS	No	N/A	No	None
**Viral Shedding**	24/04/20	29/04/20	NS	-	-	-	None
**Viral Shedding**	22/06/20	22/06/20	NS*	Yes, acknowledge CRD420181914	Yes, previous registration deemed poor quality	Potentially- poor reporting in previous registration. Authors have given reason for duplication.	None
**Weather**	28/03/20	07/04/20	NS	-	-	-	None
**Weather**	01/04/20	08/04/20	NS	-	-	-	Federal University of Pará, Brazil.
**Weather**	27/04/20	30/04/20	NS	No	No	Yes, looking at seasons and times of year rather than climate conditions.	None

For peer review only

Appendix

Research area	Date submitted	Date registered	Answer to screening question?	Have authors acknowledged similar reviews?	Authors reason for duplication?	Is there reason for duplication from our perspective?	Is the review funded?
**Weather**	03/07/20	03/07/20	SD	No	No	Almost identical. No real reason for duplication.	None
**Ace inhibitors**	05/04/20	14/04/20	NS	-	-	-	Self funded
**Ace inhibitors**	13/04/20	17/04/20	NS*	-	-	-	None
**Ace inhibitors**	03/05/20	05/05/20	NS	No	NO	NO	None
**Ace inhibitors**	04-Mayx-20	04/05/20	NS	No	NO	NO	China Postdoctoral Science Foundation (2019M652593) and Henan Postdoctoral Science Foundation (1902006)
**Ace inhibitors**	08/05/20	11/05/20	SD	Partially	NO	NO	None

Appendix

Research area	Date submitted	Date registered	Answer to screening question?	Have authors acknowledged similar reviews?	Authors reason for duplication?	Is there reason for duplication from our perspective?	Is the review funded?
**Ace inhibitors**	13/05/20	14/05/20	NS	No	NO	NO	This study was supported by National Natural Science Foundation of China, 81970203; National Natural Science Foundation of China, 81570212; National Natural Science Foundation of China, 31800976 Chongqing Science and Health Joint Medical Research Project, 2018QNXM024
**Ace inhibitors**	27/07/20	28/07/20	NS	No	NO	NO	Qingsong Qin is sponsored by Department of education of Guangdong province, China
**Ace inhibitors**	03/08/20	04/08/20	SD	Partially	NO	NO	Disciplines Construction Project of PUMC (Grant number: 20192102). Chinese Academy of Medical Sciences, CAMS Innovation Fund for Medical Sciences (Grant number: 2018-I2M-1-002)

Appendix

Research area	Date submitted	Date registered	Answer to screening question?	Have authors acknowledged similar reviews?	Authors reason for duplication?	Is there reason for duplication from our perspective?	Is the review funded?
**All treatment living**	20/04/20	22/04/20	SD	-	-	-	None
**All treatment living**	27/04/20	28/04/20	SD	Partially	NO	No	This review received some funding from the Agence Nationale de la Recherche (ANR) and from the WHO
**All treatment MA**	07-Feb-20	31/03/20	NS	-	-	-	NA
**All treatment MA**	09/03/20	20/03/20	NS	No	No	No	This study will be funded by Research Funds of Shanghai Health and Family Planning commission (20184Y0022, 20194Y0007), Cultivation fund of clinical research of Renji hospital (PY2018-III-06), Clinical Pharmacy Innovation Research Institute of Shanghai Jiao Tong University School of Medicine (CXYJY2019ZD001, CXYJY2019QN004).

Appendix

Research area	Date submitted	Date registered	Answer to screening question?	Have authors acknowledged similar reviews?	Authors reason for duplication?	Is there reason for duplication from our perspective?	Is the review funded?
**All treatment MA**	22/03/20	24/03/20	NS	No	No	No	Shubham Misra is a recipient of DST-INSPIRE PhD Fellowship from Department of Science & Technology, Government of India
**All treatment MA**	07/04/20	17/04/20	NS	No	No	No	https://covid19.kacst.edu.sa/grants/
**All treatment MA**	20/04/20	22/04/20	SD	Partially	No	No	None
**All treatment MA**	05/05/20	06/05/20	NS	No	No	No	None
**All treatment MA**	13/10/20	16/10/20	NS	No	No	No	This work was funded by the Guidance Plan for Social Development of Taizhou Municipal Science and Technology (ssf20160141).
**All treatment MA**	14/11/20	07/12/20	NS	No	No	No	None
**All treatment MA**	05/01/21	08/01/21	Not in system	No	No	No	None

Appendix

Research area	Date submitted	Date registered	Answer to screening question?	Have authors acknowledged similar reviews?	Authors reason for duplication?	Is there reason for duplication from our perspective?	Is the review funded?
**All treatment NMA**	07/04/20	15/04/20	NS	NO	Yes		
**All treatment NMA**	12/04/20	16/04/20	SD	Partially	NA		
**All treatment NMA**	18/04/20	22/04/20	SD	Partially	No	No	The research project will be assisted with the by Griffith University Postgraduate Research Scholarship
**All treatment NMA**	19/04/20	20/04/20	NS	NO	No	No	None
**All treatment NMA**	23/04/20	23/04/20	NS	NO	No	No	Supported by the Programme of Introducing Talents of Discipline to Universities, China, the 111 Project, D18011)
**All treatment NMA**	27/04/20	27/04/20	SD	Partially	No	No	None
**All treatment NMA**	15/05/20	19/05/20	NS	NO	No	No	None
**All treatment NMA**	18/06/20	24/06/20	NS	NO	No	No	None

Appendix

Research area	Date submitted	Date registered	Answer to screening question?	Have authors acknowledged similar reviews?	Authors reason for duplication?	Is there reason for duplication from our perspective?	Is the review funded?
**All treatment NMA**	18/10/20	26/10/20	NS	NO	No	No	None
**All treatment umbrella**	13/05/20	20/05/20	NS	NA	NA	NA	NA
**All treatment umbrella**	07/09/20	07/09/20	NS	No	No	No	Indian Council of Medical Research
**All treatment umbrella**	04/10/20	05/10/20	NS	No	No	No	None
**Anticoagulant**	02/06/20	02/06/20	NS	-	-	-	
**Anticoagulant**	02/07/20	03/07/20	SD	Partially	No	No	None
**Anticoagulant**	14/10/20	15/10/20	NS	No	No	No	None
**Anticoagulant**	07/01/21	11/01/21	not in list	No	No	No	None
**Blood plasma**	12/03/20	24/03/20	NS	-	-	-	None

For peer review only

Appendix

Research area	Date submitted	Date registered	Answer to screening question?	Have authors acknowledged similar reviews?	Authors reason for duplication?	Is there reason for duplication from our perspective?	Is the review funded?
**Blood plasma**	28/03/20	08/04/20	SD	Partially		No reasons why sufficiently different.	Funded by the UK Forum
**Blood plasma**	02/04/20	02/04/20	NS	No	No	No	None
**Blood plasma**	06/04/20	15/04/20	SD	Partially	No	No reasons why sufficiently different.	None
**Blood plasma**	10/04/20	15/04/20	NS	No	No	No	Education Foundation of Chengdu University of Traditional Chinese Medic
**Blood plasma**	17/06/20	19/06/20	SD	Partially	No	No	None
**Blood plasma**	05/07/20	06/07/20	SD	Partially	No	No reasons why sufficently different.	None
**Blood plasma**	16/07/20	16/07/20	NS	No	No	No	None
**Blood plasma**	12/08/20	14/08/20	SD	Partially	No	No reasons why sufficently different.	None

Appendix

Research area	Date submitted	Date registered	Answer to screening question?	Have authors acknowledged similar reviews?	Authors reason for duplication?	Is there reason for duplication from our perspective?	Is the review funded?
**Blood plasma**	09/12/20	22/12/20	not in list	No	No	No	None
**Blood plasma**	16/12/20	17/12/20	not in list		No	No	None
**Blood plasma**	21/12/20	22/12/20	not in list	No	Potentially severe patient pop.	No	None
**Colchicine**	17/09/20	21/09/20					None
**Colchicine**	11/01/21	12/01/21	not in list	No	No	No	None
**Corticosteroids**	03/03/20	20/04/20	NS	-	-	-	None
**Corticosteroids**	20/04/20	21/04/20	NS	-	-	-	None
**Corticosteroids**	28/04/20	30/04/20	NS	No	No	No	None

For peer review only

Appendix

Research area	Date submitted	Date registered	Answer to screening question?	Have authors acknowledged similar reviews?	Authors reason for duplication?	Is there reason for duplication from our perspective?	Is the review funded?
**Corticosteroids**	06/05/20	06/05/20	SD	Partially	No	No	This work was supported by National Key Research and Development Project (20YFC2005700), Key Realm R&D Program of Guangdong Province (2019B030335001), and Medical Scientific Research Foundation of Guangdong Province of China (A2019407)
**Corticosteroids**	07/07/20	07/07/20	SD	Partially	No	No	None
**Corticosteroids**	23/09/20	23/09/20	SD	Partially	No	No	None
**Corticosteroids**	07/12/20	08/12/20	not in list	No	No	No	None
**Corticosteroids**	21/12/20	22/12/20	not in list	No	No	No	NaFoUniMedCovid19“ (FKZ: 01KX21) „CEO-Sys“
**Corticosteroids**	05/01/21	12/01/21	not in list	No	No	No	None

Appendix

Research area	Date submitted	Date registered	Answer to screening question?	Have authors acknowledged similar reviews?	Authors reason for duplication?	Is there reason for duplication from our perspective?	Is the review funded?
**ECMO**	02/04/20	14/04/20		-	-	-	None
**ECMO**	07/04/20	14/04/20		-	-	-	None
**ECMO**	16/04/20	17/04/20	NS	No	No	No	None
**ECMO**	03/05/20	04/05/20	SD	Partially	No	No	None
**ECMO**	15/06/20	18/06/20	NS	No	No	No	None
**ECMO**	19/06/20	22/06/20	NS	No	No	No	None
**ECMO**	17/09/20	21/09/20	NS	No	No	No	None
**ECMO**	22/11/20	23/11/20	NS	No	No	No	This work was supported by the research Grant 20YFC0841300 from Ministry of Science and Technology of the People's Republic of China.

Appendix

Research area	Date submitted	Date registered	Answer to screening question?	Have authors acknowledged similar reviews?	Authors reason for duplication?	Is there reason for duplication from our perspective?	Is the review funded?
**ECMO**	21/12/20	22/12/20	NS	No	No	No	None
**ECMO**	04/01/21	07/01/21	NS	No	No	No	None
**Hydroxychloroquine**	22-Feb-20	17/03/20		-	-	-	None
**Hydroxychloroquine**	29/03/20	16/04/20	NS	No	No	No	Major Projects of Science and Technology Innovation 2025 in Ningbo, China
**Hydroxychloroquine**	04/04/20	06/04/20	SD	Partially	No	No	NIHR Applied Research Collaboration East Midlands (ARC EM), UK
**Hydroxychloroquine**	06/04/20	07/04/20	SD	Partially	No	No	None
**Hydroxychloroquine**	06/04/20	09/04/20	NS	No	No	No	None
**Hydroxychloroquine**	07/04/20	17/04/20	NS	No	No	No	None
**Hydroxychloroquine**	10/04/20	21/04/20	SD	Partially	No	No	None

Appendix

Research area	Date submitted	Date registered	Answer to screening question?	Have authors acknowledged similar reviews?	Authors reason for duplication?	Is there reason for duplication from our perspective?	Is the review funded?
**Hydroxychloroquine**	14/04/20	17/04/20	SD	Partially	No	No	None
**Hydroxychloroquine**	20/04/20	21/04/20	SD	Partially	No	No	None
**Hydroxychloroquine**	27/04/20	28/04/20	NS	No	No	No	None
**Hydroxychloroquine**	22/05/20	22/05/20	SD	Partially	No	No	None
**Hydroxychloroquine**	10/08/20	12/08/20	NS	No	No	No	None
**Hydroxychloroquine**	19/08/20	19/08/20	NS	No	No	No	None
**Hydroxychloroquine**	12/09/20	17/09/20	NS	No	No	No	None
**Hydroxychloroquine**	12/09/20	15/09/20	SD	Partially	No	No	None
**Interferon**	26/03/20	26/03/20	NA	-	-	-	None
**Interferon**		07/04/20	NA	-	-	-	None

Appendix

Research area	Date submitted	Date registered	Answer to screening question?	Have authors acknowledged similar reviews?	Authors reason for duplication?	Is there reason for duplication from our perspective?	Is the review funded?
**Interferon**	17/11/20	20/11/20	SD	Partially	No	No	None
**Interferon**	21/11/20	03/12/20	NS	No	No	No	None
**Ivermectin**	08/07/20	09/07/20		-	-	-	-
**Ivermectin**	20/10/20	09/11/20	NS	no	No	No	None
**Ivermectin**	24/12/20	07/01/21	NS	no	No	No	The Institute for Health Technology Assessment (IETS),
**Ivermectin**	11/01/21	12/01/21	NS	no	No	No	None
**JAK inhibitors**	07/06/20	09/06/20		-	-	-	NA
**JAK inhibitors**	04/01/21	12/01/21	SD	partially	no	no	None
**lopinavir**	06/04/20	15/04/20	NA	NA	NA	NA	NA

Appendix

Research area	Date submitted	Date registered	Answer to screening question?	Have authors acknowledged similar reviews?	Authors reason for duplication?	Is there reason for duplication from our perspective?	Is the review funded?
**lopinavir**	09/04/20	15/04/20	NA	NA	NA	NA	NA
**lopinavir**	12/04/20	16/04/20	NA	NA	NA	NA	NA
**lopinavir**	17/04/20	21/04/20	NS	no	no	no	None
**lopinavir**	24/04/20	24/04/20	NS	no	no	no	None
**lopinavir**	13/01/21	15/01/21	SD	partially	no	no	This study is carried out at the request of the Ministry of Health and Social Protection and the Disaster Risk Management Fund. Contract No. 9677-MECOVID19-1009-20.
**Prone position**	24/06/20	24/06/20	NA	-	-	-	-
**Prone position**	28/08/20	01/09/20	NS	no	no	no	None
**Prone position**	11/09/20	14/09/20	NS	no	no	no	None

Appendix

Research area	Date submitted	Date registered	Answer to screening question?	Have authors acknowledged similar reviews?	Authors reason for duplication?	Is there reason for duplication from our perspective?	Is the review funded?
**Prone position**	19/10/20	22/10/20	NS	no	no	no	None
**Prone position**	16/11/20	20/11/20	RN	yes	no	no	None
**Prone position**	10/12/20	11/12/20	NS	no	no	no	None
**Prone position**	12/12/20	14/12/20	NS	no	no	no	None
**RAAS**	07/05/20	11/05/20		-	-	-	None
**RAAS**	14/06/20	17/06/20	NS	no	no	no	None
**RAAS**	15/09/20	16/09/20	RN	yes	no	no	None
**Remdisivir**	08-Feb-20	23/03/20	NS	-	-	-	National Natural Science Foundation of China
**Remdisivir**	02/04/20	14/04/20	NS	No	No	No	CDT-Africa, College of Health Sciences, Addis Ababa University
**Remdisivir**	21/04/20	23/04/20	NS	No	No	No	None

Appendix

Research area	Date submitted	Date registered	Answer to screening question?	Have authors acknowledged similar reviews?	Authors reason for duplication?	Is there reason for duplication from our perspective?	Is the review funded?
**Remdisivir**	30/04/20	01/05/20	SD	No	No	No	None
**Remdisivir**	26/05/20	26/05/20	NS	No	No	No	Tongji Hospital, Tongji Medical College
**Remdisivir**	20/09/20	21/09/20	NS	No	No	No	None
**Remdisivir**	12/10/20	15/10/20	NS	No	No	No	Zhejiang Medicine and Health Science and Technology Project; Natural Science Foundation of Zhejiang Province
**Tocilizumab**	22/03/20	24/03/20	NS	-	-	-	None
**Tocilizumab**	18/06/20	19/06/20	NS		No	No	None
**Tocilizumab**	21/06/20	22/06/20	SD		No	No	None

Appendix

Research area	Date submitted	Date registered	Answer to screening question?	Have authors acknowledged similar reviews?	Authors reason for duplication?	Is there reason for duplication from our perspective?	Is the review funded?
**Tocilizumab**	26/06/20	01/07/20	NS		No	No	Nicola Luigi Bragazzi and Jianhong Wu are partially supported by the Canadian Institute of Health Research (CIHR) 2019 Novel Coronavirus (COVID-19) rapid research program
**Tocilizumab**	14/08/20	17/08/20	NS		No	No	None
**Tocilizumab**	06/11/20	10/11/20	NS	no	No	No	None
**Tocilizumab**	11/12/20	18/12/20	NS	no	No	No	1.3.5 project for disciplines of excellence, West China Hospital, Sichuan University
**Tocilizumab**	16/12/20	17/12/20	SD	Partially	No	No	None

* Registrant changed their answer to the screening question. Grey cells represent 'duplicate' records, white cells represent records registered first.
Abbreviations: NS, Not Similar; RN, Repetition Needed; SD, Sufficiently Different.

BMJ Open

The extent and nature of duplication in PROSPERO: an investigation using COVID-19 related registrations

Journal:	*BMJ Open*
Manuscript ID	bmjopen-2022-061862.R1
Article Type:	Original research
Date Submitted by the Author:	26-Jul-2022
Complete List of Authors:	Beresford, Lucy; University of York, Centre for Review and Dissemination<br>Walker, Ruth; University of York Centre for Reviews and Dissemination<br>Stewart, Lesley; University of York Centre for Reviews and Dissemination
**Primary Subject Heading**:	Research methods
Secondary Subject Heading:	Infectious diseases
Keywords:	COVID-19, QUALITATIVE RESEARCH, STATISTICS & RESEARCH METHODS

SCHOLARONE™ Manuscripts

I, the Submitting Author has the right to grant and does grant on behalf of all authors of the Work (as defined in the below author licence), an exclusive licence and/or a non-exclusive licence for contributions from authors who are: i) UK Crown employees; ii) where BMJ has agreed a CC-BY licence shall apply, and/or iii) in accordance with the terms applicable for US Federal Government officers or employees acting as part of their official duties; on a worldwide, perpetual, irrevocable, royalty-free basis to BMJ Publishing Group Ltd ("BMJ") its licensees and where the relevant Journal is co-owned by BMJ to the co-owners of the Journal, to publish the Work in this journal and any other BMJ products and to exploit all rights, as set out in our licence.

The Submitting Author accepts and understands that any supply made under these terms is made by BMJ to the Submitting Author unless you are acting as an employee on behalf of your employer or a postgraduate student of an affiliated institution which is paying any applicable article publishing charge ("APC") for Open Access articles. Where the Submitting Author wishes to make the Work available on an Open Access basis (and intends to pay the relevant APC), the terms of reuse of such Open Access shall be governed by a Creative Commons licence – details of these licences and which Creative Commons licence will apply to this Work are set out in our licence referred to above.

Other than as permitted in any relevant BMJ Author's Self Archiving Policies, I confirm this Work has not been accepted for publication elsewhere, is not being considered for publication elsewhere and does not duplicate material already published. I confirm all authors consent to publication of this Work and authorise the granting of this licence.

For peer review only

Manuscript Title: **The extent and nature of duplication in PROSPERO: an investigation using COVID-19 related registrations**

Authors:

Miss Lucy Beresford, Research Fellow in Evidence Synthesis[1]
lucy.beresford@york.ac.uk*

Ruth Walker, Research Fellow in Evidence Synthesis[1]
ruth.walker@york.ac.uk*

Professor Lesley Stewart, Professor of Evidence Synthesis[1]
lesley.stewart@york.ac.uk

[1] Centre for Reviews and Dissemination, University of York
* These authors contributed equally to the work

Contact information for corresponding author

Ruth Walker, Research Fellow in Evidence Synthesis
Centre for Reviews and Dissemination, University of York
ruth.walker@york.ac.uk

For peer review only

For peer review only - http://bmjopen.bmj.com/site/about/guidelines.xhtml

Abstract

Introduction

During COVID-19, PROSPERO (an international prospective register of systematic reviews) experienced a surge in registrations for COVID-19 related systematic reviews, and duplication of research questions became apparent. Duplication can waste funding, time and research effort and can make policy-making more difficult.

Objectives

This project explored the extent of and reasons for duplication of COVID-19 related systematic review registrations in PROSPERO during the pandemic.

Methods

We searched PROSPERO for registrations related to four COVID-19 research areas: epidemiology, rehabilitation, transmission, and treatments. Records identified were compared using the PICOS (Population, Intervention, Comparator, Study Design) elements of the PROSPERO registration form. Registrations that had similar or identical PICOS were retained for further evaluation as 'duplicates'.

Named contact authors of 'duplicate' registrations were invited to complete a survey asking whether they searched PROSPERO prior to registration, identified the similar reviews, and if so, why they continued with their review.

Results

1054 COVID-19 reviews were registered between March 2020 and January 2021, of which 138 were identified as having been submitted when at least one similar protocol was already registered in PROSPERO. Duplication was greatest in reviews of COVID-19 treatments; for example, there were 14 similar reviews evaluating the efficacy of hydroxychloroquine.

From the 138 authors invited to take part in the survey, we received 41 responses. Most respondents said that they identified similar reviews when they searched PROSPERO prior to registration. The main reasons they gave for 'duplication' were differences in PICOS or planned analyses (N = 13), poor quality of previous registrations (N = 2), and the need to update the evidence (N = 3).

For peer review only - http://bmjopen.bmj.com/site/about/guidelines.xhtml

Conclusions

This research highlights that registration of similar and duplicate systematic reviews related to COVID-19 in PROSPERO occurred frequently. Increased awareness of research waste is required, and initial checking for similar reviews should be embedded within good review practice.

Strengths and Limitations

- As this research project was conducted by members of the PROSPERO team, we were able to access data not publicly available – specifically whether authors identified other similar reviews prior to registration. Therefore, this paper provides a unique insight into the nature of duplication in PROSPERO.
- Prior to conducting the full analysis, we were able to pilot the screening and data extraction process in duplicate. This allowed us to iron out discrepancies in our methods and technique prior to singular screening.
- We received a relatively small number of responses to our survey (30%). While the questionnaire provides useful insights into the reasons for duplication, it may not be representative of all those who registered 'duplicate' reviews.
- Owing to resource constraints, the study only explored the extent and nature of duplication from March 2020 until January 2021. Therefore, key milestones in the pandemic (such as the global role out of vaccinations, or the availability of antivirals) that could have impacted the extent of duplication were not explored.
- Resource constraints meant we could only evaluate the extent and nature of duplication in four of the seventeen COVID-19 related topic areas. Although we attempted to choose a broad range of topics, the extent of duplication could differ in other COVID-19 research areas.

For peer review only

Introduction

During the COVID-19 pandemic, a significant volume of medical research has been conducted to better understand the virus, its biology and associated disease.[1] Large numbers of systematic reviews have synthesised the emerging evidence.[2] It is best practice to register systematic reviews at the protocol stage to help avoid unplanned duplication and to enable comparison between methods reported in the final publication and that which was planned in the protocol.[3] This can be done in registries such as PROSPERO, an international prospective register of systematic review protocols, funded by the National Institute of Health and Care Research (NIHR) in the UK. PROSPERO was launched in 2011 with the intention of increasing transparency, limiting unintended duplication, and reducing the risk of bias in systematic reviews. [4-6]

In the ten years since its launch in 2011, prospective registration of systematic reviews has become routine and the importance of this, acknowledged widely. At launch, PROSPERO anticipated registration of around 2000 systematic reviews per year. In 2020 40,639 reviews were registered, and PROSPERO now includes over 125,000 registration records. PROSPERO has global reach, with registrations originating from 194 countries and territories in 2020. In March 2020, PROSPERO prioritised registration of review protocols related to COVID-19. Our intention was to support the global pandemic research endeavour by making information about planned and ongoing reviews public as quickly as possible and providing researchers and policy makers with an overview of the evidence synthesis pipeline. This would also help reduce unintended duplication, by enabling authors to check whether similar reviews already existed before embarking on a new one, based on up-to-date information (COVID-19 registrations are published within 24 hours of submission Monday- Friday). Submissions are registered in PROSPERO through the process described in Box 1.

To aid identification of reviews on topics of key relevance to pandemic research, a single click search by broad COVID-19 research categories was implemented on the PROSPERO home page. The research categories have been regularly updated as new topics emerge. PROSPERO encourages authors not to duplicate existing systematic reviews but recognises that duplication may be intended with good reason (e.g., the existing review is out of date or used suboptimal methods). As such, when registering, users are asked to indicate whether their review is (i) not similar to an existing review, (ii) sufficiently different, or (iii) similar to an existing review, but repetition is needed, as part of a series of eligibility questions before completing the registration form.

For peer review only - http://bmjopen.bmj.com/site/about/guidelines.xhtml

Box 1.

- Registration in PROSPERO involves the submission and publication of key information about the design and conduct of a systematic review
- Authors should register their systematic review once they have designed their review protocol but before they begin data extraction
- Prior to submission authors answer a series of 'triage questions' to check whether their review is eligible for inclusion.
- Previously, applications were checked by the PROSPERO team to ensure that they were in scope and that the required data had been provided. Since March 2020, to enable rapid processing of COVID-19 registrations, some applications have been published automatically following simple automated checks. No quality assessment or peer review is involved. Records are published on an open access electronic database.
- Registration information can be amended by authors should plans change. These changes are published, and a date stamped audit trail of previous versions made available in the public record.
- Registration records are permanent, and authors are asked to provide links to subsequent reports and publications.
- PROSPERO assigns each registered review a unique registration number. This number should be cited in publications.
- Peer reviewers and readers can compare what was reported for the review with what was intended in the review protocol.

Despite encouragement to check PROSPERO for similar systematic reviews and to not duplicate without good reason, PROSPERO administrators quickly raised the issue that multiple systematic reviews related to COVID-19, addressing the same research question, were being registered. Unnecessary duplication of research is wasteful and creates additional work for healthcare providers who need to make sense of evidence in a rapidly evolving field. [7,9] Duplication can also cause confusion, if findings between reviews are inconsistent.[2]

Using retrospective analyses, we aimed to explore the extent of duplication within PROSPERO registration records related to COVID-19, including whether the extent differed between rapid, full and living systematic reviews and/or particular areas of COVID-19 related research. We also aimed to explore the nature of duplication, including whether this was done unknowingly, covertly or for good reason by authors who were open and transparent about replication. Finally, we aimed to better understand the reasons for repetition from the authors' perspective and explore who funded duplicate reviews by conducting a survey.

Methods

Searches

PROSPERO was searched for registrations relating to COVID-19 from March 2020 to January 2021. PROSPERO created a search strategy for users to identify COVID-19 related records, which was used in this research project (see Appendix A). When registering their review, authors chose their 'type

For peer review only - http://bmjopen.bmj.com/site/about/guidelines.xhtml

and method of review' from a list. These tags then acted as filters on the PROSPERO website to identify the eligible studies. Due to resource constraints, not all research areas could be assessed. Four of the seventeen COVID-19 research categories were selected for further study: epidemiology, transmission, treatment, and rehabilitation. These research categories were chosen to capture a wide range of different topic areas addressing questions relating to COVID-19. For practical reasons, the records were downloaded for reviews of transmission, rehabilitation and epidemiology in November 2020, and treatment in January 2021.

Identification of duplicate records

Within each topic area, records were each assigned a keyword based on the review title. Those with the same keyword were compared, firstly using the title of the registration record and then for those with similar titles, using the Population, Intervention/Exposure, Comparator, Outcome and Study design (PICOS) criteria recorded in the PROSPERO registration form. Those addressing the same research question, based on the PICOS criteria were retained (see Figure 2). To ensure that decisions were being made consistently, this was conducted in duplicate by two reviewers for a subset of records (the rehabilitation and transmission reviews, and a random sample of epidemiology registrations (100 from 445 records)). Discrepancies in decisions were discussed between reviewers to reach a consensus. The remaining records were screened singularly.

For reviews addressing the same research question, the date of first submission to PROSPERO and the date of publication were obtained. Registrations submitted after the publication of a record with a similar or identical PICOS, were deemed 'duplicates' and retained. Reviews that have been submitted to PROSPERO but not yet registered/published on the public website (for example if the record was returned to authors for additional information or clarification) are not visible on the PROSPERO COVID-19 search. Therefore, if a record was *submitted* before another record on the same topic and *published* after it, this was not counted as duplication because the authors would not have had knowledge of the other review at the point they submitted their registration form.

Data extraction

For duplicate reviews, data on whether authors acknowledged similar existing registrations in PROSPERO when answering the screening questions prior to registration were recorded. From the public registration forms, data concerning the nature of duplication were extracted (see Appendix B, for Data Extraction template). This included whether the authors provided reasons for conducting a new review and whether they had an external funding source. Furthermore, we extracted information about whether reviews were rapid, full, or living systematic reviews. These topics were

For peer review only - http://bmjopen.bmj.com/site/about/guidelines.xhtml

defined based on whether the PROSPERO record included the wording 'living' or 'rapid' in their record, if not they were deemed to be 'full' systematic reviews. This information was used to assess whether full systematic reviews were conducted after rapid reviews for particular research topics, and/or whether rapid or full systematic reviews were being registered after living systematic reviews.

Surveys

To further understand the reasons why people duplicated existing reviews, authors of duplicate registrations were contacted using the email address of the named contact, provided in the PROSPERO form, and invited to complete a short questionnaire. Authors were asked whether they searched PROSPERO prior to registration, and whether they identified any similar reviews. Authors were also asked why they believed their review to be sufficiently different from an existing review, or why they felt repetition was needed if they had identified a similar review. The contacted authors had up to six weeks to respond to the survey, and reminders to complete the questionnaire were sent one week before it closed. A copy of the questionnaire is given in Appendix C.

Patient Public Involvement

Patients or the public were not involved in the design, conduct or reporting, or dissemination plans of our research. Our findings from this research were discussed with the COVID Evidence Network to support Decision making (COVID-END) community.

Results

Search results

From 01 March 2020 to 31st of January 2021, PROSPERO registered 3,013 protocols related to COVID-19. The monthly number of registrations published on PROSPERO peaked in April 2020 with 588 protocols (Figure 1). Overall, 1054 registrations related to the four COVID-19 topic areas of interest (until January 2021).

Descriptive characteristics

574 records were found to have a similar review title and had their full PROSPERO record examined. 195 of these records had a similar or identical PICO(S) (57 of which were the first instance of reviews addressing particular research questions). The remaining 138 duplicate records had been submitted when at least one protocol with similar or identical PICOS had already been published in PROSPERO. Figure 2 presents the number of records at each stage of the screening process. The data from the extracted duplicate studies are provided in Appendix D.

For peer review only - http://bmjopen.bmj.com/site/about/guidelines.xhtml

There were 33 (7.4% of those registered) duplicate reviews for epidemiological topics, 98 (20%) for treatments and seven (7.5%) relating to the transmission of COVID-19. No duplicate reviews were identified in the rehabilitation topic area. Figure 3 shows the number of duplicated reviews registered by month between March 2020 and January 2021 in each research area.

The number of duplicate reviews on specific questions ranged from one to 14. Multiple duplicate registrations were most common in reviews evaluating COVID-19 treatments, including reviews evaluating the efficacy of drugs such as hydroxychloroquine (14 duplicate reviews), tocilizumab (seven duplicate reviews), remdesivir (six duplicate reviews), the use of blood plasma (seven duplicate reviews) and prone position (six duplicate reviews). The majority of epidemiological reviews only had a single duplicate review, except for those evaluating the impact of comorbidities (six duplicate reviews) and reviews of general risk factors in patients with COVID-19 (eight duplicate reviews). The number of duplicates per research question are given in the Appendix E,.

Screening questions

When answering the PROSPERO screening questions prior to registering their protocol, authors of 85 of the 138 records considered as duplicate reviews did not acknowledge existing similar reviews in PROSPERO and selected 'Not Similar' on the screening question. Forty-seven indicated that their review was sufficiently different to those already registered and five authors indicated that repetition of existing reviews was needed.

Review type

Table 1 shows the number of registrations published on PROSPERO by research area and review type. We identified 138 duplicate full systematic reviews, only two of which were replacing rapid reviews that addressed the same research question.

Table 1. Number of Registered and Duplicate Reviews in four COVID-19 topic areas by type of systematic review.

Review Type	Number of Registered Reviews				
	**Epidemiology**	**Rehabilitation**	**Treatment**	**Transmission**	**Total**
**Number of Registered Reviews**					
**Rapid**	16[a]	2	21[b]	7	**46**
**Living**	12	3	27	7	**49**
**Full**	417	24	439	79	**520**
**Total**	**445**	**29**	**487**	**93**	**615**
**Number of Duplicate Reviews**					

For peer review only

**Rapid**	1	0	2	0	**3**
**Living**	1	0	6	1	**8**
**Full**	31	0	90	6	**127**
**Total**	**33**	**0**	**98**	**7**	**138**

[a]Two reviews were rapid, living systematic reviews and are counted in the 'living' row. [b]One review was a rapid, living systematic review and is counted in the 'living' row.

It was often difficult to differentiate between rapid and full systematic reviews as many of the registration forms were poorly completed. Some reviews also indicated they would complete within 4-6 weeks but did not identify as being a rapid review or detail abbreviated or rapid review methods, such as use of text-mining during the screening process.

Twenty-three full systematic reviews and one living systematic review were found to be registered after living systematic reviews addressing the same research question.

Reasons for duplication

Provided in registrations

For each duplicate record, we explored whether the authors had acknowledged previous registrations in their PROSPERO record, and if so, gave reasons for duplication. Thirty-three (24%) authors of duplicate records did so. Only 4 of these authors (12%) provided a reason for replication within their record despite being advised during the registration process that the reasons for duplication should be made clear.

Provided in response to survey

We received 41 unique responses to the survey investigating reasons for duplication, from 12 countries (Figure 4). Nineteen responses (22% of those contacted) were from authors who during PROSPERO screening indicated that their review was 'not similar' to existing registered reviews, 20 responses, (42% of those contacted) indicated their review was 'sufficiently different' and two responses (40% of those contacted) indicated 'repetition was needed'.

Of the respondents that provided their registration number in the survey, 68% had registered reviews relating to treatments for COVID-19. Overall, 90% of respondents had conducted a systematic review previously, and 92.5% intended to publish the results of their review. Most respondents had received no specific funding for their systematic review (87.5%).

Of the 11 respondents who indicated their review was 'not similar' to existing registered reviews, eight responded in the survey that they searched PROSPERO and did not identify any reviews on the same topic area. When asked to provide search terms used to do this, some were so broad that a

For peer review only - http://bmjopen.bmj.com/site/about/guidelines.xhtml

large number of results would have been returned (such as single words like ‘COVID-19’). Other respondents used multiple search terms; when we repeated these searches, all identified the duplicate records related to their review. Eight respondents said they searched PROSPERO and found some reviews on the same topics, but decided their review was different to the existing reviews found by their search.

All nineteen respondents who indicated in the screening questions that their reviews were sufficiently different to other registered reviews responded that their searches identified some or all the reviews that we identified as being similar to their own. However, no respondent provided justification to why they believed their review was significantly different to previous registrations in their PROSPERO registration.

Of the two respondents who indicated that repetition was needed, neither provided their justification for repeating previous systematic reviews.

The reasons given for conducting similar reviews are summarised in Table 2.

Table 2. *Summary of reasons for duplications from respondents of the questionnaire*

Response to Screening Question	Responses in Survey
**Not Similar**	• Different objectives or planned analyses<br>• Poor quality of previous systematic reviews<br>• Duplication was needed owing to the rapid rate that new studies on COVID-19 were being published
**Sufficiently Different**	• Differences in PICOS, study design or planned analyses<br>• Differences in databases searched<br>• Poor quality of previous systematic reviews<br>• Duplication was needed owing to the rapid rate that new studies on COVID-19 were being published
**Repetition Needed**	• No systematic review was published or completed at the time of registration<br>• Repetition was needed to confirm/refute previous systematic review conclusions*

<u>Discussion</u>

To synthesise the considerable amount of primary research conducted during the COVID-19 pandemic[8], a substantial number of systematic reviews have been produced, many of which have been prospectively registered on PROSPERO. There is growing concern that these include duplicate systematic reviews that have replicated without good reason. Unintended or unnecessary

For peer review only - http://bmjopen.bmj.com/site/about/guidelines.xhtml

duplication of research wastes research funding, time, and effort and creates extra work for policy makers and healthcare providers who need to determine what novel information, if any, new reviews provide. [2,5,10]

We aimed to assess the extent and nature of duplication within PROSPERO registration records related to COVID-19, within four research areas: epidemiology, rehabilitation, transmission, and treatment. Duplication was found to be extensive (accounting for 13% of records across these research areas). Researchers may have instead made a more valuable contribution to knowledge by considering alternative review topics (as advised on the PROSPERO homepage).

The nature of this duplication was first examined by assessing whether duplication was a result of rapid reviews (a form of evidence synthesis in which some systematic review methods are condensed or omitted to produce results in a timely manner) being replaced by full systematic reviews.[11] As rapid reviews provide a useful overview of the available research but may stop short of providing a full or detailed synthesis, a later full systematic review may be justified. However, only two examples of this were identified in our sample. We also identified rapid and full systematic reviews registered after living systematic reviews on the same topic. For example, following the registration of a living systematic review and network meta-analysis on all interventions for COVID-19, an additional seven duplicate standard systematic reviews were registered. This is particularly wasteful as living reviews update regularly to capture and synthesise new research, preventing the need for many systematic reviews assessing the same research question at different points in time. Living reviews are particularly important in situations, such as the COVID-19 pandemic, where the research evolves rapidly and published standard systematic reviews may become out of date quickly. [12]

Responses to our survey provided more insight into the reasons for duplication. Notably, users indicated that they did not identify any similar records when they searched PROSPERO prior to registration, despite our own simple repetition of their searches identifying similar reviews. This may indicate poor use of search terms that are not able or likely to identify similar protocols, or lack of examination of the reviews returned by their search (the very broad terms that some authors reported using would have returned a large number of potentially similar reviews that could have made screening a daunting task). To tackle this, PROSPERO may need to develop better tools and guidance to support more effective searching and continue to promote research integrity, such as making explanation of planned replication a mandatory requirement.

For peer review only - http://bmjopen.bmj.com/site/about/guidelines.xhtml

Whilst some valid reasons for duplication of systematic reviews were provided in the survey, for example, difference in the PICOS criteria and poor quality of previous reviews, these explanations were not included in the public PROSPERO registration forms, and therefore this detail can not inform decisions in future research for example, umbrella reviews that to synthesise outcome data from these systematic reviews.

Given the time and resources required to conduct systematic reviews, a surprising number of duplicate reviews lacked external funding. Unfunded reviews may have been initiated due to an altruistic desire to do something to help, and at least early in the pandemic, many funders were not set up to make rapid funding decisions. The number of reviews lacking external funding may, however, be concerning as, at least in the case in high income countries, these reviewers may not have been subject to the same level scrutiny or review as afforded by the funding application process (e.g., demonstrating sufficient experience and a track record in conducting systematic reviews). Research funders may also require due diligence in checking for existing reviews. Research that assesses whether funded proposals translate into better quality research, compared to self-funded proposals could be useful.

Although not formally assessed here, a general observation from this research was the low quality of registration forms we reviewed. Poorly completed forms make it difficult, even for experienced systematic reviewers, to determine whether an existing review is similar or identical to their own and may therefore be contributing to unnecessary duplication of systematic reviews. Fewer high quality systematic reviews are always more valuable to decision makers than many of poor-quality ones, which have the potential to damage the confidence of the research community in systematic reviews, which are viewed as the 'gold standard' of evidence-based research.[13,14]

Journals may play a role in preventing systematic reviews that have been unnecessarily duplicated from being published. However, whilst some journals now require PROSPERO registration as a prerequisite for publication, peer reviewers or editors may not have the resource, nor deem it necessary to search PROSPERO for similar reviews to ascertain whether there is a need for a new systematic review. Issues around the low quality of registration forms, as previously discussed, may also impede the ability to do this and inform the editorial decisions. Further research may involve conducting a search for published reviews arising from the duplicated protocols identified in this research, to better understand how many make it to publication.

PROSPERO could help tackle the concerns raised in this research by engaging in outreach activity to improve the quality of registration records and by enhancing/promoting the guidance provided on

For peer review only - http://bmjopen.bmj.com/site/about/guidelines.xhtml

the PROSPERO website. Further developments aimed at reducing the level of duplication could involve the provision of resources to help users decide whether duplication is necessary or useful, such as the checklist created by Tugwell et al (2021).[15] This could include information on the purpose of living systematic reviews and why duplication might be unnecessary after a research question has been addressed with this methodology. This advice would be especially helpful for researchers who lack experience in systematic reviews and may not understand the importance of avoiding duplication. An improved functionality around discovery of similar reviews could be introduced, including the development of automation that would highlight potentially similar reviews that exist in the database. If similar reviews are identified, additional information from the author could be requested, including the purpose and justification for duplication and the added value of their systematic review. More generally, PROSPERO's educational role could be developed, not only in promoting best review methods and practice, but also in highlighting the importance of avoiding unintended duplication and research waste. With 175,140 signed up users (setting up a PROSPERO account is prerequisite to registering a review) we are well placed to reach a large audience and help build expertise and infrastructure that would better serve decision making in a future pandemic.

Limitations

As not all authors prospectively register their systematic reviews, the real extent of duplication may differ from that in PROSPERO, particularly because those who knowingly duplicate without good reason may be less likely to register their review.

Owing to time and resource constraints our research only examined four of the seventeen research areas related to COVID-19. Although it is not our intention to repeat this research for the remaining research categories, this could be an interesting area for future research, particularly to see whether duplication remains consistently apparent in more recent areas of research related to COVID-19 for example, long-covid.

As records can be created in the PROSPERO system prior to submission, it is possible that similar systematic reviews could be registered during this period. If authors did not re-search PROSPERO prior to submission, unintended duplication could occur. However, this data is not available in the PROSPERO system so was not captured in this study.

We observed low quality registration forms and a substantial number of systematic reviews that lacked external funding; however, a comparison was not made to non-duplicate reviews. Further work addressing this could be of interest.

For peer review only - http://bmjopen.bmj.com/site/about/guidelines.xhtml

Finally, while the surveys provide a useful insight into the reasons for replication of previous systematic reviews, the number of responses received was small (41 reponces /138 contacted). This may not be representative of all PROSPERO users, as those who responded could feel that they had a legitimate reason to replicate, whilst those who did not may not have responded to the survey when asked.

Conclusions

This research has highlighted that registration of duplicate systematic reviews related to COVID-19 has occurred frequently in PROSPERO, with 138 review teams addressing research questions of existing reviews in the first year of the pandemic. It adds to the evidence highlighting the problem of duplication and research waste in reviews and evidence syntheses and has identified a need to increase awareness of the need to avoid unintended duplication among those conducting reviews. PROSPERO, which has provided registration free of charge and served the systematic review community for ten years, responded to the COVID-19 pandemic by ensuring that reviews relating to COVID-19 were prioritised and registered/published rapidly. Changes made to accommodate this, along with associated reflection on the role of registration, has informed thinking about future developments and consideration of how PROSPERO can continue to support the global systematic review community over the next ten years and be part of the research infrastructure that stands ready to respond effectively to future global health challenges.

Contributorship Statement

The corresponding author attests that all listed authors meet authorship criteria and that no others meeting the criteria have been omitted.

Lucy Beresford and Ruth Walker equally contributed to the data curation, formal analysis, investigation, visualisation and writing of the original draft, Lesley Stewart provided support for these tasks. Lesley Stewart led the conceptualisation, project administration, validation and reviewing and editing of the drafts, Lucy Beresford and Ruth Walker provided support for these tasks. All authors contributed equally to the methodology used in the research. Lesley Stewart provided overall project supervision.

Funding Statement

This study/project is funded by the NIHR Technology Assessment Reviews Programme (14/25/13). The views expressed are those of the author(s) and not necessarily those of the NIHR or the Department of Health and Social Care.

For peer review only - http://bmjopen.bmj.com/site/about/guidelines.xhtml

This report presents independent research commissioned by the National Institute for Health Research (NIHR). The views and opinions expressed by authors in this publication are those of the authors and do not necessarily reflect those of the NHS, the NIHR, MRC, CCF, NETSCC, Health Technology Assessment programme or the Department of Health.

Competing Interests

Production of PROSPERO is funded by the NIHR through an overarching research contract 14/25/13. Supplementary funding was made through this award to support PROSPERO's response to COVID-19 which included this project. This research funding is made to the University of York. Lesley Stewart is the principal investigator and award holder. Ruth Walker is partially funded on this grant and is part of the PROSPERO team. Lucy Beresford was funded by the supplementary Covid funding for a period of six months to provide additional support to PROSPERO and contribute to this project.

Data Sharing Statement

Data from the extracted duplicated studies have been provided in Appendix D. Data from the survey can be obtained upon request.

Ethics

The study was granted ethical approval from the University of York, Health Sciences Research Governance Committee. The study was deemed to be very low risk so was dealt with as a Chair's Action. The letter can be provided upon request.

Acknowledgements

We would like to acknowledge Gordon Dooley who extracted the data from PROSPERO for analysis.

References

1. Lorenc T KC, Raine G, Shemilt I, Sutcliffe K, D'Souza P, Burchett H, Hinds K, Macdowall W, Melton H, Richardson M, South E, Stansfield C, Thomas S, Kwan I, Wright K, Sowden A, Thomas J (2020) COVID. COVID-19: living map of the evidence. London: EPPI-Centre, Social Science Research Unit, UCL Social Research Institute, University College London.; 2020.
2. Page MJ, Welch VA, Haddaway NR, Karunananthan S, Maxwell LJ, Tugwell P. "One more time": why replicating some syntheses of evidence relevant to COVID-19 makes sense. *J Clin Epidemiol* 2020; **125**: 179-82.
3. Page MJ, McKenzie JE, Bossuyt PM, et al. The PRISMA 2020 statement: an updated guideline for reporting systematic reviews. *Bmj* 2021; **372**.
4. Booth A, Clarke M, Ghersi D, Moher D, Petticrew M, Stewart L. An international registry of systematic-review protocols. *Lancet* 2011; **377**(9760): 108-9.

For peer review only - http://bmjopen.bmj.com/site/about/guidelines.xhtml

5. Moher D, Booth A, Stewart L. How to reduce unnecessary duplication: use PROSPERO. *Bjog* 2014; **121**(7): 784-6.

6. Stewart L, Moher D, Shekelle P. Why prospective registration of systematic reviews makes sense. *Systematic Reviews* 2012; **1**(1): 7.

7. Pieper D, Rombey T. Where to prospectively register a systematic review. *Systematic Reviews* 2022; **11**(1): 8.

8. Glasziou PP, Sanders S, Hoffmann T. Waste in covid-19 research. *Bmj* 2020; **369**: m1847.

9. Siontis KC, Ioannidis JPA. Replication, Duplication, and Waste in a Quarter Million Systematic Reviews and Meta-Analyses. *Circ Cardiovasc Qual Outcomes* 2018; **11**(12): e005212.

10. Chalmers I, Glasziou P. Avoidable waste in the production and reporting of research evidence. *Lancet* 2009; **374**(9683): 86-9.

11. Tricco AC, Antony J, Zarin W, et al. A scoping review of rapid review methods. *BMC Medicine* 2015; **13**(1): 224.

12. McDonald S, Turner S, Page MJ, Turner T. Most published systematic reviews of remdesivir for COVID-19 were redundant and lacked currency. *Journal of clinical epidemiology* 2022; **146**: 22-31.

13. Smith J, Noble H. Reviewing the literature. *Evidence Based Nursing* 2016; **19**(1): 2.

14. Helliwell JA, Thompson J, Smart N, Jayne DG, Chapman SJ. Duplication and nonregistration of COVID-19 systematic reviews: Bibliometric review. *Health Science Reports* 2022; **5**(3): e541.

15. Tugwell P, Welch VA, Karunananthan S, et al. When to replicate systematic reviews of interventions: consensus checklist. *BMJ* 2020; **370**: m2864.

Figure Legends

- **Figure 1.** Number of COVID-19 related registrations between March 2020 to July 2021.
- **Figure 2.** Flow chart showing number of records screened and identified as ‘duplicates’ in the four COVID-19 research areas.
- **Figure 3.** Number of ‘duplicate’ reviews registered in PROSPERO between March 2020 and January 2021, by research area.
- **Figure 4.** The country in which the survey respondent’s review is/was being carried out.

For peer review only

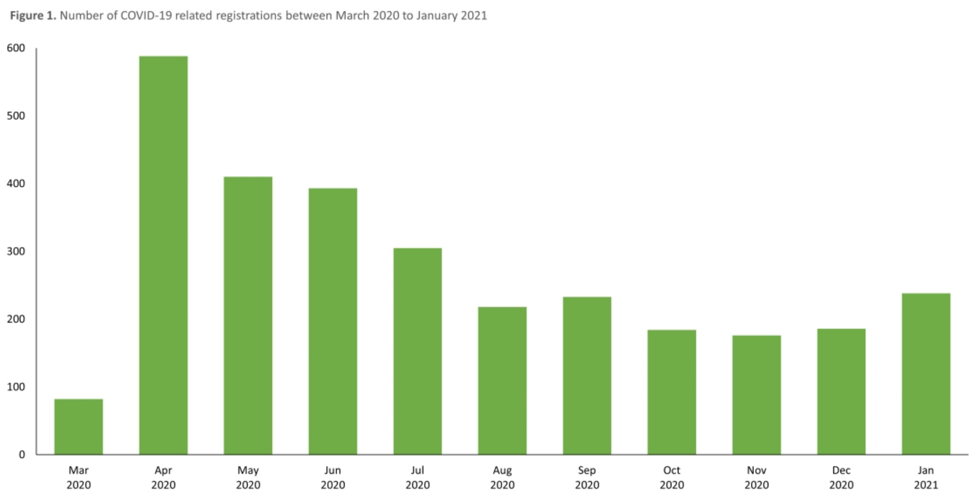

Figure 1. Number of COVID-19 related registrations between March 2020 to January 2021

817x428mm (130 x 130 DPI)

Figure 2. *Flow chart showing number of records screened and identified as 'duplicates' in the four COVID-19 research areas.*

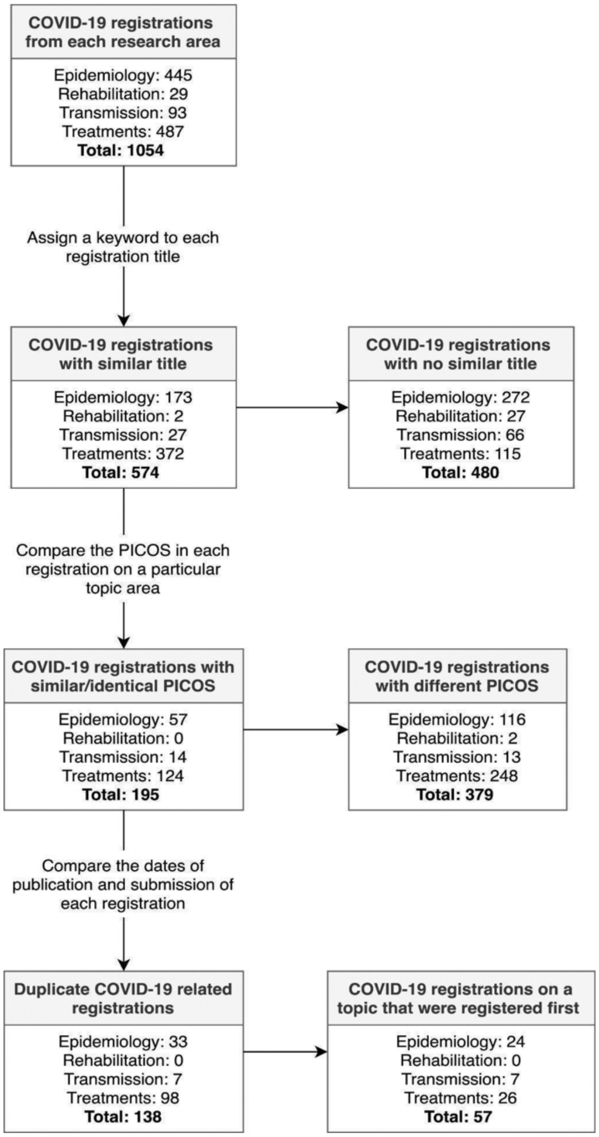

287x582mm (130 x 130 DPI)

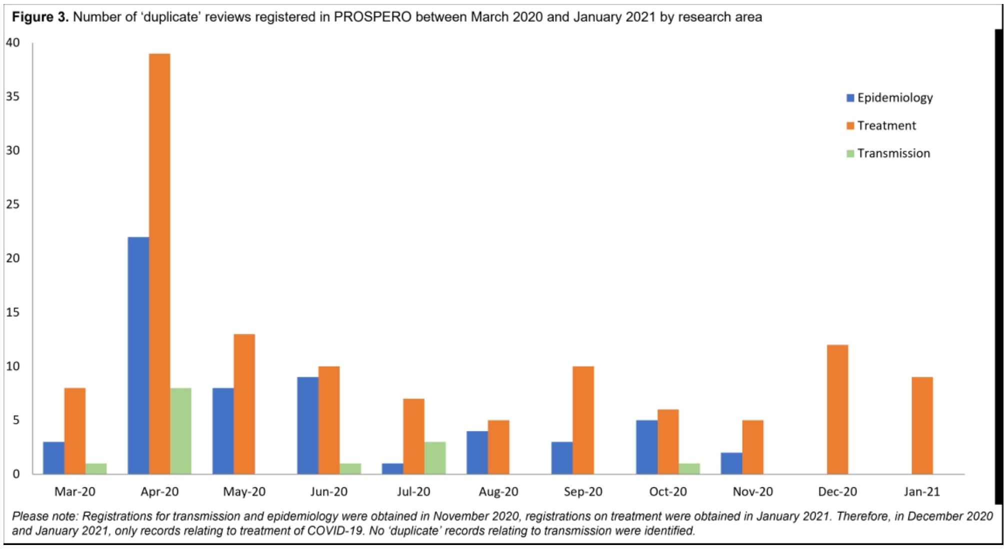

Figure 3. Number of 'duplicate' reviews registered in PROSPERO between March 2020 and January 2021 by research area

784x423mm (130 x 130 DPI)

Figure 4. The country in which the survey respondent's review is being carried out

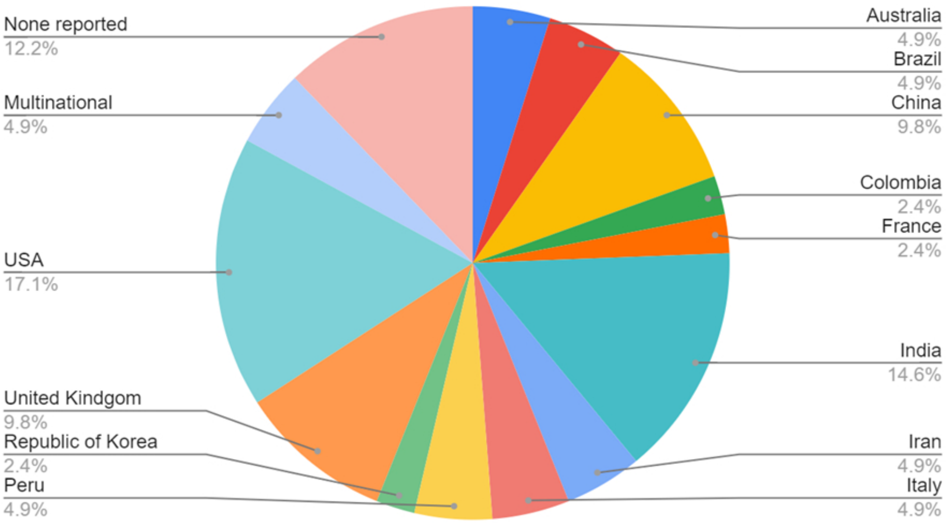

622x385mm (38 x 38 DPI)

Appendix A

Search Terms:
'(((coronavirus or corona-virus) AND (wuhan or beijing or shanghai or Italy or South-Korea or korea or China or Chinese or 2019-nCoV or nCoV or COVID-19 or Covid19 or SARS-CoV* or SARSCov2 or ncov)) OR (pneumonia AND Wuhan) or "COVID-19" or "2019-nCoV" or "SARS-CoV" or SARSCOV2 or 2019-nCov or "2019 coronavirus" or "2019 corona virus" or covid19 or ncov OR "novel corona virus" or "new corona virus" or "nouveau corona virus" or "2019 corona virus" OR "novel coronavirus" or "new coronavirus" or "nouveau coronavirus" or "2019 coronavirus" or "Long Covid") NOT Animal:DB'

For peer review only

For peer review only

Appendix B

Data Extraction

Table 1. *Data extraction template for identifying duplicate reviews*

Record number	Research area	Date submitted	Date registered	Is the PICOS similar/identical to existing review?	Answer to screening question? *	Is the review funded?	Have authors acknowledged similar reviews?	Authors reason for duplication?	Is there a reason for duplication from our perspective?

**Screening questions before authors submit a PROSPERO registration ask whether their review is not similar to an existing review, sufficiently different, or similar to an existing review, but repetition is needed*

For peer review only

Appendix C

COVID-19 Systematic Review Duplication in PROSPERO Questionnaire

The survey can be entirely anonymous. But it would be useful for us to be able to link your responses to the PROSPERO record, so you can choose to include your personal information. If you do provide your identifying ID number, this will not be included or used in any future publications and it will not affect the status of your PROSPERO registration.

This survey has been granted ethical approval. If you choose to include your PROSPERO registration number, your data will be handled in compliance with GDPR and the UK Data Protection Act (2018). For more information, please visit the links on the University of York's policy on Data Protection Legislation, GDPR compliant research and the general privacy notice.

Q1 What is your PROSPERO registration number

____

About your COVID-19 related systematic review

Q2 Have you conducted a systematic review before?

- Yes (1)
- No (2)
- Other (3) ____

Q3 What research area was your systematic review looking at?

- Epidemiology (1)
- Transmission (2)
- Treatments (3)
- Other (4) ____

Q4 Why did you conduct your systematic review on a COVID-19 related topic?

- To inform future research that you had planned (such as a primary research study) (1)
- To inform funders/policy makers (2)
- For personal/departmental interest (3)
- Other (4) ____

Q5 Please provide details of the country in which the review is being carried out. For multi-national collaborations, give details of all the countries involved.

____

For peer review only

Q6 Is your review funded

- Yes (1)
- No (2)
- Other (3) ____

Display This Question:

If Is your review funded != No

Q7 If your review is funded, please provide details of the individuals, organizations, groups or other legal entities who take responsibility for initiating, managing, sponsoring and/or financing the review

____

Q8 Do you intend to publish or disseminate the results of your systematic review?

- Yes (1)
- No (2)
- Maybe (3)
- Other (4) ____

Display This Question:

If Do you intend to publish or disseminate the results of your systematic review? = No

Q9 Why did you decide not to publish the results of your systematic review?

____

Q10 Is there any other information that you wish to provide about your systematic review?

____

The following questions were specific to the registrant's response to the triage questions at PROSPERO submission

For peer review only

Not Similar Sub-Questions

When registering, users are prompted to search PROSPERO and check whether a similar systematic review has been registered. Some duplication, intended with good reason (e.g. the existing one is out of date or used suboptimal methods), is justifiable. When registering, you indicated that your review was not similar to an existing review

Q12 To help us understand this further, please indicate which statement applies most to your situation.

- I searched PROSPERO prior to registration and identified some/all of the similar records (1)
- I searched PROSPERO prior to registration but did not identify the similar records (2)
- I did not search PROSPERO prior to registration (3)

Display This Question:

If To help us understand this further, please indicate which statement applies most to your situation. = I searched PROSPERO prior to registration and identified some/all of the similar records

Q13 Please could you tell us more about the reasons you conducted a review similar to those which you identified?

____

Display This Question:

If To help us understand this further, please indicate which statement applies most to your situation. = I searched PROSPERO prior to registration but did not identify the similar records

Q14 Please could you tell us more about how you searched PROSPERO, including the search terms you used. This could help us to improve our guidance about how to search PROSPERO in the future.

____

Display This Question:

If To help us understand this further, please indicate which statement applies most to your situation. = I did not search PROSPERO prior to registration

Q15 Please could you tell us more about the reasons you did not search PROSPERO before registration?

____

For peer review only

Sufficiently Different Sub-Questions

When registering, users are prompted to search PROSPERO and check whether a similar systematic review has been registered. Some duplication, intended with good reason (e.g. the existing one is out of date or used suboptimal methods), is justifiable. When registering, you indicated that your review was similar to an existing review but sufficiently different to justify this review.

Q12 To help us understand this further, please indicate which statement applies most to your situation.

- I searched PROSPERO and identified some/ all of the existing reviews that were similar to my own. (1)
- Other (2)

Display This Question:

If To help us understand this further, please indicate which statement applies most to your situation. = I searched PROSPERO and identified some/ all of the existing reviews that were similar to my own.

Q13 For each of the similar reviews you identified, please could you tell us more about the reasons why your review is sufficiently different.

____

Display This Question:

If To help us understand this further, please indicate which statement applies most to your situation. = Other

Q14 Please could you tell us whether you searched PROSPERO, and if you did, please could you explain more about how you searched PROSPERO, including the search terms you used. This could help us to improve our guidance about how to search PROSPERO in the future.

____

For peer review only

Repetition Needed Sub-Questions

When registering, users are prompted to search PROSPERO and check whether a similar systematic review has been registered. Some duplication, intended with good reason (e.g. the existing one is out of date or used suboptimal methods), is justifiable. When registering, you indicated that your review was similar to an existing review but repetition was needed.

Q12 To help us understand this further, please indicate which statement applies most to your situation.

- I searched PROSPERO and identified some/ all of the existing reviews that were similar to my own. (1)
- Other (2)

Display This Question:

If To help us understand this further, please indicate which statement applies most to your situation. = I searched PROSPERO and identified some/ all of the existing reviews that were similar to my own.

Q13 For each of the similar reviews you identified, please could you tell us more about the reasons why repetition was needed.

____

Display This Question:

If To help us understand this further, please indicate which statement applies most to your situation. = Other

Q14 Please could you tell us whether you searched PROSPERO, and if you did, please could you explain more about how you searched PROSPERO, including the search terms you used. This could help us to improve our guidance about how to search PROSPERO in the future.

____

For peer review only

Appendix D

Research area	Date submitted	Date registered	Answer to screening question?	Have authors acknowledged similar reviews?	Authors reason for duplication?	Is there reason for duplication from our perspective?	Is the review funded?
**Cancer**	17/04/20	21/04/20	-	-	-	-	None
**Cancer**	22/04/20	22/04/20	NS	No	No	Perhaps, submitted one day after last was published	None
**Cardiovascular Disease**	14/04/20	20/04/20	-	-	-	-	None
**Cardiovascular Disease**	31/05/20	01/06/20	NS	No	No	No	National Natural Science Foundation of China
**Children**	15/04/20	21/04/20	-	-	-	-	None
**Children**	18/04/20	20/04/20	-	-	-	-	Nanchang University

For peer review only - http://bmjopen.bmj.com/site/about/guidelines.xhtml

Research area	Date submitted	Date registered	Answer to screening question?	Have authors acknowledged similar reviews?	Authors reason for duplication?	Is there reason for duplication from our perspective?	Is the review funded?
**Children**	02/08/20	06/08/20	RN*	No	-	Potentially. Four months since previous systematic review	None
**Comorbidities: Impact on Mortality**	24/03/20	03/04/20	-	-	-	-	None
**Comorbidities: Impact on Mortality**	22/05/20	28/05/20		No	None	No	None
**Comorbidities: Impact on Mortality**	29/10/20	30/10/20	NS*	No	None	Perhaps- to update evidence (5 months after last review).	None
**Comorbidities: Prevalence**	27/03/20	06/04/20	-	-	-	-	Human Resources Development Operational Programme Grant
**Comorbidities: Prevalence**	26/04/20	28/04/20	NS*	No	No	No	None

Research area	Date submitted	Date registered	Answer to screening question?	Have authors acknowledged similar reviews?	Authors reason for duplication?	Is there reason for duplication from our perspective?	Is the review funded?
**Comorbidities: Severe Outcomes and Death**	26/02/20	17/03/20	-	-	-	-	National Key R&D Program of China.
**Comorbidities: Severe Outcomes and Death**	26/03/20	16/04/20	SD	Yes	Yes: no risk of bias in CRD42020170315	Yes, CRD42020170315 do not report methods of quality assessment).	None
**Comorbidities: Severe Outcomes and Death**	31/03/20	30/04/20	NS	No	None	No	None
**Comorbidities: Severe Outcomes and Death**	13/04/20	16/04/20	NS	No	None	No	Second Affiliated Hospital of Nanchang University
**COVID-19 in Healthcare Workers**	06/04/20	15/04/20	--	-	-	-	None
**COVID-19 in Healthcare Workers**	27/04/20	28/04/20	NS	None	No	No	None
**Diabetes**	28/04/20	04/05/20	-	-	-	-	None

For peer review only

Research area	Date submitted	Date registered	Answer to screening question?	Have authors acknowledged similar reviews?	Authors reason for duplication?	Is there reason for duplication from our perspective?	Is the review funded?
**Diabetes**	30/06/20	30/06/20	NS	No	None	No	Natural Science Foundation of Tianjan and Tianjan Education Committee
**Diabetes**	05/10/20	06/10/20	NS	No	None	Perhaps update of review potentially needed	None
**Echocardiographic Findings**	18/08/20	19/08/20	-	-	-	-	None
**Echocardiographic Findings**	19/09/20	22/09/20	NS	No	No	No	None
**Face Masks**	29/09/20	29/09/20	-	-	-	-	None
**Face Masks**	19/10/20	28/10/20	SD	No	No	Yes. Living systematic review	None
**General Risk Factors: Impact on Mortality**	26/03/20	03/04/20	-	-	-	-	None

Research area	Date submitted	Date registered	Answer to screening question?	Have authors acknowledged similar reviews?	Authors reason for duplication?	Is there reason for duplication from our perspective?	Is the review funded?
**General Risk Factors: Impact on Mortality**	04/04/20	09/04/20	NS	None	No	Potentially- only one day after previous record was registered.	Wellcome Trust
**General Risk Factors: Impact on Poor Outcomes and Mortality**	27/02/20	03/04/20	-	-	-	-	National Natural Science Foundation
**General Risk Factors: Impact on Poor Outcomes and Mortality**	15/03/20	27/03/20	-	-	-	-	None
**General Risk Factors: Impact on Poor Outcomes and Mortality**	18/03/20	23/03/20	NS	None	No	No	Chinese Natural Science Foundation and the Training Program of Shanghai Health and Family Planning System
**General Risk Factors: Impact on Poor Outcomes and Mortality**	28/03/20	09/04/20	SD	Yes	Yes, provide additional analysis (regression)	Yes	None

Research area	Date submitted	Date registered	Answer to screening question?	Have authors acknowledged similar reviews?	Authors reason for duplication?	Is there reason for duplication from our perspective?	Is the review funded?
**General Risk Factors: Impact on Poor Outcomes and Mortality**	12/04/20	16/04/20	NS	No	None	No	None
**General Risk Factors: Impact on Poor Outcomes and Mortality**	14/04/20	17/05/20	NS*	No	None	None	None
**General Risk Factors: Impact on Poor Outcomes and Mortality**	20/04/20	21/04/20	RN	No	None	None	None
**General Risk Factors: Impact on Poor Outcomes and Mortality**	06/05/20	06/05/20	SD	No	None	No	Department of Epidemiology, College of Public Health, University of Nebraska Medical
**General Risk Factors: Impact on Poor Outcomes and Mortality**	11/05/20	12/05/20	NS	No	None	No	None
**Long COVID**	15/10/20	23/10/20	-	-	-	-	None

Research area	Date submitted	Date registered	Answer to screening question?	Have authors acknowledged similar reviews?	Authors reason for duplication?	Is there reason for duplication from our perspective?	Is the review funded?
**Long COVID**	31/10/20	02/11/20	SD	No	No	No	None
**Manifestations: Taste and Smell**	22/05/20	29/05/20	-	-	-	-	None
**Manifestations: Taste and Smell**	20/06/20	25/06/20	NS	No	None	None	Health Commission of Hunan Province and Human Pharmaceutical Association
**Mental Health in Healthcare Workers**	31/07/20	03/08/20	-	-	-	-	National Council for Scientific and Technological Development - CNPq, Brazilian Government
**Mental Health in Healthcare Workers**	19/10/20	22/10/20	NULL	No	No	No	None
**Neurological Manifestations**	22/05/20	01/06/20	-	-	-	-	None
**Neurological Manifestations**	02/06/20	02/06/20	NS	No	None	Perhaps, submitted one day after previous was published	None

For peer review only - http://bmjopen.bmj.com/site/about/guidelines.xhtml

Research area	Date submitted	Date registered	Answer to screening question?	Have authors acknowledged similar reviews?	Authors reason for duplication?	Is there reason for duplication from our perspective?	Is the review funded?
**Obesity**	13/04/20	06/04/20	-	-	-	-	NIHR Applied Research and Care
**Obesity**	29/04/20	01/05/20	NS	None	None	No	University of Indonesia
**Obesity**	13/05/20	15/05/20	NS	No	None	None	Programme Széchenyi 20
**Obesity**	10/11/20	12/11/20	SD	No	No	Perhaps update of review potentially needed	None
**Pregnancy**	04/04/20	23/06/20	-	-	-	-	Partly funded by WHO
**Pregnancy**	13/04/20	17/04/20	-	-	-	-	None
**Pregnancy**	24/04/20	24/04/20	NS*	No	None	No	None
**Skin Manifestations**	23/06/20	25/06/20	-	-	-	-	None

Research area	Date submitted	Date registered	Answer to screening question?	Have authors acknowledged similar reviews?	Authors reason for duplication?	Is there reason for duplication from our perspective?	Is the review funded?
**Skin Manifestations**	28/06/20	29/06/20	SD	Yes	Yes (none will be published before completion)	No	None
**Treatment: ACE Inhibitors and ARBs**	07/06/20	19/06/20	-	-	-	-	Major Project of National Science and Technology on New Drug etc.
**Treatment: ACE Inhibitors and ARBs**	03/07/20	08/07/20	SD	No	None	No	None
**Treatment: Metformin**	21/08/20	25/08/20	-	-	-	-	ARC-EM
**Treatment: Metformin**	12/09/20	15/09/20	RN	None	None	No	National Natural Science China
**Breastfeeding**	06/04/20	21/04/20	SD	References CRD420173886	Yes. But review is sufficiently so no duplication occurred.	Yes	None
**Breastfeeding**	11/04/20	20/04/20	N/A	-	-	-	None
**Breastfeeding**	17/07/20	20/07/20	SD	Not explicitly	No	No	None

For peer review only

Research area	Date submitted	Date registered	Answer to screening question?	Have authors acknowledged similar reviews?	Authors reason for duplication?	Is there reason for duplication from our perspective?	Is the review funded?
**Breastfeeding**		10/10/20	NS	No	No	No comparator.	None
**Maternal to Fetal Transmission**	16/03/20	24/03/20	NS	-	-	-	N/A
**Maternal to Fetal Transmission**	22/06/20	03/07/20	SD	No	No	No	None
**Surgery**	02/04/20	22/04/20	NS	-	-	-	None
**Surgery**	25/04/20	28/04/20	NS	No	N/A	No	None
**Viral Shedding**	24/04/20	29/04/20	NS	-	-	-	None
**Viral Shedding**	22/06/20	22/06/20	NS*	Yes, acknowledge CRD420181914	Yes, previous registration deemed poor quality	Potentially- poor reporting in previous registration. Authors have given reason for duplication.	None
**Weather**	28/03/20	07/04/20	NS	-	-	-	None

Research area	Date submitted	Date registered	Answer to screening question?	Have authors acknowledged similar reviews?	Authors reason for duplication?	Is there reason for duplication from our perspective?	Is the review funded?
**Weather**	01/04/20	08/04/20	NS	-	-	-	Federal University of Pará, Brazil.
**Weather**	27/04/20	30/04/20	NS	No	No	Yes, looking at seasons and times of year rather than climate conditions.	None
**Weather**	03/07/20	03/07/20	SD	No	No	Almost identical. No real reason for duplication.	None
**Ace inhibitors**	05/04/20	14/04/20	NS	-	-	-	Self funded
**Ace inhibitors**	13/04/20	17/04/20	NS*	-	-	-	None
**Ace inhibitors**	03/05/20	05/05/20	NS	No	NO	NO	None

For peer review only - http://bmjopen.bmj.com/site/about/guidelines.xhtml

Research area	Date submitted	Date registered	Answer to screening question?	Have authors acknowledged similar reviews?	Authors reason for duplication?	Is there reason for duplication from our perspective?	Is the review funded?
**Ace inhibitors**	04-Mayx-20	04/05/20	NS	No	NO	NO	China Postdoctoral Science Foundation (2019M652593) and Henan Postdoctoral Science Foundation (1902006)
**Ace inhibitors**	08/05/20	11/05/20	SD	Partially	NO	NO	None
**Ace inhibitors**	13/05/20	14/05/20	NS	No	NO	NO	This study was supported by National Natural Science Foundation of China, 81970203; National Natural Science Foundation of China, 81570212; National Natural Science Foundation of China, 31800976 Chongqing Science and Health Joint Medical Research Project, 2018QNXM024
**Ace inhibitors**	27/07/20	28/07/20	NS	No	NO	NO	Qingsong Qin is sponsored by Department of education of Guangdong province, China

Research area	Date submitted	Date registered	Answer to screening question?	Have authors acknowledged similar reviews?	Authors reason for duplication?	Is there reason for duplication from our perspective?	Is the review funded?
**Ace inhibitors**	03/08/20	04/08/20	SD	Partially	NO	NO	Disciplines Construction Project of PUMC (Grant number: 20192102). Chinese Academy of Medical Sciences, CAMS Innovation Fund for Medical Sciences (Grant number: 2018-I2M-1-002)
**All treatment living**	20/04/20	22/04/20	SD	-	-	-	None
**All treatment living**	27/04/20	28/04/20	SD	Partially	NO	No	This review received some funding from the Agence Nationale de la Recherche (ANR) and from the WHO
**All treatment MA**	07-Feb-20	31/03/20	NS	-	-	-	NA

For peer review only

Research area	Date submitted	Date registered	Answer to screening question?	Have authors acknowledged similar reviews?	Authors reason for duplication?	Is there reason for duplication from our perspective?	Is the review funded?
**All treatment MA**	09/03/20	20/03/20	NS	No	No	No	This study will be funded by Research Funds of Shanghai Health and Family Planning commission (20184Y0022, 20194Y0007), Cultivation fund of clinical research of Renji hospital (PY2018-III-06), Clinical Pharmacy Innovation Research Institute of Shanghai Jiao Tong University School of Medicine (CXYJY2019ZD001, CXYJY2019QN004).
**All treatment MA**	22/03/20	24/03/20	NS	No	No	No	Shubham Misra is a recipient of DST-INSPIRE PhD Fellowship from Department of Science & Technology, Government of India
**All treatment MA**	07/04/20	17/04/20	NS	No	No	No	https://covid19.kacst.edu.sa/grants/

Research area	Date submitted	Date registered	Answer to screening question?	Have authors acknowledged similar reviews?	Authors reason for duplication?	Is there reason for duplication from our perspective?	Is the review funded?
**All treatment MA**	20/04/20	22/04/20	SD	Partially	No	No	None
**All treatment MA**	05/05/20	06/05/20	NS	No	No	No	None
**All treatment MA**	13/10/20	16/10/20	NS	No	No	No	This work was funded by the Guidance Plan for Social Development of Taizhou Municipal Science and Technology (ssf20160141).
**All treatment MA**	14/11/20	07/12/20	NS	No	No	No	None
**All treatment MA**	05/01/21	08/01/21	Not in system	No	No	No	None
**All treatment NMA**	07/04/20	15/04/20	NS	NO	Yes		
**All treatment NMA**	12/04/20	16/04/20	SD	Partially	NA		
**All treatment NMA**	18/04/20	22/04/20	SD	Partially	No	No	The research project will be assisted with the by Griffith University

Research area	Date submitted	Date registered	Answer to screening question?	Have authors acknowledged similar reviews?	Authors reason for duplication?	Is there reason for duplication from our perspective?	Is the review funded?
							Postgraduate Research Scholarship
**All treatment NMA**	19/04/20	20/04/20	NS	NO	No	No	None
**All treatment NMA**	23/04/20	23/04/20	NS	NO	No	No	Supported by the Programme of Introducing Talents of Discipline to Universities, China, the 111 Project, D18011)
**All treatment NMA**	27/04/20	27/04/20	SD	Partially	No	No	None
**All treatment NMA**	15/05/20	19/05/20	NS	NO	No	No	None
**All treatment NMA**	18/06/20	24/06/20	NS	NO	No	No	None
**All treatment NMA**	18/10/20	26/10/20	NS	NO	No	No	None
**All treatment umbrella**	13/05/20	20/05/20	NS	NA	NA	NA	NA

Research area	Date submitted	Date registered	Answer to screening question?	Have authors acknowledged similar reviews?	Authors reason for duplication?	Is there reason for duplication from our perspective?	Is the review funded?
**All treatment umbrella**	07/09/20	07/09/20	NS	No	No	No	Indian Council of Medical Research
**All treatment umbrella**	04/10/20	05/10/20	NS	No	No	No	None
**Anticoagulant**	02/06/20	02/06/20	NS	-	-	-	
**Anticoagulant**	02/07/20	03/07/20	SD	Partially	No	No	None
**Anticoagulant**	14/10/20	15/10/20	NS	No	No	No	None
**Anticoagulant**	07/01/21	11/01/21	not in list	No	No	No	None
**Blood plasma**	12/03/20	24/03/20	NS	-	-	-	None
**Blood plasma**	28/03/20	08/04/20	SD	Partially		No reasons why sufficiently different.	Funded by the UK Forum
**Blood plasma**	02/04/20	02/04/20	NS	No	No	No	None

Research area	Date submitted	Date registered	Answer to screening question?	Have authors acknowledged similar reviews?	Authors reason for duplication?	Is there reason for duplication from our perspective?	Is the review funded?
**Blood plasma**	06/04/20	15/04/20	SD	Partially	No	No reasons why sufficiently different.	None
**Blood plasma**	10/04/20	15/04/20	NS	No	No	No	Education Foundation of Chengdu University of Traditional Chinese Medic
**Blood plasma**	17/06/20	19/06/20	SD	Partially	No	No	None
**Blood plasma**	05/07/20	06/07/20	SD	Partially	No	No reasons why sufficently different.	None
**Blood plasma**	16/07/20	16/07/20	NS	No	No	No	None
**Blood plasma**	12/08/20	14/08/20	SD	Partially	No	No reasons why sufficently different.	None
**Blood plasma**	09/12/20	22/12/20	not in list	No	No	No	None
**Blood plasma**	16/12/20	17/12/20	not in list		No	No	None

Research area	Date submitted	Date registered	Answer to screening question?	Have authors acknowledged similar reviews?	Authors reason for duplication?	Is there reason for duplication from our perspective?	Is the review funded?
**Blood plasma**	21/12/20	22/12/20	not in list	No	Potentially severe patient pop.	No	None
**Colchicine**	17/09/20	21/09/20					None
**Colchicine**	11/01/21	12/01/21	not in list	No	No	No	None
**Corticosteroids**	03/03/20	20/04/20	NS	-	-	-	None
**Corticosteroids**	20/04/20	21/04/20	NS	-	-	-	None
**Corticosteroids**	28/04/20	30/04/20	NS	No	No	No	None
**Corticosteroids**	06/05/20	06/05/20	SD	Partially	No	No	This work was supported by National Key Research and Development Project (20YFC2005700), Key Realm R&D Program of Guangdong Province (2019B030335001), and Medical Scientific Research

Research area	Date submitted	Date registered	Answer to screening question?	Have authors acknowledged similar reviews?	Authors reason for duplication?	Is there reason for duplication from our perspective?	Is the review funded?
							Foundation of Guangdong Province of China (A2019407)
**Corticosteroids**	07/07/20	07/07/20	SD	Partially	No	No	None
**Corticosteroids**	23/09/20	23/09/20	SD	Partially	No	No	None
**Corticosteroids**	07/12/20	08/12/20	not in list	No	No	No	None
**Corticosteroids**	21/12/20	22/12/20	not in list	No	No	No	NaFoUniMedCovid19“ (FKZ: 01KX21) „CEO-Sys“
**Corticosteroids**	05/01/21	12/01/21	not in list	No	No	No	None
**ECMO**	02/04/20	14/04/20		-	-	-	None

Research area	Date submitted	Date registered	Answer to screening question?	Have authors acknowledged similar reviews?	Authors reason for duplication?	Is there reason for duplication from our perspective?	Is the review funded?
**ECMO**	07/04/20	14/04/20		-	-	-	None
**ECMO**	16/04/20	17/04/20	NS	No	No	No	None
**ECMO**	03/05/20	04/05/20	SD	Partially	No	No	None
**ECMO**	15/06/20	18/06/20	NS	No	No	No	None
**ECMO**	19/06/20	22/06/20	NS	No	No	No	None
**ECMO**	17/09/20	21/09/20	NS	No	No	No	None
**ECMO**	22/11/20	23/11/20	NS	No	No	No	This work was supported by the research Grant 20YFC0841300 from Ministry of Science and Technology of the People's Republic of China.
**ECMO**	21/12/20	22/12/20	NS	No	No	No	None

Research area	Date submitted	Date registered	Answer to screening question?	Have authors acknowledged similar reviews?	Authors reason for duplication?	Is there reason for duplication from our perspective?	Is the review funded?
**ECMO**	04/01/21	07/01/21	NS	No	No	No	None
**Hydroxychloroquine**	22-Feb-20	17/03/20		-	-	-	None
**Hydroxychloroquine**	29/03/20	16/04/20	NS	No	No	No	Major Projects of Science and Technology Innovation 2025 in Ningbo, China
**Hydroxychloroquine**	04/04/20	06/04/20	SD	Partially	No	No	NIHR Applied Research Collaboration East Midlands (ARC EM), UK
**Hydroxychloroquine**	06/04/20	07/04/20	SD	Partially	No	No	None
**Hydroxychloroquine**	06/04/20	09/04/20	NS	No	No	No	None
**Hydroxychloroquine**	07/04/20	17/04/20	NS	No	No	No	None
**Hydroxychloroquine**	10/04/20	21/04/20	SD	Partially	No	No	None
**Hydroxychloroquine**	14/04/20	17/04/20	SD	Partially	No	No	None

Research area	Date submitted	Date registered	Answer to screening question?	Have authors acknowledged similar reviews?	Authors reason for duplication?	Is there reason for duplication from our perspective?	Is the review funded?
**Hydroxychloroquine**	20/04/20	21/04/20	SD	Partially	No	No	None
**Hydroxychloroquine**	27/04/20	28/04/20	NS	No	No	No	None
**Hydroxychloroquine**	22/05/20	22/05/20	SD	Partially	No	No	None
**Hydroxychloroquine**	10/08/20	12/08/20	NS	No	No	No	None
**Hydroxychloroquine**	19/08/20	19/08/20	NS	No	No	No	None
**Hydroxychloroquine**	12/09/20	17/09/20	NS	No	No	No	None
**Hydroxychloroquine**	12/09/20	15/09/20	SD	Partially	No	No	None
**Interferon**	26/03/20	26/03/20	NA	-	-	-	None
**Interferon**		07/04/20	NA	-	-	-	None
**Interferon**	17/11/20	20/11/20	SD	Partially	No	No	None

Research area	Date submitted	Date registered	Answer to screening question?	Have authors acknowledged similar reviews?	Authors reason for duplication?	Is there reason for duplication from our perspective?	Is the review funded?
**Interferon**	21/11/20	03/12/20	NS	No	No	No	None
**Ivermectin**	08/07/20	09/07/20		-	-	-	-
**Ivermectin**	20/10/20	09/11/20	NS	no	No	No	None
**Ivermectin**	24/12/20	07/01/21	NS	no	No	No	The Institute for Health Technology Assessment (IETS),
**Ivermectin**	11/01/21	12/01/21	NS	no	No	No	None
**JAK inhibitors**	07/06/20	09/06/20		-	-	-	NA
**JAK inhibitors**	04/01/21	12/01/21	SD	partially	no	no	None
**lopinavir**	06/04/20	15/04/20	NA	NA	NA	NA	NA
**lopinavir**	09/04/20	15/04/20	NA	NA	NA	NA	NA

Research area	Date submitted	Date registered	Answer to screening question?	Have authors acknowledged similar reviews?	Authors reason for duplication?	Is there reason for duplication from our perspective?	Is the review funded?
**lopinavir**	12/04/20	16/04/20	NA	NA	NA	NA	NA
**lopinavir**	17/04/20	21/04/20	NS	no	no	no	None
**lopinavir**	24/04/20	24/04/20	NS	no	no	no	None
**lopinavir**	13/01/21	15/01/21	SD	partially	no	no	This study is carried out at the request of the Ministry of Health and Social Protection and the Disaster Risk Management Fund. Contract No. 9677-MECOVID19-1009-20.
**Prone position**	24/06/20	24/06/20	NA	-	-	-	-
**Prone position**	28/08/20	01/09/20	NS	no	no	no	None
**Prone position**	11/09/20	14/09/20	NS	no	no	no	None
**Prone position**	19/10/20	22/10/20	NS	no	no	no	None

Research area	Date submitted	Date registered	Answer to screening question?	Have authors acknowledged similar reviews?	Authors reason for duplication?	Is there reason for duplication from our perspective?	Is the review funded?
**Prone position**	16/11/20	20/11/20	RN	yes	no	no	None
**Prone position**	10/12/20	11/12/20	NS	no	no	no	None
**Prone position**	12/12/20	14/12/20	NS	no	no	no	None
**RAAS**	07/05/20	11/05/20		-	-	-	None
**RAAS**	14/06/20	17/06/20	NS	no	no	no	None
**RAAS**	15/09/20	16/09/20	RN	yes	no	no	None
**Remdisivir**	08-Feb-20	23/03/20	NS	-	-	-	National Natural Science Foundation of China
**Remdisivir**	02/04/20	14/04/20	NS	No	No	No	CDT-Africa, College of Health Sciences, Addis Ababa University
**Remdisivir**	21/04/20	23/04/20	NS	No	No	No	None
**Remdisivir**	30/04/20	01/05/20	SD	No	No	No	None

Research area	Date submitted	Date registered	Answer to screening question?	Have authors acknowledged similar reviews?	Authors reason for duplication?	Is there reason for duplication from our perspective?	Is the review funded?
**Remdisivir**	26/05/20	26/05/20	NS	No	No	No	Tongji Hospital, Tongji Medical College
**Remdisivir**	20/09/20	21/09/20	NS	No	No	No	None
**Remdisivir**	12/10/20	15/10/20	NS	No	No	No	Zhejiang Medicine and Health Science and Technology Project; Natural Science Foundation of Zhejiang Province
**Tocilizumab**	22/03/20	24/03/20	NS	-	-	-	None
**Tocilizumab**	18/06/20	19/06/20	NS		No	No	None
**Tocilizumab**	21/06/20	22/06/20	SD		No	No	None
**Tocilizumab**	26/06/20	01/07/20	NS		No	No	Nicola Luigi Bragazzi and Jianhong Wu are partially supported by the Canadian Institute of Health Research

Research area	Date submitted	Date registered	Answer to screening question?	Have authors acknowledged similar reviews?	Authors reason for duplication?	Is there reason for duplication from our perspective?	Is the review funded?
							(CIHR) 2019 Novel Coronavirus (COVID-19) rapid research program
**Tocilizumab**	14/08/20	17/08/20	NS		No	No	None
**Tocilizumab**	06/11/20	10/11/20	NS	no	No	No	None
**Tocilizumab**	11/12/20	18/12/20	NS	no	No	No	1.3.5 project for disciplines of excellence, West China Hospital, Sichuan University
**Tocilizumab**	16/12/20	17/12/20	SD	Partially	No	No	None

* Registrant changed their answer to the screening question. Grey cells represent 'duplicate' records, white cells represent records registered first.
Abbreviations: NS, Not Similar; RN, Repetition Needed; SD, Sufficiently Different.

Appendix E

Duplicate Registrations Characteristics

Table 2: Number of ‘duplicate’ records registered in each COVID-19 related topic area.

**Topic**	**N duplicates**
**Epidemiology**	
**Health Condition and Impact on Outcome**	
Cancer	1
Cardiovascular disease	1
Diabetes	2
General Risk Factors	8
Obesity	3
Comorbidities	6
**Healthcare Workers**	
COVID-19 in healthcare workers	1
Mental Health in Healthcare Workers	1
**Clinical presentation of COVID-19**	
Echocardiographic Findings	1
Manifestations (Dermatological)	1
Manifestations (Taste and Smell)	1
Manifestations (Neurological)	1
Long-COVID	1
**Transmission of COVID-19**	
Facemasks	1
Pregnancy and mother-baby transmission	1
**Treatment of COVID-19**	
ACE-inhibitors	1
Metformin	1
**Total number of duplicates**	**31**
**Treatments**	
**All treatments**	
All treatment living	1
All treatment MA	7
All treatment umbrella	2
All treatment NMA	8
**Pharmacological interventions**	
ACE-inhibitors	6
Anticoagulants	1
Corticosteroids	4
Hydroxychloroquine	14
Interferon	2

Ivermectin	3
JAK inhibitors	1
**Topic**	**N duplicates**
**Pharmacological interventions**	
lopinavir	3
RAAS	2
Remdisivir	6
Tocilizumab	7
**Non-pharmacological interventions**	
Blood plasma	8
ECMO	5
Prone position	6
**Total number of duplicates**	**86**
**Transmission**	
Mother-baby transmission	2
Transmission during Surgery	1
Weather	1
**Total number of duplicates**	**4**

For peer review only

For peer review only

BMJ Open

The extent and nature of duplication in PROSPERO using COVID-19 related registrations: a retrospective investigation and survey

Journal:	*BMJ Open*
Manuscript ID	bmjopen-2022-061862.R2
Article Type:	Original research
Date Submitted by the Author:	07-Nov-2022
Complete List of Authors:	Beresford, Lucy; University of York, Centre for Review and Dissemination<br>Walker, Ruth; University of York Centre for Reviews and Dissemination<br>Stewart, Lesley; University of York Centre for Reviews and Dissemination
**Primary Subject Heading**:	Research methods
Secondary Subject Heading:	Infectious diseases
Keywords:	COVID-19, QUALITATIVE RESEARCH, STATISTICS & RESEARCH METHODS

SCHOLARONE™
Manuscripts

I, the Submitting Author has the right to grant and does grant on behalf of all authors of the Work (as defined in the below author licence), an exclusive licence and/or a non-exclusive licence for contributions from authors who are: i) UK Crown employees; ii) where BMJ has agreed a CC-BY licence shall apply, and/or iii) in accordance with the terms applicable for US Federal Government officers or employees acting as part of their official duties; on a worldwide, perpetual, irrevocable, royalty-free basis to BMJ Publishing Group Ltd ("BMJ") its licensees and where the relevant Journal is co-owned by BMJ to the co-owners of the Journal, to publish the Work in this journal and any other BMJ products and to exploit all rights, as set out in our licence.

The Submitting Author accepts and understands that any supply made under these terms is made by BMJ to the Submitting Author unless you are acting as an employee on behalf of your employer or a postgraduate student of an affiliated institution which is paying any applicable article publishing charge ("APC") for Open Access articles. Where the Submitting Author wishes to make the Work available on an Open Access basis (and intends to pay the relevant APC), the terms of reuse of such Open Access shall be governed by a Creative Commons licence – details of these licences and which Creative Commons licence will apply to this Work are set out in our licence referred to above.

Other than as permitted in any relevant BMJ Author's Self Archiving Policies, I confirm this Work has not been accepted for publication elsewhere, is not being considered for publication elsewhere and does not duplicate material already published. I confirm all authors consent to publication of this Work and authorise the granting of this licence.

For peer review only

Manuscript Title: **The extent and nature of duplication in PROSPERO using COVID-19 related registrations: a retrospective investigation and survey**

Authors:

Miss Lucy Beresford, Research Fellow in Evidence Synthesis[1]
lucy.beresford@york.ac.uk*

Ruth Walker, Research Fellow in Evidence Synthesis[1]
ruth.walker@york.ac.uk*

Professor Lesley Stewart, Professor of Evidence Synthesis[1]
lesley.stewart@york.ac.uk

[1] Centre for Reviews and Dissemination, University of York
* These authors contributed equally to the work

Contact information for corresponding author

Ruth Walker, Research Fellow in Evidence Synthesis
Centre for Reviews and Dissemination, University of York
ruth.walker@york.ac.uk

For peer review only

Abstract

Objectives

During COVID-19, PROSPERO (an international prospective register of systematic reviews) experienced a surge in registrations for COVID-19 related systematic reviews, and duplication of research questions became apparent. Duplication can waste funding, time and research effort and make policy-making more difficult.

This project explored the extent of and reasons for duplication of COVID-19 related systematic review registrations in PROSPERO during the pandemic.

Design

Retrospective analysis of COVID-19 related registrations in PROSPERO, and a qualitative survey.

Setting

PROSPERO was searched for registrations related to four COVID-19 research areas: epidemiology, rehabilitation, transmission, and treatments.

Methods

Records identified were compared using PICOS (Population, Intervention, Comparator, Study Design) elements of PROSPERO registration forms. Registrations with similar or identical PICOS were evaluated further as 'duplicates'.

Authors of 'duplicate' registrations were invited to complete a survey asking whether they searched PROSPERO prior to registration, identified similar reviews, and if so, why they continued with their review.

Results

1054 COVID-19 reviews were registered between March 2020 and January 2021, of which 138 were submitted when at least one similar protocol was already registered in PROSPERO. Duplication was greatest in reviews of COVID-19 treatments; for example, there were 14 similar reviews evaluating the efficacy of hydroxychloroquine.

From 138 authors invited to take part in the survey, we received 41 responses. Most respondents said that they identified similar reviews when they searched PROSPERO prior to registration. Main

For peer review only

reasons given for ‘duplication’ were differences in PICOS or planned analyses (N = 13), poor quality of previous registrations (N = 2), and the need to update evidence (N = 3).

Conclusions

This research highlights that registration of similar and duplicate systematic reviews related to COVID-19 in PROSPERO occurred frequently. Awareness of research waste is required, and initial checking for similar reviews should be embedded within good review practice.

Strengths and Limitations

- As this research project was conducted by members of the PROSPERO team, we were able to access data that are not publicly available – specifically whether authors identified other similar reviews prior to registration. Therefore, our work provides a unique insight into the nature of duplication in PROSPERO.
- The inclusion of a survey provided further insight into the nature of duplication in PROSPERO, which has allowed us to offer specific improvements for protocol registration in PROSPERO, specifically at a time where cooperation and collaboration between researchers was pertinent given the public health crisis
- We received a relatively low response to our survey (30%). Therefore, while it provides useful insights into the reasons for duplication, it may not be representative of all those who registered ‘duplicate’ reviews.
- Our study was limited by available resource and were only able to explore the extent and nature of duplication from March 2020 until January 2021. Therefore, key milestones in the pandemic (such as the global role out of vaccinations, or the availability of antivirals) that could have impacted the extent of duplication were not explored.
- For the same reason we were only able to evaluate the extent and nature of duplication in four of the seventeen COVID-19 related topic areas. Although we attempted to choose a broad range of topics, the extent of duplication could differ in other COVID-19 research areas

For peer review only - http://bmjopen.bmj.com/site/about/guidelines.xhtml

Introduction

During the COVID-19 pandemic, a significant volume of medical research has been conducted to better understand the virus, its biology and associated disease.[1] Large numbers of systematic reviews have synthesised the emerging evidence.[2] It is best practice to register systematic reviews at the protocol stage to help avoid unplanned duplication and to enable comparison between methods reported in the final publication and that which was planned in the protocol.[3] This can be done in registries such as PROSPERO, an international prospective register of systematic review protocols, funded by the National Institute of Health and Care Research (NIHR) in the UK. PROSPERO was launched in 2011 with the intention of increasing transparency, limiting unintended duplication, and reducing the risk of bias in systematic reviews.[4-6]

In the ten years since its launch in 2011, prospective registration of systematic reviews has become routine and the importance of this, acknowledged widely. At launch, PROSPERO anticipated registration of around 2000 systematic reviews per year. In 2020 40,639 reviews were registered, and PROSPERO now includes over 125,000 registration records. PROSPERO has global reach, with registrations originating from 194 countries and territories in 2020. In March 2020, PROSPERO prioritised registration of review protocols related to COVID-19. Our intention was to support the global pandemic research endeavour by making information about planned and ongoing reviews public as quickly as possible and providing researchers and policy makers with an overview of the evidence synthesis pipeline. This would also help reduce unintended duplication, by enabling authors to check whether similar reviews already existed before embarking on a new one, based on up-to-date information (COVID-19 registrations are published within 24 hours of submission Monday- Friday). Submissions are registered in PROSPERO through the process described in Box 1.

To aid identification of reviews on topics of key relevance to pandemic research, a single click search by broad COVID-19 research categories was implemented on the PROSPERO home page. The research categories have been regularly updated as new topics emerge. PROSPERO encourages authors not to duplicate existing systematic reviews but recognises that duplication may be intended with good reason (e.g., the existing review is out of date or used suboptimal methods). As such, when registering, users are asked to indicate whether their review is (i) not similar to an existing review, (ii) sufficiently different, or (iii) similar to an existing review, but repetition is needed, as part of a series of eligibility questions before completing the registration form.

For peer review only - http://bmjopen.bmj.com/site/about/guidelines.xhtml

Box 1.

- Registration in PROSPERO involves the submission and publication of key information about the design and conduct of a systematic review
- Authors should register their systematic review once they have designed their review protocol but before they begin data extraction
- Prior to submission authors answer a series of 'triage questions' to check whether their review is eligible for inclusion.
- Previously, applications were checked by the PROSPERO team to ensure that they were in scope and that the required data had been provided. Since March 2020, to enable rapid processing of COVID-19 registrations, some applications have been published automatically following simple automated checks. No quality assessment or peer review is involved. Records are published on an open access electronic database.
- Registration information can be amended by authors should plans change. These changes are published, and a date stamped audit trail of previous versions made available in the public record.
- Registration records are permanent, and authors are asked to provide links to subsequent reports and publications.
- PROSPERO assigns each registered review a unique registration number. This number should be cited in publications.
- Peer reviewers and readers can compare what was reported for the review with what was intended in the review protocol.

Despite encouragement to check PROSPERO for similar systematic reviews and to not duplicate without good reason, PROSPERO administrators quickly raised the issue that multiple systematic reviews related to COVID-19, addressing the same research question, were being registered. Unnecessary duplication of research is wasteful and creates additional work for healthcare providers who need to make sense of evidence in a rapidly evolving field.[7-9] Duplication can also cause confusion, if findings between reviews are inconsistent.[2]

Using retrospective analyses, we aimed to explore the extent of duplication within PROSPERO registration records related to COVID-19, including whether the extent differed between rapid, full and living systematic reviews and/or particular areas of COVID-19 related research. We also aimed to explore the nature of duplication, including whether this was done unknowingly, covertly or for good reason by authors who were open and transparent about replication. Finally, we aimed to better understand the reasons for repetition from the authors' perspective and explore who funded duplicate reviews by conducting a survey.

Methods

Searches

PROSPERO was searched for registrations relating to COVID-19 from March 2020 to January 2021. PROSPERO created a search strategy for users to identify COVID-19 related records, which was used in this research project (see Appendix A). When registering their review, authors chose their 'type

For peer review only - http://bmjopen.bmj.com/site/about/guidelines.xhtml

and method of review' from a list. These tags then acted as filters on the PROSPERO website to identify the eligible studies. Due to resource constraints, not all research areas could be assessed. Four of the seventeen COVID-19 research categories were selected for further study: epidemiology, transmission, treatment, and rehabilitation. These research categories were chosen to capture a wide range of different topic areas addressing questions relating to COVID-19. For practical reasons, the records were downloaded for reviews of transmission, rehabilitation and epidemiology in November 2020, and treatment in January 2021.

Identification of duplicate records

Within each topic area, records were each assigned a keyword based on the review title. Those with the same keyword were compared, firstly using the title of the registration record and then for those with similar titles, using the Population, Intervention/Exposure, Comparator, Outcome and Study design (PICOS) criteria recorded in the PROSPERO registration form. Those addressing the same research question, based on the PICOS criteria were retained (see Figure 2). To ensure that decisions were being made consistently, this was conducted in duplicate by two reviewers for a subset of records (the rehabilitation and transmission reviews, and a random sample of epidemiology registrations (100 from 445 records)). Discrepancies in decisions were discussed between reviewers to reach a consensus. The remaining records were screened singularly.

For reviews addressing the same research question, the date of first submission to PROSPERO and the date of publication were obtained. Registrations submitted after the publication of a record with a similar or identical PICOS, were deemed 'duplicates' and retained. Reviews that have been submitted to PROSPERO but not yet registered/published on the public website (for example if the record was returned to authors for additional information or clarification) are not visible on the PROSPERO COVID-19 search. Therefore, if a record was *submitted* before another record on the same topic and *published* after it, this was not counted as duplication because the authors would not have had knowledge of the other review at the point they submitted their registration form.

Data extraction

For duplicate reviews, data on whether authors acknowledged similar existing registrations in PROSPERO when answering the screening questions prior to registration were recorded. From the public registration forms, data concerning the nature of duplication were extracted (see Appendix B, for Data Extraction template). This included whether the authors provided reasons for conducting a new review and whether they had an external funding source. Furthermore, we extracted

For peer review only - http://bmjopen.bmj.com/site/about/guidelines.xhtml

information about whether reviews were rapid, full, or living systematic reviews. These topics were defined based on whether the PROSPERO record included the wording 'living' or 'rapid' in their record, if not they were deemed to be 'full' systematic reviews. This information was used to assess whether full systematic reviews were conducted after rapid reviews for particular research topics, and/or whether rapid or full systematic reviews were being registered after living systematic reviews.

Surveys

To further understand the reasons why people duplicated existing reviews, authors of duplicate registrations were contacted using the email address of the named contact, provided in the PROSPERO form, and invited to complete a short questionnaire. Authors were asked whether they searched PROSPERO prior to registration, and whether they identified any similar reviews. Authors were also asked why they believed their review to be sufficiently different from an existing review, or why they felt repetition was needed if they had identified a similar review. The contacted authors had up to six weeks to respond to the survey, and reminders to complete the questionnaire were sent one week before it closed. A copy of the questionnaire is given in Appendix C.

Patient Public Involvement

Patients or the public were not involved in the design, conduct or reporting, or dissemination plans of our research. Our findings from this research were discussed with the COVID Evidence Network to support Decision making (COVID-END) community.

Results

Search results

From 01 March 2020 to 31st of January 2021, PROSPERO registered 3,013 protocols related to COVID-19. The monthly number of registrations published on PROSPERO peaked in April 2020 with 588 protocols (Figure 1). Overall, 1054 registrations related to the four COVID-19 topic areas of interest (until January 2021).

Descriptive characteristics

574 records were found to have a similar review title and had their full PROSPERO record examined. 195 of these records had a similar or identical PICO(S) (57 of which were the first instance of reviews addressing particular research questions). The remaining 138 duplicate records had been submitted when at least one protocol with similar or identical PICOS had already been published in PROSPERO.

For peer review only - http://bmjopen.bmj.com/site/about/guidelines.xhtml

Figure 2 presents the number of records at each stage of the screening process. The data from the extracted duplicate studies are provided in Appendix D.

There were 33 (7.4% of those registered) duplicate reviews for epidemiological topics, 98 (20%) for treatments and seven (7.5%) relating to the transmission of COVID-19. No duplicate reviews were identified in the rehabilitation topic area. Figure 3 shows the number of duplicated reviews registered by month between March 2020 and January 2021 in each research area.

The number of duplicate reviews on specific questions ranged from one to 14. Multiple duplicate registrations were most common in reviews evaluating COVID-19 treatments, including reviews evaluating the efficacy of drugs such as hydroxychloroquine (14 duplicate reviews), tocilizumab (seven duplicate reviews), remdesivir (six duplicate reviews), the use of blood plasma (seven duplicate reviews) and prone position (six duplicate reviews). The majority of epidemiological reviews only had a single duplicate review, except for those evaluating the impact of comorbidities (six duplicate reviews) and reviews of general risk factors in patients with COVID-19 (eight duplicate reviews). The number of duplicates per research question are given in the Appendix E,.

Screening questions

When answering the PROSPERO screening questions prior to registering their protocol, authors of 85 of the 138 records considered as duplicate reviews did not acknowledge existing similar reviews in PROSPERO and selected 'Not Similar' on the screening question. Forty-eight indicated that their review was sufficiently different to those already registered and five authors indicated that repetition of existing reviews was needed.

Review type

Table 1 shows the number of registrations published on PROSPERO by research area and review type. We identified 138 duplicate full systematic reviews, only two of which were replacing rapid reviews that addressed the same research question.

Table 1. Number of Registered and Duplicate Reviews in four COVID-19 topic areas by type of systematic review.

Review Type	Number of Registered Reviews				
	Epidemiology	Rehabilitation	Treatment	Transmission	Total
**Number of Registered Reviews**					
**Rapid**	16[a]	2	21[b]	7	**46**
**Living**	12	3	27	7	**49**

For peer review only - http://bmjopen.bmj.com/site/about/guidelines.xhtml

**Full**	417	24	439	79	**520**
**Total**	**445**	**29**	**487**	**93**	**615**
**Number of Duplicate Reviews**					
**Rapid**	1	0	2	0	**3**
**Living**	1	0	6	1	**8**
**Full**	31	0	90	6	**127**
**Total**	**33**	**0**	**98**	**7**	**138**

[a]Two reviews were rapid, living systematic reviews and are counted in the 'living' row. [b]One review was a rapid, living systematic review and is counted in the 'living' row.

It was often difficult to differentiate between rapid and full systematic reviews as many of the registration forms were poorly completed. Some reviews also indicated they would complete within 4-6 weeks but did not identify as being a rapid review or detail abbreviated or rapid review methods, such as use of text-mining during the screening process.

Twenty-three full systematic reviews and one living systematic review were found to be registered after living systematic reviews addressing the same research question.

Reasons for duplication

Provided in registrations

For each duplicate record, we explored whether the authors had acknowledged previous registrations in their PROSPERO record, and if so, gave reasons for duplication. Thirty-three (24%) authors of duplicate records did so. Only 4 of these authors (12%) provided a reason for replication within their record despite being advised during the registration process that the reasons for duplication should be made clear.

Provided in response to survey

We received 41 unique responses to the survey investigating reasons for duplication, from 12 countries (Figure 4). Eighteen responses (21% of those contacted) were from authors who during PROSPERO screening indicated that their review was 'not similar' to existing registered reviews, 21 responses, (45% of those contacted) indicated their review was 'sufficiently different' and two responses (40% of those contacted) indicated 'repetition was needed'.

Of the respondents that provided their registration number in the survey, 68% had registered reviews relating to treatments for COVID-19. Overall, 90% of respondents had conducted a systematic review previously, and 92.5% intended to publish the results of their review. Most respondents had received no specific funding for their systematic review (87.5%).

For peer review only - http://bmjopen.bmj.com/site/about/guidelines.xhtml

Of the 18 respondents who indicated their review was ‘not similar’ to existing registered reviews, eight responded in the survey that they searched PROSPERO and did not identify any reviews on the same topic area. When asked to provide search terms used to do this, some were so broad that a large number of results would have been returned (such as single words like ‘COVID-19’). Other respondents used multiple search terms; when we repeated these searches, all identified the duplicate records related to their review. Ten respondents said they searched PROSPERO and found some reviews on the same topics, but decided their review was different to the existing reviews found by their search.

All 21 respondents who indicated in the screening questions that their reviews were sufficiently different to other registered reviews responded that their searches identified some or all the reviews that we identified as being similar to their own. However, no respondent provided justification to why they believed their review was significantly different to previous registrations in their PROSPERO registration.

Of the two respondents who indicated that repetition was needed, neither provided their justification for repeating previous systematic reviews.

The reasons given for conducting similar reviews are summarised in Table 2.

Table 2. *Summary of reasons for duplications from respondents of the questionnaire*

**Response to Screening Question**	**Responses in Survey**
**Not Similar**	• Different objectives or planned analyses<br>• Poor quality of previous systematic reviews<br>• Duplication was needed owing to the rapid rate that new studies on COVID-19 were being published
**Sufficiently Different**	• Differences in PICOS, study design or planned analyses<br>• Differences in databases searched<br>• Poor quality of previous systematic reviews<br>• Duplication was needed owing to the rapid rate that new studies on COVID-19 were being published
**Repetition Needed**	• No systematic review was published or completed at the time of registration<br>• Repetition was needed to confirm/refute previous systematic review conclusions*

For peer review only - http://bmjopen.bmj.com/site/about/guidelines.xhtml

Discussion

To synthesise the considerable amount of primary research conducted during the COVID-19 pandemic[7], a substantial number of systematic reviews have been produced, many of which have been prospectively registered on PROSPERO. There is growing concern that these include duplicate systematic reviews that have replicated without good reason. Unintended or unnecessary duplication of research wastes research funding, time, and effort and creates extra work for policy makers and healthcare providers who need to determine what novel information, if any, new reviews provide.[2 5 10]

We aimed to assess the extent and nature of duplication within PROSPERO registration records related to COVID-19, within four research areas: epidemiology, rehabilitation, transmission, and treatment. Duplication was found to be extensive (accounting for 13% of records across these research areas). Researchers may have instead made a more valuable contribution to knowledge by considering alternative review topics (as advised on the PROSPERO homepage).

The nature of this duplication was first examined by assessing whether duplication was a result of rapid reviews (a form of evidence synthesis in which some systematic review methods are condensed or omitted to produce results in a timely manner) being replaced by full systematic reviews.[11] As rapid reviews provide a useful overview of the available research but may stop short of providing a full or detailed synthesis, a later full systematic review may be justified. However, only two examples of this were identified in our sample. We also identified rapid and full systematic reviews registered after living systematic reviews on the same topic. For example, following the registration of a living systematic review and network meta-analysis on all interventions for COVID-19, an additional seven duplicate standard systematic reviews were registered. This is particularly wasteful as living reviews update regularly to capture and synthesise new research, preventing the need for many systematic reviews assessing the same research question at different points in time. Living reviews are particularly important in situations, such as the COVID-19 pandemic, where the research evolves rapidly and published standard systematic reviews may become out of date quickly.[12]

Responses to our survey provided more insight into the reasons for duplication. Notably, users indicated that they did not identify any similar records when they searched PROSPERO prior to registration, despite our own simple repetition of their searches identifying similar reviews. This may indicate poor use of search terms that are not able or likely to identify similar protocols, or lack of

For peer review only - http://bmjopen.bmj.com/site/about/guidelines.xhtml

examination of the reviews returned by their search (the very broad terms that some authors reported using would have returned a large number of potentially similar reviews that could have made screening a daunting task). To tackle this, PROSPERO may need to develop better tools and guidance to support more effective searching and continue to promote research integrity, such as making explanation of planned replication a mandatory requirement.

Whilst some valid reasons for duplication of systematic reviews were provided in the survey, for example, difference in the PICOS criteria and poor quality of previous reviews, these explanations were not included in the public PROSPERO registration forms, and therefore this detail can not inform decisions in future research for example, umbrella reviews that to synthesise outcome data from these systematic reviews.

Given the time and resources required to conduct systematic reviews, a surprising number of duplicate reviews lacked external funding. Unfunded reviews may have been initiated due to an altruistic desire to do something to help, and at least early in the pandemic, many funders were not set up to make rapid funding decisions. The number of reviews lacking external funding may, however, be concerning as, at least in the case in high income countries, these reviewers may not have been subject to the same level scrutiny or review as afforded by the funding application process (e.g., demonstrating sufficient experience and a track record in conducting systematic reviews). Research funders may also require due diligence in checking for existing reviews. Research that assesses whether funded proposals translate into better quality research, compared to self-funded proposals could be useful.

Although not formally assessed here, a general observation from this research was the low quality of registration forms we reviewed. Poorly completed forms make it difficult, even for experienced systematic reviewers, to determine whether an existing review is similar or identical to their own and may therefore be contributing to unnecessary duplication of systematic reviews. Fewer high quality systematic reviews are always more valuable to decision makers than many of poor-quality ones, which have the potential to damage the confidence of the research community in systematic reviews, which are viewed as the 'gold standard' of evidence-based research.[13 14]

Journals may play a role in preventing systematic reviews that have been unnecessarily duplicated from being published. However, whilst some journals now require PROSPERO registration as a prerequisite for publication, peer reviewers or editors may not have the resource, nor deem it necessary to search PROSPERO for similar reviews to ascertain whether there is a need for a new systematic review. Issues around the low quality of registration forms, as previously discussed, may

For peer review only - http://bmjopen.bmj.com/site/about/guidelines.xhtml

For peer review only

also impede the ability to do this and inform the editorial decisions. Further research may involve conducting a search for published reviews arising from the duplicated protocols identified in this research, to better understand how many make it to publication.

PROSPERO could help tackle the concerns raised in this research by engaging in outreach activity to improve the quality of registration records and by enhancing/promoting the guidance provided on the PROSPERO website. Further developments aimed at reducing the level of duplication could involve the provision of resources to help users decide whether duplication is necessary or useful, such as the checklist created by Tugwell et al (2021).[15] This could include information on the purpose of living systematic reviews and why duplication might be unnecessary after a research question has been addressed with this methodology. This advice would be especially helpful for researchers who lack experience in systematic reviews and may not understand the importance of avoiding duplication. An improved functionality around discovery of similar reviews could be introduced, including the development of automation that would highlight potentially similar reviews that exist in the database. If similar reviews are identified, additional information from the author could be requested, including the purpose and justification for duplication and the added value of their systematic review. More generally, PROSPERO's educational role could be developed, not only in promoting best review methods and practice, but also in highlighting the importance of avoiding unintended duplication and research waste. With 175,140 signed up users (setting up a PROSPERO account is prerequisite to registering a review) we are well placed to reach a large audience and help build expertise and infrastructure that would better serve decision making in a future pandemic.

Limitations

As not all authors prospectively register their systematic reviews, the real extent of duplication may differ from that in PROSPERO, particularly because those who knowingly duplicate without good reason may be less likely to register their review.

Owing to time and resource constraints our research only examined four of the seventeen research areas related to COVID-19. Although it is not our intention to repeat this research for the remaining research categories, this could be an interesting area for future research, particularly to see whether duplication remains consistently apparent in more recent areas of research related to COVID-19 for example, long-covid.

As records can be created in the PROSPERO system prior to submission, it is possible that similar systematic reviews could be registered during this period. If authors did not re-search PROSPERO

For peer review only - http://bmjopen.bmj.com/site/about/guidelines.xhtml

prior to submission, unintended duplication could occur. However, this data is not available in the PROSPERO system so was not captured in this study.

We observed low quality registration forms and a substantial number of systematic reviews that lacked external funding; however, a comparison was not made to non-duplicate reviews. Further work addressing this could be of interest.

Finally, while the surveys provide a useful insight into the reasons for replication of previous systematic reviews, the number of responses received was small (41 reponces /138 contacted). This may not be representative of all PROSPERO users, as those who responded could feel that they had a legitimate reason to replicate, whilst those who did not may not have responded to the survey when asked.

Conclusions

This research has highlighted that registration of duplicate systematic reviews related to COVID-19 has occurred frequently in PROSPERO, with 138 review teams addressing research questions of existing reviews in the first year of the pandemic. It adds to the evidence highlighting the problem of duplication and research waste in reviews and evidence syntheses and has identified a need to increase awareness of the need to avoid unintended duplication among those conducting reviews. PROSPERO, which has provided registration free of charge and served the systematic review community for ten years, responded to the COVID-19 pandemic by ensuring that reviews relating to COVID-19 were prioritised and registered/published rapidly. Changes made to accommodate this, along with associated reflection on the role of registration, has informed thinking about future developments and consideration of how PROSPERO can continue to support the global systematic review community over the next ten years and be part of the research infrastructure that stands ready to respond effectively to future global health challenges.

Contributorship Statement

The corresponding author attests that all listed authors meet authorship criteria and that no others meeting the criteria have been omitted.

Lucy Beresford and Ruth Walker equally contributed to the data curation, formal analysis, investigation, visualisation and writing of the original draft, Lesley Stewart provided support for these tasks. Lesley Stewart led the conceptualisation, project administration, validation and reviewing and editing of the drafts, Lucy Beresford and Ruth Walker provided support for these

For peer review only - http://bmjopen.bmj.com/site/about/guidelines.xhtml

tasks. All authors contributed equally to the methodology used in the research. Lesley Stewart provided overall project supervision.

Funding Statement

This study/project is funded by the NIHR Technology Assessment Reviews Programme (14/25/13). The views expressed are those of the author(s) and not necessarily those of the NIHR or the Department of Health and Social Care.

This report presents independent research commissioned by the National Institute for Health Research (NIHR). The views and opinions expressed by authors in this publication are those of the authors and do not necessarily reflect those of the NHS, the NIHR, MRC, CCF, NETSCC, Health Technology Assessment programme or the Department of Health.

Competing Interests

Production of PROSPERO is funded by the NIHR through an overarching research contract 14/25/13. Supplementary funding was made through this award to support PROSPERO's response to COVID-19 which included this project. This research funding is made to the University of York. Lesley Stewart is the principal investigator and award holder. Ruth Walker is partially funded on this grant and is part of the PROSPERO team. Lucy Beresford was funded by the supplementary Covid funding for a period of six months to provide additional support to PROSPERO and contribute to this project.

Data Sharing Statement

Data from the extracted duplicated studies have been provided in Appendix D. Data from the survey can be obtained upon request.

Ethics

The study was granted ethical approval from the University of York, Health Sciences Research Governance Committee. The study was deemed to be very low risk so was dealt with as a Chair's Action. The letter can be provided upon request.

Acknowledgements

We would like to acknowledge Gordon Dooley who extracted the data from PROSPERO for analysis.

For peer review only - http://bmjopen.bmj.com/site/about/guidelines.xhtml

References

1. Lorenc T KC, Raine G, Shemilt I, Sutcliffe K, D'Souza P, Burchett H, Hinds K, Macdowall W, Melton H, Richardson M, South E, Stansfield C, Thomas S, Kwan I, Wright K, Sowden A, Thomas J (2020) COVID. COVID-19: living map of the evidence. London: EPPI-Centre, Social Science Research Unit, UCL Social Research Institute, University College London., 2020.
2. Page MJ, Welch VA, Haddaway NR, et al. "One more time": why replicating some syntheses of evidence relevant to COVID-19 makes sense. *J Clin Epidemiol* 2020;125:179-82. doi: 10.1016/j.jclinepi.2020.05.024 [published Online First: 2020/05/29]
3. Page MJ, McKenzie JE, Bossuyt PM, et al. The PRISMA 2020 statement: an updated guideline for reporting systematic reviews. *Bmj* 2021;372
4. Booth A, Clarke M, Ghersi D, et al. An international registry of systematic-review protocols. *Lancet* 2011;377(9760):108-09. doi: 10.1016/s0140-6736(10)60903-8
5. Moher D, Booth A, Stewart L. How to reduce unnecessary duplication: use PROSPERO. *Bjog* 2014;121(7):784-6. doi: 10.1111/1471-0528.12657 [published Online First: 2014/03/19]
6. Stewart L, Moher D, Shekelle P. Why prospective registration of systematic reviews makes sense. *Systematic Reviews* 2012;1(1):7. doi: 10.1186/2046-4053-1-7
7. Glasziou PP, Sanders S, Hoffmann T. Waste in covid-19 research. *Bmj* 2020;369:m1847. doi: 10.1136/bmj.m1847 [published Online First: 2020/05/14]
8. Pieper D, Rombey T. Where to prospectively register a systematic review. *Systematic Reviews* 2022;11(1):8. doi: 10.1186/s13643-021-01877-1
9. Siontis KC, Ioannidis JPA. Replication, Duplication, and Waste in a Quarter Million Systematic Reviews and Meta-Analyses. *Circ Cardiovasc Qual Outcomes* 2018;11(12):e005212. doi: 10.1161/circoutcomes.118.005212 [published Online First: 2018/12/19]
10. Chalmers I, Glasziou P. Avoidable waste in the production and reporting of research evidence. *Lancet* 2009;374(9683):86-9. doi: 10.1016/s0140-6736(09)60329-9 [published Online First: 2009/06/16]
11. Tricco AC, Antony J, Zarin W, et al. A scoping review of rapid review methods. *BMC Medicine* 2015;13(1):224. doi: 10.1186/s12916-015-0465-6
12. McDonald S, Turner S, Page MJ, et al. Most published systematic reviews of remdesivir for COVID-19 were redundant and lacked currency. *Journal of clinical epidemiology* 2022;146:22-31.
13. Helliwell JA, Thompson J, Smart N, et al. Duplication and nonregistration of COVID-19 systematic reviews: Bibliometric review. *Health Science Reports* 2022;5(3):e541. doi: https://doi.org/10.1002/hsr2.541
14. Smith J, Noble H. Reviewing the literature. *Evidence Based Nursing* 2016;19(1):2. doi: 10.1136/eb-2015-102252
15. Tugwell P, Welch VA, Karunananthan S, et al. When to replicate systematic reviews of interventions: consensus checklist. *BMJ* 2020;370:m2864. doi: 10.1136/bmj.m2864

Figure Legends

- **Figure 1.** Number of COVID-19 related registrations between March 2020 to July 2021.
- **Figure 2.** Flow chart showing number of records screened and identified as 'duplicates' in the four COVID-19 research areas.
- **Figure 3.** Number of 'duplicate' reviews registered in PROSPERO between March 2020 and January 2021, by research area.
- **Figure 4.** The country in which the survey respondent's review is/was being carried out.

For peer review only - http://bmjopen.bmj.com/site/about/guidelines.xhtml

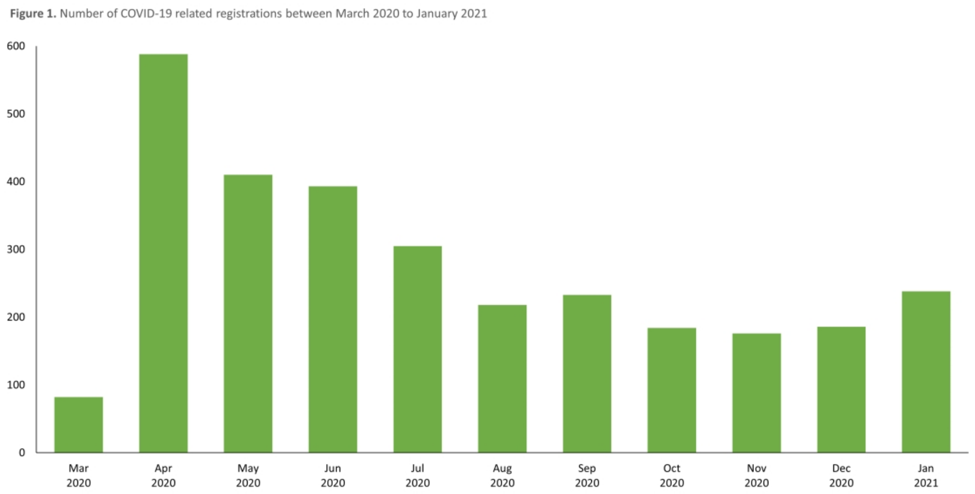

Figure 1. Number of COVID-19 related registrations between March 2020 to January 2021

817x428mm (130 x 130 DPI)

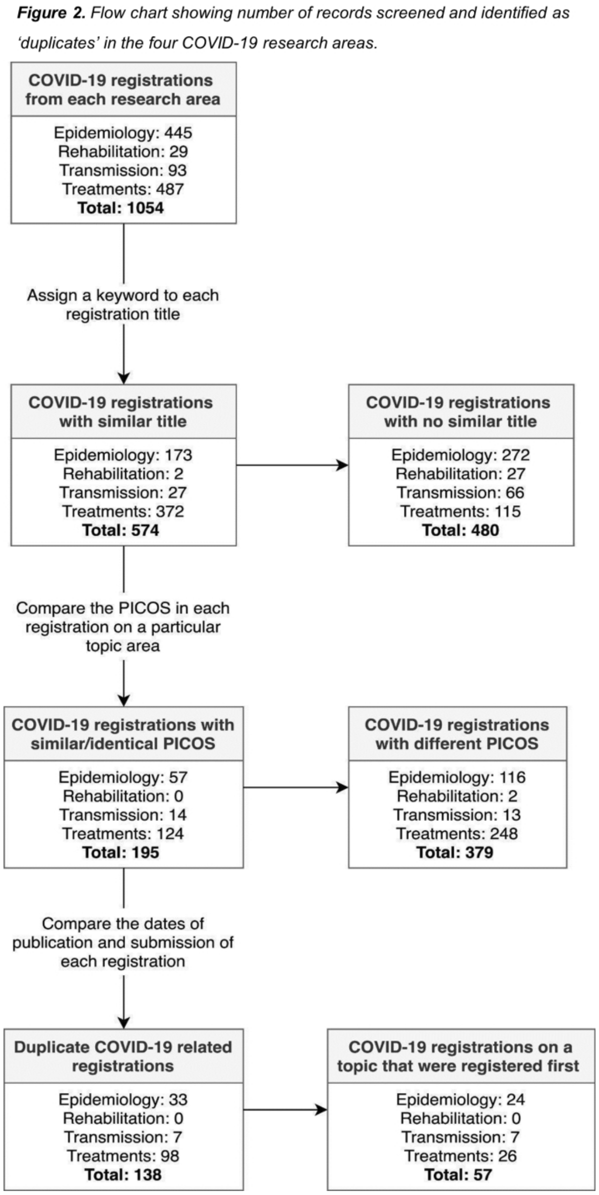

Figure 2. Flow chart showing number of records screened and identified as 'duplicates' in the four COVID-19 research areas.

287x582mm (130 x 130 DPI)

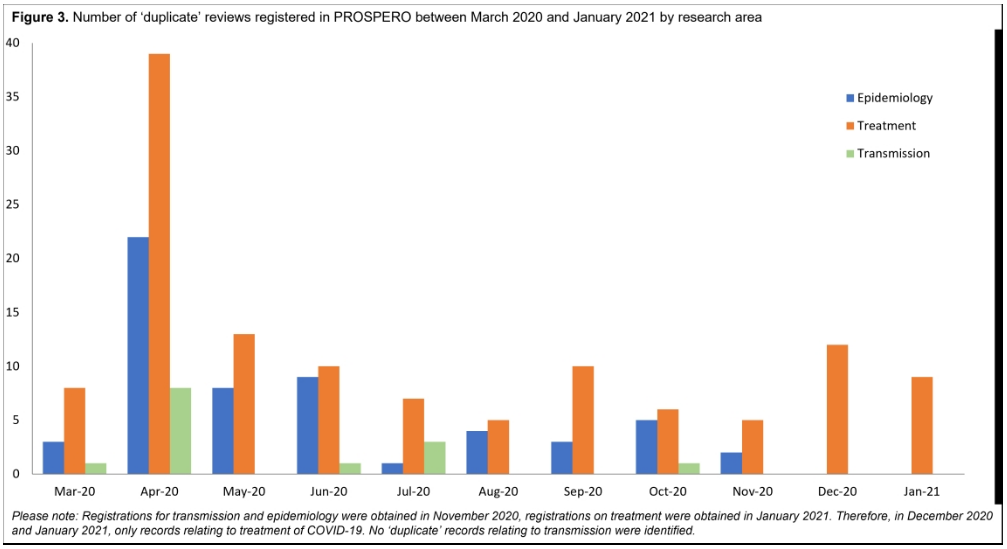

Figure 3. Number of 'duplicate' reviews registered in PROSPERO between March 2020 and January 2021 by research area

784x423mm (130 x 130 DPI)

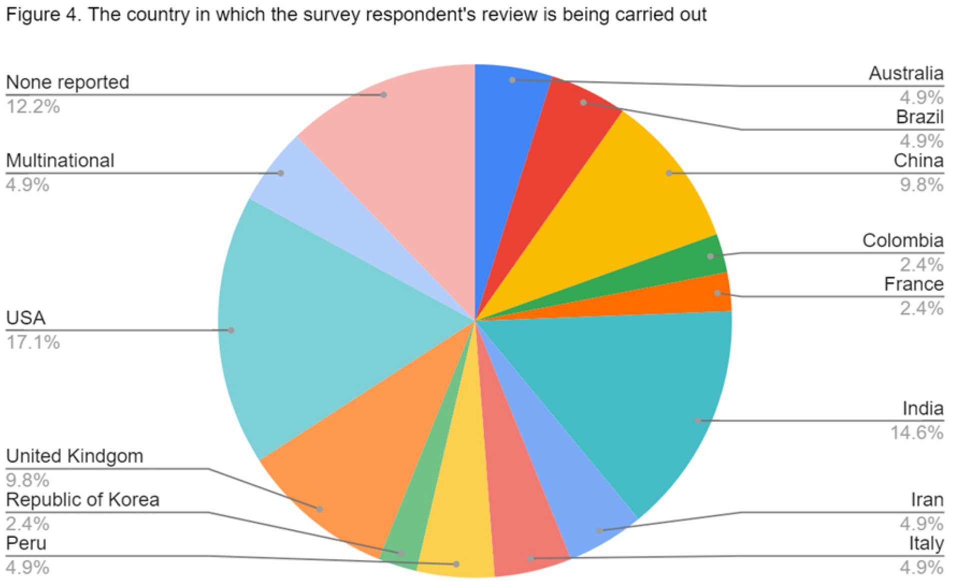

Figure 4. The country in which the survey respondent's review is/was being carried out.

622x385mm (38 x 38 DPI)

Appendix A

Search Terms:

'(((coronavirus or corona-virus) AND (wuhan or beijing or shanghai or Italy or South-Korea or korea or China or Chinese or 2019-nCoV or nCoV or COVID-19 or Covid19 or SARS-CoV* or SARSCov2 or ncov)) OR (pneumonia AND Wuhan) or "COVID-19" or "2019-nCoV" or "SARS-CoV" or SARSCOV2 or 2019-nCov or "2019 coronavirus" or "2019 corona virus" or covid19 or ncov OR "novel corona virus" or "new corona virus" or "nouveau corona virus" or "2019 corona virus" OR "novel coronavirus" or "new coronavirus" or "nouveau coronavirus" or "2019 coronavirus" or "Long Covid") NOT Animal:DB'

For peer review only

For peer review only

Appendix B

Data Extraction

Table 1. *Data extraction template for identifying duplicate reviews*

Record number	Research area	Date submitted	Date registered	Is the PICOS similar/identical to existing review?	Answer to screening question? *	Is the review funded?	Have authors acknowledged similar reviews?	Authors reason for duplication?	Is there a reason for duplication from our perspective?

**Screening questions before authors submit a PROSPERO registration ask whether their review is not similar to an existing review, sufficiently different, or similar to an existing review, but repetition is needed*

For peer review only

Appendix C

COVID-19 Systematic Review Duplication in PROSPERO Questionnaire

The survey can be entirely anonymous. But it would be useful for us to be able to link your responses to the PROSPERO record, so you can choose to include your personal information. If you do provide your identifying ID number, this will not be included or used in any future publications and it will not affect the status of your PROSPERO registration.

This survey has been granted ethical approval. If you choose to include your PROSPERO registration number, your data will be handled in compliance with GDPR and the UK Data Protection Act (2018). For more information, please visit the links on the University of York's policy on Data Protection Legislation, GDPR compliant research and the general privacy notice.

Q1 What is your PROSPERO registration number

____

About your COVID-19 related systematic review

Q2 Have you conducted a systematic review before?

- Yes (1)
- No (2)
- Other (3) ____

Q3 What research area was your systematic review looking at?

- Epidemiology (1)
- Transmission (2)
- Treatments (3)
- Other (4) ____

Q4 Why did you conduct your systematic review on a COVID-19 related topic?

- To inform future research that you had planned (such as a primary research study) (1)
- To inform funders/policy makers (2)
- For personal/departmental interest (3)
- Other (4) ____

Q5 Please provide details of the country in which the review is being carried out. For multi-national collaborations, give details of all the countries involved.

____

For peer review only

Q6 Is your review funded

- Yes (1)
- No (2)
- Other (3) ____

Display This Question:

If Is your review funded != No

Q7 If your review is funded, please provide details of the individuals, organizations, groups or other legal entities who take responsibility for initiating, managing, sponsoring and/or financing the review

____

Q8 Do you intend to publish or disseminate the results of your systematic review?

- Yes (1)
- No (2)
- Maybe (3)
- Other (4) ____

Display This Question:

If Do you intend to publish or disseminate the results of your systematic review? = No

Q9 Why did you decide not to publish the results of your systematic review?

____

Q10 Is there any other information that you wish to provide about your systematic review?

____

The following questions were specific to the registrant's response to the triage questions at PROSPERO submission

For peer review only

Not Similar Sub-Questions

When registering, users are prompted to search PROSPERO and check whether a similar systematic review has been registered. Some duplication, intended with good reason (e.g. the existing one is out of date or used suboptimal methods), is justifiable. When registering, you indicated that your review was not similar to an existing review

Q12 To help us understand this further, please indicate which statement applies most to your situation.

- I searched PROSPERO prior to registration and identified some/all of the similar records (1)
- I searched PROSPERO prior to registration but did not identify the similar records (2)
- I did not search PROSPERO prior to registration (3)

Display This Question:

If To help us understand this further, please indicate which statement applies most to your situation. = I searched PROSPERO prior to registration and identified some/all of the similar records

Q13 Please could you tell us more about the reasons you conducted a review similar to those which you identified?

____

Display This Question:

If To help us understand this further, please indicate which statement applies most to your situation. = I searched PROSPERO prior to registration but did not identify the similar records

Q14 Please could you tell us more about how you searched PROSPERO, including the search terms you used. This could help us to improve our guidance about how to search PROSPERO in the future.

____

Display This Question:

If To help us understand this further, please indicate which statement applies most to your situation. = I did not search PROSPERO prior to registration

Q15 Please could you tell us more about the reasons you did not search PROSPERO before registration?

____

Sufficiently Different Sub-Questions

When registering, users are prompted to search PROSPERO and check whether a similar systematic review has been registered. Some duplication, intended with good reason (e.g. the existing one is out of date or used suboptimal methods), is justifiable. When registering, you indicated that your review was similar to an existing review but sufficiently different to justify this review.

Q12 To help us understand this further, please indicate which statement applies most to your situation.

- I searched PROSPERO and identified some/ all of the existing reviews that were similar to my own. (1)
- Other (2)

Display This Question:

If To help us understand this further, please indicate which statement applies most to your situation. = I searched PROSPERO and identified some/ all of the existing reviews that were similar to my own.

Q13 For each of the similar reviews you identified, please could you tell us more about the reasons why your review is sufficiently different.

____

Display This Question:

If To help us understand this further, please indicate which statement applies most to your situation. = Other

Q14 Please could you tell us whether you searched PROSPERO, and if you did, please could you explain more about how you searched PROSPERO, including the search terms you used. This could help us to improve our guidance about how to search PROSPERO in the future.

____

For peer review only

Repetition Needed Sub-Questions

When registering, users are prompted to search PROSPERO and check whether a similar systematic review has been registered. Some duplication, intended with good reason (e.g. the existing one is out of date or used suboptimal methods), is justifiable. When registering, you indicated that your review was similar to an existing review but repetition was needed.

Q12 To help us understand this further, please indicate which statement applies most to your situation.

- I searched PROSPERO and identified some/ all of the existing reviews that were similar to my own. (1)
- Other (2)

Display This Question:

If To help us understand this further, please indicate which statement applies most to your situation. = I searched PROSPERO and identified some/ all of the existing reviews that were similar to my own.

Q13 For each of the similar reviews you identified, please could you tell us more about the reasons why repetition was needed.

____

Display This Question:

If To help us understand this further, please indicate which statement applies most to your situation. = Other

Q14 Please could you tell us whether you searched PROSPERO, and if you did, please could you explain more about how you searched PROSPERO, including the search terms you used. This could help us to improve our guidance about how to search PROSPERO in the future.

____

For peer review only

Appendix D

Research area	Date submitted	Date registered	Answer to screening question?	Have authors acknowledged similar reviews?	Authors reason for duplication?	Is there reason for duplication from our perspective?	Is the review funded?
**Cancer**	17/04/20	21/04/20	-	-	-	-	None
**Cancer**	22/04/20	22/04/20	NS	No	No	Perhaps, submitted one day after last was published	None
**Cardiovascular Disease**	14/04/20	20/04/20	-	-	-	-	None
**Cardiovascular Disease**	31/05/20	01/06/20	NS	No	No	No	National Natural Science Foundation of China
**Children**	15/04/20	21/04/20	-	-	-	-	None
**Children**	18/04/20	20/04/20	-	-	-	-	Nanchang University

For peer review only

Research area	Date submitted	Date registered	Answer to screening question?	Have authors acknowledged similar reviews?	Authors reason for duplication?	Is there reason for duplication from our perspective?	Is the review funded?
**Children**	02/08/20	06/08/20	RN*	No	-	Potentially. Four months since previous systematic review	None
**Comorbidities: Impact on Mortality**	24/03/20	03/04/20	-	-	-	-	None
**Comorbidities: Impact on Mortality**	22/05/20	28/05/20		No	None	No	None
**Comorbidities: Impact on Mortality**	29/10/20	30/10/20	NS*	No	None	Perhaps- to update evidence (5 months after last review).	None
**Comorbidities: Prevalence**	27/03/20	06/04/20	-	-	-	-	Human Resources Development Operational Programme Grant
**Comorbidities: Prevalence**	26/04/20	28/04/20	NS*	No	No	No	None

Research area	Date submitted	Date registered	Answer to screening question?	Have authors acknowledged similar reviews?	Authors reason for duplication?	Is there reason for duplication from our perspective?	Is the review funded?
**Comorbidities: Severe Outcomes and Death**	26/02/20	17/03/20	-	-	-	-	National Key R&D Program of China.
**Comorbidities: Severe Outcomes and Death**	26/03/20	16/04/20	SD	Yes	Yes: no risk of bias in CRD42020170315	Yes, CRD42020170315 do not report methods of quality assessment).	None
**Comorbidities: Severe Outcomes and Death**	31/03/20	30/04/20	NS	No	None	No	None
**Comorbidities: Severe Outcomes and Death**	13/04/20	16/04/20	NS	No	None	No	Second Affiliated Hospital of Nanchang University
**COVID-19 in Healthcare Workers**	06/04/20	15/04/20	--	-	-	-	None
**COVID-19 in Healthcare Workers**	27/04/20	28/04/20	NS	None	No	No	None
**Diabetes**	28/04/20	04/05/20	-	-	-	-	None

Research area	Date submitted	Date registered	Answer to screening question?	Have authors acknowledged similar reviews?	Authors reason for duplication?	Is there reason for duplication from our perspective?	Is the review funded?
**Diabetes**	30/06/20	30/06/20	NS	No	None	No	Natural Science Foundation of Tianjan and Tianjan Education Committee
**Diabetes**	05/10/20	06/10/20	NS	No	None	Perhaps update of review potentially needed	None
**Echocardiographic Findings**	18/08/20	19/08/20	-	-	-	-	None
**Echocardiographic Findings**	19/09/20	22/09/20	NS	No	No	No	None
**Face Masks**	29/09/20	29/09/20	-	-	-	-	None
**Face Masks**	19/10/20	28/10/20	SD	No	No	Yes. Living systematic review	None
**General Risk Factors: Impact on Mortality**	26/03/20	03/04/20	-	-	-	-	None

Research area	Date submitted	Date registered	Answer to screening question?	Have authors acknowledged similar reviews?	Authors reason for duplication?	Is there reason for duplication from our perspective?	Is the review funded?
**General Risk Factors: Impact on Mortality**	04/04/20	09/04/20	NS	None	No	Potentially- only one day after previous record was registered.	Wellcome Trust
**General Risk Factors: Impact on Poor Outcomes and Mortality**	27/02/20	03/04/20	-	-	-	-	National Natural Science Foundation
**General Risk Factors: Impact on Poor Outcomes and Mortality**	15/03/20	27/03/20	-	-	-	-	None
**General Risk Factors: Impact on Poor Outcomes and Mortality**	18/03/20	23/03/20	NS	None	No	No	Chinese Natural Science Foundation and the Training Program of Shanghai Health and Family Planning System
**General Risk Factors: Impact on Poor Outcomes and Mortality**	28/03/20	09/04/20	SD	Yes	Yes, provide additional analysis (regression)	Yes	None

For peer review only - http://bmjopen.bmj.com/site/about/guidelines.xhtml

Research area	Date submitted	Date registered	Answer to screening question?	Have authors acknowledged similar reviews?	Authors reason for duplication?	Is there reason for duplication from our perspective?	Is the review funded?
**General Risk Factors: Impact on Poor Outcomes and Mortality**	12/04/20	16/04/20	NS	No	None	No	None
**General Risk Factors: Impact on Poor Outcomes and Mortality**	14/04/20	17/05/20	NS*	No	None	None	None
**General Risk Factors: Impact on Poor Outcomes and Mortality**	20/04/20	21/04/20	RN	No	None	None	None
**General Risk Factors: Impact on Poor Outcomes and Mortality**	06/05/20	06/05/20	SD	No	None	No	Department of Epidemiology, College of Public Health, University of Nebraska Medical
**General Risk Factors: Impact on Poor Outcomes and Mortality**	11/05/20	12/05/20	NS	No	None	No	None
**Long COVID**	15/10/20	23/10/20	-	-	-	-	None

Research area	Date submitted	Date registered	Answer to screening question?	Have authors acknowledged similar reviews?	Authors reason for duplication?	Is there reason for duplication from our perspective?	Is the review funded?
**Long COVID**	31/10/20	02/11/20	SD	No	No	No	None
**Manifestations: Taste and Smell**	22/05/20	29/05/20	-	-	-	-	None
**Manifestations: Taste and Smell**	20/06/20	25/06/20	NS	No	None	None	Health Commission of Hunan Province and Human Pharmaceutical Association
**Mental Health in Healthcare Workers**	31/07/20	03/08/20	-	-	-	-	National Council for Scientific and Technological Development - CNPq, Brazilian Government
**Mental Health in Healthcare Workers**	19/10/20	22/10/20	NULL	No	No	No	None
**Neurological Manifestations**	22/05/20	01/06/20	-	-	-	-	None
**Neurological Manifestations**	02/06/20	02/06/20	NS	No	None	Perhaps, submitted one day after previous was published	None

For peer review only

Research area	Date submitted	Date registered	Answer to screening question?	Have authors acknowledged similar reviews?	Authors reason for duplication?	Is there reason for duplication from our perspective?	Is the review funded?
**Obesity**	13/04/20	06/04/20	-	-	-	-	NIHR Applied Research and Care
**Obesity**	29/04/20	01/05/20	NS	None	None	No	University of Indonesia
**Obesity**	13/05/20	15/05/20	NS	No	None	None	Programme Széchenyi 20
**Obesity**	10/11/20	12/11/20	SD	No	No	Perhaps update of review potentially needed	None
**Pregnancy**	04/04/20	23/06/20	-	-	-	-	Partly funded by WHO
**Pregnancy**	13/04/20	17/04/20	-	-	-	-	None
**Pregnancy**	24/04/20	24/04/20	NS*	No	None	No	None
**Skin Manifestations**	23/06/20	25/06/20	-	-	-	-	None

Research area	Date submitted	Date registered	Answer to screening question?	Have authors acknowledged similar reviews?	Authors reason for duplication?	Is there reason for duplication from our perspective?	Is the review funded?
**Skin Manifestations**	28/06/20	29/06/20	SD	Yes	Yes (none will be published before completion)	No	None
**Treatment: ACE Inhibitors and ARBs**	07/06/20	19/06/20	-	-	-	-	Major Project of National Science and Technology on New Drug etc.
**Treatment: ACE Inhibitors and ARBs**	03/07/20	08/07/20	SD	No	None	No	None
**Treatment: Metformin**	21/08/20	25/08/20	-	-	-	-	ARC-EM
**Treatment: Metformin**	12/09/20	15/09/20	RN	None	None	No	National Natural Science China
**Breastfeeding**	06/04/20	21/04/20	SD	References CRD420173886	Yes. But review is sufficiently so no duplication occurred.	Yes	None
**Breastfeeding**	11/04/20	20/04/20	N/A	-	-	-	None
**Breastfeeding**	17/07/20	20/07/20	SD	Not explicitly	No	No	None

Research area	Date submitted	Date registered	Answer to screening question?	Have authors acknowledged similar reviews?	Authors reason for duplication?	Is there reason for duplication from our perspective?	Is the review funded?
**Breastfeeding**		10/10/20	NS	No	No	No comparator.	None
**Maternal to Fetal Transmission**	16/03/20	24/03/20	NS	-	-	-	N/A
**Maternal to Fetal Transmission**	22/06/20	03/07/20	SD	No	No	No	None
**Surgery**	02/04/20	22/04/20	NS	-	-	-	None
**Surgery**	25/04/20	28/04/20	NS	No	N/A	No	None
**Viral Shedding**	24/04/20	29/04/20	NS	-	-	-	None
**Viral Shedding**	22/06/20	22/06/20	NS*	Yes, acknowledge CRD420181914	Yes, previous registration deemed poor quality	Potentially- poor reporting in previous registration. Authors have given reason for duplication.	None
**Weather**	28/03/20	07/04/20	NS	-	-	-	None

Research area	Date submitted	Date registered	Answer to screening question?	Have authors acknowledged similar reviews?	Authors reason for duplication?	Is there reason for duplication from our perspective?	Is the review funded?
**Weather**	01/04/20	08/04/20	NS	-	-	-	Federal University of Pará, Brazil.
**Weather**	27/04/20	30/04/20	NS	No	No	Yes, looking at seasons and times of year rather than climate conditions.	None
**Weather**	03/07/20	03/07/20	SD	No	No	Almost identical. No real reason for duplication.	None
**Ace inhibitors**	05/04/20	14/04/20	NS	-	-	-	Self funded
**Ace inhibitors**	13/04/20	17/04/20	NS*	-	-	-	None
**Ace inhibitors**	03/05/20	05/05/20	NS	No	NO	NO	None

Research area	Date submitted	Date registered	Answer to screening question?	Have authors acknowledged similar reviews?	Authors reason for duplication?	Is there reason for duplication from our perspective?	Is the review funded?
**Ace inhibitors**	04-Mayx-20	04/05/20	NS	No	NO	NO	China Postdoctoral Science Foundation (2019M652593) and Henan Postdoctoral Science Foundation (1902006)
**Ace inhibitors**	08/05/20	11/05/20	SD	Partially	NO	NO	None
**Ace inhibitors**	13/05/20	14/05/20	NS	No	NO	NO	This study was supported by National Natural Science Foundation of China, 81970203; National Natural Science Foundation of China, 81570212; National Natural Science Foundation of China, 31800976 Chongqing Science and Health Joint Medical Research Project, 2018QNXM024
**Ace inhibitors**	27/07/20	28/07/20	NS	No	NO	NO	Qingsong Qin is sponsored by Department of education of Guangdong province, China

Research area	Date submitted	Date registered	Answer to screening question?	Have authors acknowledged similar reviews?	Authors reason for duplication?	Is there reason for duplication from our perspective?	Is the review funded?
**Ace inhibitors**	03/08/20	04/08/20	SD	Partially	NO	NO	Disciplines Construction Project of PUMC (Grant number: 20192102). Chinese Academy of Medical Sciences, CAMS Innovation Fund for Medical Sciences (Grant number: 2018-I2M-1-002)
**All treatment living**	20/04/20	22/04/20	SD	-	-	-	None
**All treatment living**	27/04/20	28/04/20	SD	Partially	NO	No	This review received some funding from the Agence Nationale de la Recherche (ANR) and from the WHO
**All treatment MA**	07-Feb-20	31/03/20	NS	-	-	-	NA

For peer review only

Research area	Date submitted	Date registered	Answer to screening question?	Have authors acknowledged similar reviews?	Authors reason for duplication?	Is there reason for duplication from our perspective?	Is the review funded?
**All treatment MA**	09/03/20	20/03/20	NS	No	No	No	This study will be funded by Research Funds of Shanghai Health and Family Planning commission (20184Y0022, 20194Y0007), Cultivation fund of clinical research of Renji hospital (PY2018-III-06), Clinical Pharmacy Innovation Research Institute of Shanghai Jiao Tong University School of Medicine (CXYJY2019ZD001, CXYJY2019QN004).
**All treatment MA**	22/03/20	24/03/20	NS	No	No	No	Shubham Misra is a recipient of DST-INSPIRE PhD Fellowship from Department of Science & Technology, Government of India
**All treatment MA**	07/04/20	17/04/20	NS	No	No	No	https://covid19.kacst.edu.sa/grants/

Research area	Date submitted	Date registered	Answer to screening question?	Have authors acknowledged similar reviews?	Authors reason for duplication?	Is there reason for duplication from our perspective?	Is the review funded?
**All treatment MA**	20/04/20	22/04/20	SD	Partially	No	No	None
**All treatment MA**	05/05/20	06/05/20	NS	No	No	No	None
**All treatment MA**	13/10/20	16/10/20	NS	No	No	No	This work was funded by the Guidance Plan for Social Development of Taizhou Municipal Science and Technology (ssf20160141).
**All treatment MA**	14/11/20	07/12/20	NS	No	No	No	None
**All treatment MA**	05/01/21	08/01/21	Not in system	No	No	No	None
**All treatment NMA**	07/04/20	15/04/20	NS	NO	Yes		
**All treatment NMA**	12/04/20	16/04/20	SD	Partially	NA		
**All treatment NMA**	18/04/20	22/04/20	SD	Partially	No	No	The research project will be assisted with the by Griffith University

Research area	Date submitted	Date registered	Answer to screening question?	Have authors acknowledged similar reviews?	Authors reason for duplication?	Is there reason for duplication from our perspective?	Is the review funded?
							Postgraduate Research Scholarship
**All treatment NMA**	19/04/20	20/04/20	NS	NO	No	No	None
**All treatment NMA**	23/04/20	23/04/20	NS	NO	No	No	Supported by the Programme of Introducing Talents of Discipline to Universities, China, the 111 Project, D18011)
**All treatment NMA**	27/04/20	27/04/20	SD	Partially	No	No	None
**All treatment NMA**	15/05/20	19/05/20	NS	NO	No	No	None
**All treatment NMA**	18/06/20	24/06/20	NS	NO	No	No	None
**All treatment NMA**	18/10/20	26/10/20	NS	NO	No	No	None
**All treatment umbrella**	13/05/20	20/05/20	NS	NA	NA	NA	NA

Research area	Date submitted	Date registered	Answer to screening question?	Have authors acknowledged similar reviews?	Authors reason for duplication?	Is there reason for duplication from our perspective?	Is the review funded?
**All treatment umbrella**	07/09/20	07/09/20	NS	No	No	No	Indian Council of Medical Research
**All treatment umbrella**	04/10/20	05/10/20	NS	No	No	No	None
**Anticoagulant**	02/06/20	02/06/20	NS	-	-	-	
**Anticoagulant**	02/07/20	03/07/20	SD	Partially	No	No	None
**Anticoagulant**	14/10/20	15/10/20	NS	No	No	No	None
**Anticoagulant**	07/01/21	11/01/21	not in list	No	No	No	None
**Blood plasma**	12/03/20	24/03/20	NS	-	-	-	None
**Blood plasma**	28/03/20	08/04/20	SD	Partially		No reasons why sufficiently different.	Funded by the UK Forum
**Blood plasma**	02/04/20	02/04/20	NS	No	No	No	None

Research area	Date submitted	Date registered	Answer to screening question?	Have authors acknowledged similar reviews?	Authors reason for duplication?	Is there reason for duplication from our perspective?	Is the review funded?
**Blood plasma**	06/04/20	15/04/20	SD	Partially	No	No reasons why sufficiently different.	None
**Blood plasma**	10/04/20	15/04/20	NS	No	No	No	Education Foundation of Chengdu University of Traditional Chinese Medic
**Blood plasma**	17/06/20	19/06/20	SD	Partially	No	No	None
**Blood plasma**	05/07/20	06/07/20	SD	Partially	No	No reasons why sufficently different.	None
**Blood plasma**	16/07/20	16/07/20	NS	No	No	No	None
**Blood plasma**	12/08/20	14/08/20	SD	Partially	No	No reasons why sufficently different.	None
**Blood plasma**	09/12/20	22/12/20	not in list	No	No	No	None
**Blood plasma**	16/12/20	17/12/20	not in list		No	No	None

Research area	Date submitted	Date registered	Answer to screening question?	Have authors acknowledged similar reviews?	Authors reason for duplication?	Is there reason for duplication from our perspective?	Is the review funded?
**Blood plasma**	21/12/20	22/12/20	not in list	No	Potentially severe patient pop.	No	None
**Colchicine**	17/09/20	21/09/20					None
**Colchicine**	11/01/21	12/01/21	not in list	No	No	No	None
**Corticosteroids**	03/03/20	20/04/20	NS	-	-	-	None
**Corticosteroids**	20/04/20	21/04/20	NS	-	-	-	None
**Corticosteroids**	28/04/20	30/04/20	NS	No	No	No	None
**Corticosteroids**	06/05/20	06/05/20	SD	Partially	No	No	This work was supported by National Key Research and Development Project (20YFC2005700), Key Realm R&D Program of Guangdong Province (2019B030335001), and Medical Scientific Research

Research area	Date submitted	Date registered	Answer to screening question?	Have authors acknowledged similar reviews?	Authors reason for duplication?	Is there reason for duplication from our perspective?	Is the review funded?
							Foundation of Guangdong Province of China (A2019407)
**Corticosteroids**	07/07/20	07/07/20	SD	Partially	No	No	None
**Corticosteroids**	23/09/20	23/09/20	SD	Partially	No	No	None
**Corticosteroids**	07/12/20	08/12/20	not in list	No	No	No	None
**Corticosteroids**	21/12/20	22/12/20	not in list	No	No	No	NaFoUniMedCovid19“ (FKZ: 01KX21) „CEO-Sys“
**Corticosteroids**	05/01/21	12/01/21	not in list	No	No	No	None
**ECMO**	02/04/20	14/04/20		-	-	-	None

Research area	Date submitted	Date registered	Answer to screening question?	Have authors acknowledged similar reviews?	Authors reason for duplication?	Is there reason for duplication from our perspective?	Is the review funded?
**ECMO**	07/04/20	14/04/20		-	-	-	None
**ECMO**	16/04/20	17/04/20	NS	No	No	No	None
**ECMO**	03/05/20	04/05/20	SD	Partially	No	No	None
**ECMO**	15/06/20	18/06/20	NS	No	No	No	None
**ECMO**	19/06/20	22/06/20	NS	No	No	No	None
**ECMO**	17/09/20	21/09/20	NS	No	No	No	None
**ECMO**	22/11/20	23/11/20	NS	No	No	No	This work was supported by the research Grant 20YFC0841300 from Ministry of Science and Technology of the People's Republic of China.
**ECMO**	21/12/20	22/12/20	NS	No	No	No	None

Research area	Date submitted	Date registered	Answer to screening question?	Have authors acknowledged similar reviews?	Authors reason for duplication?	Is there reason for duplication from our perspective?	Is the review funded?
**ECMO**	04/01/21	07/01/21	NS	No	No	No	None
**Hydroxychloroquine**	22-Feb-20	17/03/20		-	-	-	None
**Hydroxychloroquine**	29/03/20	16/04/20	NS	No	No	No	Major Projects of Science and Technology Innovation 2025 in Ningbo, China
**Hydroxychloroquine**	04/04/20	06/04/20	SD	Partially	No	No	NIHR Applied Research Collaboration East Midlands (ARC EM), UK
**Hydroxychloroquine**	06/04/20	07/04/20	SD	Partially	No	No	None
**Hydroxychloroquine**	06/04/20	09/04/20	NS	No	No	No	None
**Hydroxychloroquine**	07/04/20	17/04/20	NS	No	No	No	None
**Hydroxychloroquine**	10/04/20	21/04/20	SD	Partially	No	No	None
**Hydroxychloroquine**	14/04/20	17/04/20	SD	Partially	No	No	None

Research area	Date submitted	Date registered	Answer to screening question?	Have authors acknowledged similar reviews?	Authors reason for duplication?	Is there reason for duplication from our perspective?	Is the review funded?
**Hydroxychloroquine**	20/04/20	21/04/20	SD	Partially	No	No	None
**Hydroxychloroquine**	27/04/20	28/04/20	NS	No	No	No	None
**Hydroxychloroquine**	22/05/20	22/05/20	SD	Partially	No	No	None
**Hydroxychloroquine**	10/08/20	12/08/20	NS	No	No	No	None
**Hydroxychloroquine**	19/08/20	19/08/20	NS	No	No	No	None
**Hydroxychloroquine**	12/09/20	17/09/20	NS	No	No	No	None
**Hydroxychloroquine**	12/09/20	15/09/20	SD	Partially	No	No	None
**Interferon**	26/03/20	26/03/20	NA	-	-	-	None
**Interferon**		07/04/20	NA	-	-	-	None
**Interferon**	17/11/20	20/11/20	SD	Partially	No	No	None

Research area	Date submitted	Date registered	Answer to screening question?	Have authors acknowledged similar reviews?	Authors reason for duplication?	Is there reason for duplication from our perspective?	Is the review funded?
**Interferon**	21/11/20	03/12/20	NS	No	No	No	None
**Ivermectin**	08/07/20	09/07/20		-	-	-	-
**Ivermectin**	20/10/20	09/11/20	NS	no	No	No	None
**Ivermectin**	24/12/20	07/01/21	NS	no	No	No	The Institute for Health Technology Assessment (IETS),
**Ivermectin**	11/01/21	12/01/21	NS	no	No	No	None
**JAK inhibitors**	07/06/20	09/06/20		-	-	-	NA
**JAK inhibitors**	04/01/21	12/01/21	SD	partially	no	no	None
**lopinavir**	06/04/20	15/04/20	NA	NA	NA	NA	NA
**lopinavir**	09/04/20	15/04/20	NA	NA	NA	NA	NA

Research area	Date submitted	Date registered	Answer to screening question?	Have authors acknowledged similar reviews?	Authors reason for duplication?	Is there reason for duplication from our perspective?	Is the review funded?
**lopinavir**	12/04/20	16/04/20	NA	NA	NA	NA	NA
**lopinavir**	17/04/20	21/04/20	NS	no	no	no	None
**lopinavir**	24/04/20	24/04/20	NS	no	no	no	None
**lopinavir**	13/01/21	15/01/21	SD	partially	no	no	This study is carried out at the request of the Ministry of Health and Social Protection and the Disaster Risk Management Fund. Contract No. 9677-MECOVID19-1009-20.
**Prone position**	24/06/20	24/06/20	NA	-	-	-	-
**Prone position**	28/08/20	01/09/20	NS	no	no	no	None
**Prone position**	11/09/20	14/09/20	NS	no	no	no	None
**Prone position**	19/10/20	22/10/20	NS	no	no	no	None

Research area	Date submitted	Date registered	Answer to screening question?	Have authors acknowledged similar reviews?	Authors reason for duplication?	Is there reason for duplication from our perspective?	Is the review funded?
**Prone position**	16/11/20	20/11/20	RN	yes	no	no	None
**Prone position**	10/12/20	11/12/20	NS	no	no	no	None
**Prone position**	12/12/20	14/12/20	NS	no	no	no	None
**RAAS**	07/05/20	11/05/20		-	-	-	None
**RAAS**	14/06/20	17/06/20	NS	no	no	no	None
**RAAS**	15/09/20	16/09/20	RN	yes	no	no	None
**Remdisivir**	08-Feb-20	23/03/20	NS	-	-	-	National Natural Science Foundation of China
**Remdisivir**	02/04/20	14/04/20	NS	No	No	No	CDT-Africa, College of Health Sciences, Addis Ababa University
**Remdisivir**	21/04/20	23/04/20	NS	No	No	No	None
**Remdisivir**	30/04/20	01/05/20	SD	No	No	No	None

Research area	Date submitted	Date registered	Answer to screening question?	Have authors acknowledged similar reviews?	Authors reason for duplication?	Is there reason for duplication from our perspective?	Is the review funded?
**Remdisivir**	26/05/20	26/05/20	NS	No	No	No	Tongji Hospital, Tongji Medical College
**Remdisivir**	20/09/20	21/09/20	NS	No	No	No	None
**Remdisivir**	12/10/20	15/10/20	NS	No	No	No	Zhejiang Medicine and Health Science and Technology Project; Natural Science Foundation of Zhejiang Province
**Tocilizumab**	22/03/20	24/03/20	NS	-	-	-	None
**Tocilizumab**	18/06/20	19/06/20	NS		No	No	None
**Tocilizumab**	21/06/20	22/06/20	SD		No	No	None
**Tocilizumab**	26/06/20	01/07/20	NS		No	No	Nicola Luigi Bragazzi and Jianhong Wu are partially supported by the Canadian Institute of Health Research

Research area	Date submitted	Date registered	Answer to screening question?	Have authors acknowledged similar reviews?	Authors reason for duplication?	Is there reason for duplication from our perspective?	Is the review funded?
							(CIHR) 2019 Novel Coronavirus (COVID-19) rapid research program
**Tocilizumab**	14/08/20	17/08/20	NS		No	No	None
**Tocilizumab**	06/11/20	10/11/20	NS	no	No	No	None
**Tocilizumab**	11/12/20	18/12/20	NS	no	No	No	1.3.5 project for disciplines of excellence, West China Hospital, Sichuan University
**Tocilizumab**	16/12/20	17/12/20	SD	Partially	No	No	None

* Registrant changed their answer to the screening question. Grey cells represent ‘duplicate’ records, white cells represent records registered first.
Abbreviations: NS, Not Similar; RN, Repetition Needed; SD, Sufficiently Different.

Appendix E

Duplicate Registrations Characteristics

Table 2: Number of 'duplicate' records registered in each COVID-19 related topic area.

Topic	N duplicates
**Epidemiology**	
**Health Condition and Impact on Outcome**	
Cancer	1
Cardiovascular disease	1
Diabetes	2
General Risk Factors	8
Obesity	3
Comorbidities	6
**Healthcare Workers**	
COVID-19 in healthcare workers	1
Mental Health in Healthcare Workers	1
**Clinical presentation of COVID-19**	
Echocardiographic Findings	1
Manifestations (Dermatological)	1
Manifestations (Taste and Smell)	1
Manifestations (Neurological)	1
Long-COVID	1
**Transmission of COVID-19**	
Facemasks	1
Pregnancy and mother-baby transmission	1
**Treatment of COVID-19**	
ACE-inhibitors	1
Metformin	1
**Total number of duplicates**	**31**
**Treatments**	
**All treatments**	
All treatment living	1
All treatment MA	7
All treatment umbrella	2
All treatment NMA	8
**Pharmacological interventions**	
ACE-inhibitors	6
Anticoagulants	1
Corticosteroids	4
Hydroxychloroquine	14
Interferon	2

Ivermectin	3
JAK inhibitors	1
**Topic**	**N duplicates**
**Pharmacological interventions**	
lopinavir	3
RAAS	2
Remdisivir	6
Tocilizumab	7
**Non-pharmacological interventions**	
Blood plasma	8
ECMO	5
Prone position	6
**Total number of duplicates**	**86**
**Transmission**	
Mother-baby transmission	2
Transmission during Surgery	1
Weather	1
**Total number of duplicates**	**4**

For peer review only

For peer review only
